# Chemical and Microstructural Nanoscale Homogeneity in Superconducting YBa_2_Cu_3_O_7–*x*_ Films Derived from Metal-Propionate Fluorine-free Solutions

**DOI:** 10.1021/acsami.2c11414

**Published:** 2022-10-21

**Authors:** Lavinia Saltarelli, Kapil Gupta, Silvia Rasi, Aiswarya Kethamkuzhi, Albert Queraltó, Diana Garcia, Joffre Gutierrez, Jordi Farjas, Pere Roura-Grabulosa, Susagna Ricart, Xavier Obradors, Teresa Puig

**Affiliations:** † Institut de Ciència de Materials de Barcelona, ICMAB-CSIC, Campus de la UAB, 08193 Bellaterra, Catalonia, Spain; ‡ GRMT, Department of Physics, University of Girona, E17071 Girona, Catalonia, Spain

**Keywords:** chemical solution deposition, fluorine-free, superconducting materials, transient liquid-assisted growth, YBa_2_Cu_3_O_7−x_

## Abstract

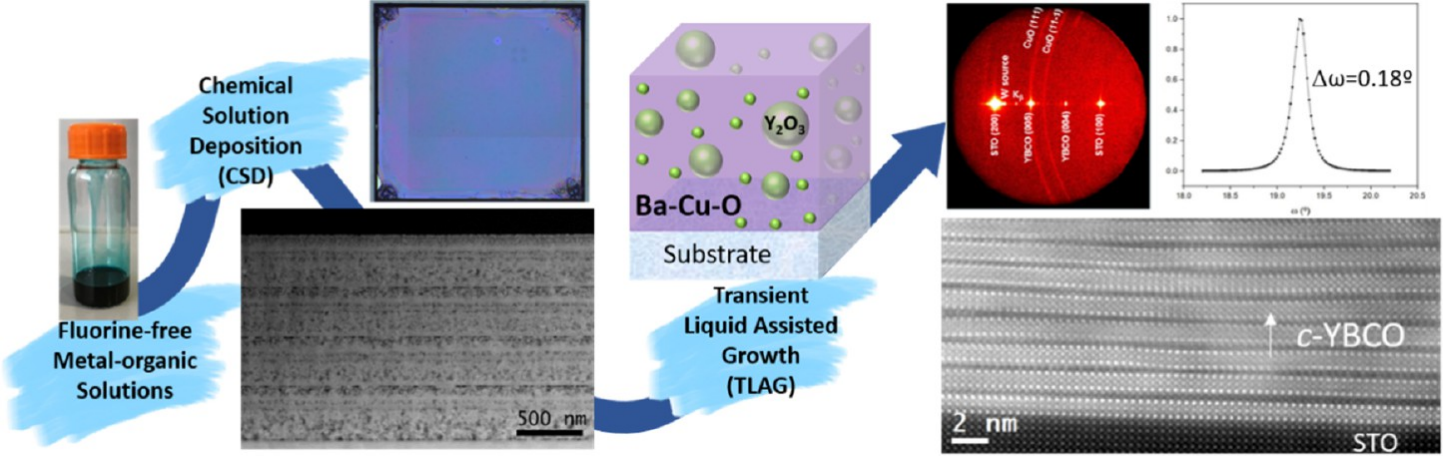

Research involved in developing alternative energy sources has become a necessity to face global warming. In this context, superconductivity is an appealing solution to enhance clean electrical energy provided that lower production costs can be attained. By implementation of chemical solution deposition techniques and high-throughput growth methods, low-cost nanostructured epitaxial cuprate superconductors are timely candidates. Here, we present a versatile and tunable solution method suitable for the preparation of high-performance epitaxial cuprate superconducting films. Disregarding the renowned trifluoroacetate route, we center our focus on the transient liquid-assisted growth (TLAG) that meets the requirement of being a greener chemical process together with ultrafast growth rates beyond 100 nm/s. We developed a facile, fast, and cost-effective method, starting from the synthesis of metal-propionate powders of Y, Ba, and Cu of high purity and high yields, being the precursors of the fluorine-free solutions, which enable the chemical and microstructural nanoscale homogeneity of YBa_2_Cu_3_O_7–*x*_ (YBCO) precursor films. These solutions present endured stability and enable precise tunability of the composition, concentration, porosity, and film thickness. Homogeneous precursor films up to thicknesses of 2.7 μm through eight layer multidepositions are demonstrated, thus establishing the correct basis for epitaxial growth using the fast kinetics of the TLAG process. YBCO films of 500 nm thickness with a critical current density of 2.6 MA/cm^2^ at 77 K were obtained, showing the correlation of precursor film homogeneity to the final YBCO physical properties.

## Introduction

The development of alternative energy sources has experienced an exponential progress in the past decades, given the necessity of reducing the environmental impact of years of fossil fuel energy source exploitation. Superconducting materials opened new opportunities to solve the problem of efficient electricity transport as they uniquely display no losses in large current transport.^1−3^ The discovery of high-temperature superconductors (HTS) pushed forward this technology, although many difficulties had to be overcome.^4,5^ Nowadays, HTS are promising candidates for various applications not only in clean power energy devices, such as power cables, fault current limiters, and transformers but also for equipment working at ultrahigh magnetic fields (accelerators, NMR, fusion reactors) and transport (ships, levitating trains, electrical airplanes)^6^ as well as in thermoelectric technology.^7−11^ Successive to the discovery of HTS, their widespread implementation was delayed by the need to develop suitable fabrication methods for superconducting REBa_2_Cu_3_O_7–*x*_ (REBCO, RE = Y, rare earth element) materials,^12^ making them extremely appealing for various applications due to the unprecedented high value of the irreversibility line this system presents.^4,13,14^

The introduction of HTS on flexible substrate architectures appropriate for industrialization, specifically coated conductors (CCs), boosted their production spread, with successful production of hundreds of meters long pieces with current capacities in the range 500–700 A/(cm × width) of tape.^4,13,15−18^ The development of CCs required adapting a thin-film technology to a kilometer length application, with epitaxial multilayers deposited on a long-length flexible metallic substrate. This breakthrough would define the widespread use of this technology, which, however, is still in its dawning given the extremely high production costs of the currently available technology.^2,13^ The techniques commonly employed for the growth of the HTS layer are pulsed laser deposition (PLD), metal–organic chemical vapor deposition (MOCVD), evaporation, and trifluoroacetate metal–organic decomposition–chemical solution deposition (TFA-CSD),^19^ which mostly imply expensive vacuum equipment and/or slow diffusion kinetics. Being vapor-based or gas–solid diffusion techniques, the low growth rates limit a cost-effective and high-throughput manufacturing of the material.^20,21^

For this reason, research involved in low-cost fabrication of YBCO superconductors based on chemical methods, disregarding the need of expensive vacuum systems, led to an innovative approach beyond the traditional TFA-CSD.^19,22^ Environmentally friendly non-fluorinated precursors for the preparation of YBCO solutions were introduced, despite the need to overcome the decomposition of BaCO_3_ (an intermediate compound in YBCO formation, with its elimination being the limiting step of the reaction) at low temperatures.^19^ Recently, we demonstrated^23^ that using fluorine-free solutions in a kinetically driven CSD approach, fabrication of YBCO layers could be attained at growth rates beyond 100 nm/s. This method, known as transient liquid-assisted growth (TLAG) process, promotes a cost-effective, high-throughput, and scalable growth of CC manufacturing, which can also benefit from combinatorial approaches to accelerate the experimentation and development of films with high performance.^24−26^ The novelty of TLAG lies in the fact that it employs a two-step nucleation mechanism in non-equilibrium conditions, starting from the formation of a transient liquid of Ba–Cu–O in which solid Y_2_O_3_ nanoparticles are dispersed, which occurs in a region of the phase diagram in which, instead, solid phases (like YBCO) are energetically favored. The Y_2_O_3_ dissolution in the transient liquid and the high atomic mobility of Y in the liquid favor Y diffusion toward the nucleation front of YBCO nuclei and, thus, its ultrafast epitaxial growth through a liquid–solid reaction.^23,27,28^ The driving force of this process is the Y supersaturation in the transient liquid, a parameter strongly controlled by the composition of the latter and easily modified through the stoichiometry of the precursor solutions. With TLAG being a liquid-assisted method, it disregards previously known solid–solid reaction mechanisms described in the literature, including solid–solid YBCO growth, a mechanism widely accepted for the TFA-MOD route,^19^ and other solid–solid crystal growth scenarios, in which precursor crystallinity dictates the crystalline phase of the final product.^29^ As in TLAG, the precursor phases react to form a transient liquid; the loss of crystallinity in this step of the growth process results in YBCO nucleation, showing no phase memory with its nanocrystalline precursors. However, the requirements for the homogeneity of the nanocrystalline precursor phases are essential for the YBCO nucleation and its ultrafast epitaxial growth, and a strict control on the formation of the precursor phases is key to high-performance superconducting films. The unique capabilities and potential of TLAG-CSD demonstrated an ultrafast growth rate of 100 nm/s, its compatibility with nanocomposite growth, and high critical current densities (3–5 MA/cm^2^ at 77 K self-field) in films of 100 nm in thickness.^23^ The knowledge acquired from the previous acetate-based fluorine-free solutions has been beneficial for the fundamental understanding of the precursors’ role in the TLAG process.^24,25^ Further steps in the advancement of TLAG require higher film thicknesses while maintaining the robustness of the process. Here, we describe the development of a novel class of solutions based on fluorine-free pure metal propionates, which is low-cost, robust, scalable, and suitable for TLAG-CSD growth of epitaxial YBCO superconducting films of high thickness with reproducible results. Stable solutions could be reached for various Y/Ba/Cu compositions, achieving chemical and microstructural nanoscale homogeneity for precursor films. This enables fine-tuning of the supersaturation conditions during TLAG not only by varying the growth conditions but also intrinsically through transient liquid properties and characteristics.^30^ The development and optimization of this novel class of fluorine-free solutions and the corresponding nanoscale homogeneity of the precursor films are seen as a requirement for the ultrafast TLAG of high-performance thick films and CCs.

CSD is a facile, low-cost method used for the preparation of various functional materials,^19^ including REBCO (RE = rare earth elements, Y). Here, we applied CSD methodology to the preparation of nanocrystalline precursor layers for superconducting YBCO films. In order to obtain thick, reproducible, and high-performance YBCO superconducting films with ultrafast growth rates, a novel class of metal–organic fluorine-free solutions had to be developed for the use in the previously pioneered TLAG-CSD process. In this article, we describe the study that led to achieving adequate optimized solutions for the preparation of thick YBCO superconducting films. The progress leading to the final optimized novel class of solutions implied a thorough study of solution rheology, which unraveled the unique characteristics of this system. Moreover, the optimized solutions yielded nanocrystalline precursor layers from the first low-temperature annealing, the pyrolysis process. A detailed microstructural study was key to identify the correct morphology and microstructure needed for the successful TLAG of high-performance epitaxial YBCO. Additionally, it led to the understanding of various phenomena that take place during the pyrolysis process of the different solutions tested. The process of optimization underwent various stages, all of which will be described in detail: first, the solution formulation, referring to the solution design and preparation procedure, is determined; later, the microstructural analyses of nanocrystalline layers yielded valuable information on the characteristics of the precursor films, closely linked to the rheological and chemical analysis of solutions, which enabled us to fully understand the solution system. Finally, the growth from optimized precursor films, which distinctly demonstrates how fundamental the solution properties and microstructural characteristics of the nanocrystalline precursor layers are to the final physical properties of the YBCO superconducting film.

## Experimental Section

### Synthesis of Metal-Propionate Precursors

To synthesize copper propionate (Cu(Prop)_2_) and yttrium propionate (Y(Prop)_3_), CuO [copper(II) oxide, Puratronic, 99.7% (metals basis), Alfa Aesar] and Y_2_O_3_ [yttrium(III) oxide, REacton, 99.99% (REO), Alfa Aesar] are separately added to an excess of propionic acid (HProp) (≥99.5%, Sigma-Aldrich). The reagent and the solvent are used without any further purification. The reactions were conducted overnight at 140 °C under vigorous stirring until a clear, blue solution for Cu(Prop)_2_ and a transparent, clear solution for Y(Prop)_3_ was achieved. In the case of Cu(Prop)_2_, excess solvent was eliminated using a rotary evaporator (Büchi) to obtain a dry, dark-blue solid. In the case of Y(Prop)_3_, precipitation of a white solid is acquired upon cooling.

For the synthesis of barium propionate (Ba(Prop)_2_), BaCO_3_ [barium (II) carbonate, 99.95% (metals basis), Alfa Aesar] is added to a mixture of HProp and distilled water. The reagent and solvents are used without further purification. The reaction starts as a highly foamy, white solution, and the reaction mixture is stirred vigorously during 24 h. Excess solvent was eliminated using a rotary evaporator (Büchi) to obtain a transparent gel. To induce crystallization of the solid product from the gel, the product is placed in a bath of ice and acetone.

Finally, all three solid products were washed with diethyl ether (Et_2_O, AGR, ACS, ISO, stabilized with BHT, Labbox) using a Büchner Funnel, a necessary step to eliminate any residual HProp, resulting in high-purity powder products (see Supporting Information Section SI, Figure S1); through this synthetic method, we ensure yields of above 90%.

The grain sizes of final powder products of Y(Prop)_3_ and Ba(Prop)_2_ after manual crushing are ∼10 and ∼6 μm, respectively. However, in the case of Cu(Prop)_2_, the final powder product after manual crushing presented a large grain size, with the majority of grains over 100 μm; moreover, the grains showed a crystal-like shape, hindering its complete solubility in the following YBCO precursor solution for high concentrations. Therefore, the product was grinded using a Fritsch Pulverisette 6 mono-planetary ball mill to obtain grains of 30 μm or lower (Figure S2).

The final products are characterized through attenuated total reflectance (ATR)-Fourier transform infrared ( FTIR) spectroscopy (Spectrophotometer Jasco 4700, Energy range: 300–7800 cm^–1^, equipped with ATR accessory) (Figures S1 and S3), X-ray diffraction (XRD) (Siemens D-5000) (Figure S4), nuclear magnetic resonance (NMR) spectrometry [Bruker Advance DPX, 250 MHz (5.8 T), characterization restricted to Ba(Prop)_2_ and Y(Prop)_3_, due to the paramagnetic nature of Cu(II) in Cu(Prop)_2_] (Figures S5 and S6), scanning electron microscopy (SEM) (QUANTA FEI 200 FEG-ESEM) for the evaluation of grain size (Figure S2), and thermogravimetric analysis (TGA) coupled to FTIR using a Mettler-Toledo thermobalance (model TGA/DSC1) (Figure S7).

### YBCO Precursor Solution Preparation

The preparation of YBCO precursor solutions follows the same procedure independent of the stoichiometries of the solution in question. The various stoichiometries of solutions differ in the Y–Ba–Cu ratio and are named considering the Ba–Cu molar ratio of the transient liquid formed during the TLAG process as follows: the YBCO-stoichiometric mixture with a Y–Ba–Cu proportion of 1:2:3 [(2:3) composition], a Cu-rich mixture with proportion of Y–Ba–Cu of 1:2:4.66 [(3:7) composition], and excess of Cu-rich composition corresponding to a proportion of Y–Ba–Cu of 1:2:5.5 [(4:11) composition]. These solutions were prepared using a mixture in the ratio (50:50) of HProp and methanol (MeOH) [methanol, 99.9%, anhydrous (max. 0.003% H_2_O), Scharlab] as solvents and the addition of different %_v/v_ of monoethanolamine (MEA) (purified by redistillation, ≥99.5%, Sigma-Aldrich). The concentration of YBCO solutions for spin-coating deposition was 1.75 M in sum of metal salts when MEA is used, independent of the solution composition. MEA aids complete precursor dissolution and increases the stability of solutions and the homogeneity and thickness of the final precursor films. If no amine additive is used, only a maximum total concentration of 1 M in sum of salts is possible.

Metal-propionate precursors were added to the mixture of solvents in consecutive order, allowing for the complete dissolution of each precursor in the solution before the addition of the following one (see the Supporting Information, Section SII, for the solution preparation procedure).

Solutions’ rheological properties such as viscosity and contact angle were measured with a HAAKE RheoStress RS600 from Thermo Electron Corp and Drop Shape Analyzer DSA 100 from Krüss, respectively (Table S1). The water content of the solutions is crucial as it may influence the final properties of the REBCO layers; it is thus monitored through the Karl–Fischer titration method (Nittoseiko Analytech, Model CA-310 equipped with a VA-200 vaporizer),^31^ and each solution is only used until % H_2_O < 2 wt %. Electron paramagnetic resonance (Bruker ELEXYS E500 X band EPR spectrometer) measurements were carried out on certain solutions to examine the role of MEA.

### Thin-Film Deposition, Pyrolysis, and Growth

As-prepared YBCO precursor solutions with different compositions were deposited via spin coating (SMA 6000 Pro, Suministro de Materiales y Asistencia, S.L.) in a grade ISO7 clean room using spin coating at 10% humidity at a spinning rate of 6000 rpm for 2 min on single-crystal (001) SrTiO_3_ (STO) substrates (CrysTech GmbH). Before deposition, substrates undergo an annealing process at 900 °C for 5 h to obtain flat-terraced surfaces, successively cleaned with acetone (acetone, Multisolvent HPLC grade ACS ISO UV-VIS, Scharlab) and methanol (methanol, Multisolvent HPLC grade ACS ISO UV-VIS K.F., Scharlab) to eliminate any possible residues.

The pyrolysis process was done by heating in humid oxygen flow (0.12 L/min) up to 500 °C at rates of 3–5 °C/min, followed by cooling to room temperature. To obtain thicker precursor films, multideposition processes were carried out repeating the above procedure.

Finally, the growth of the final epitaxial YBCO layers through the innovative process of TLAG on the multilayered precursor films has been performed using a tubular furnace connected to a vacuum system as described in the Supporting Information.

### Sample Characterization

Characterization of nanocrystalline precursor samples was performed by optical microscopy (OM) (Leica DM1750 M) analysis to inspect homogeneity of the films as well as reflectometry measurements (Filmetrics F50) that allow us to know the thickness of the film through a rapid and non-destructive technique.

Moreover, the decomposition process of these solutions has been studied through TGA coupled to FT-IR using a Mettler-Toledo thermobalance (model TGA/DSC1) to fully understand gas evolution during the heat treatment, which inevitably leads to the formation of specific phases in the final precursor layers. During this analysis, samples were heated at a constant rate of 5 °C/min under a dynamic atmosphere, created through a carrier gas with a flow rate of 55 mL/min and a protective gas with a flow rate of 15 mL/min. Humid oxygen flow used in a pyrolysis process was recreated by bubbling the carrier gas in a distilled water flask at standard temperature and pressure (25 °C, 1 atm).

### Structural Characterization

XRD on a Bruker-AXS D8 Advance diffractometer [Cu Kα equipped with a general area detector diffraction system (GADDS)] was used to characterize the structure and phase composition of the as-prepared precursor and grown YBCO layers. PDF cards used for XRD peak identification were Y_2_O_3_ (00-041-1105), CuO (00-048-1548), Cu_2_O (01-078-2076), Cu (00-004-0836), BaCO_3_ orthorhombic (00-005-0378), BaCO_3_ monoclinic (01-078-2057), and YBCO orthorhombic (04-006-6962).

Additionally, for YBCO films, a Bruker D8 Discover system (Cu Kα, X-ray energy = 8.049 keV) equipped with a Lynxeye XE-T energy-dispersive one-dimensional (1D) detector measuring in two configurations was also employed: θ–2θ geometry to evaluate epitaxy and grazing incidence (GI) geometry to amplify signals coming from secondary phases, facilitating their identification.

### Microstructural Characterization

The surface morphology and composition were studied using SEM and energy-dispersive X-ray (EDX) spectroscopy, respectively, with a QUANTA FEI 200 FEG-ESEM.

For thickness evaluation and a deeper understanding of the distribution and sizes of nanocrystalline phases, the microstructure of precursor thin films was studied by means of high-resolution transmission electron microscopy (TEM) (HRTEM), high-angle annular dark-field scanning transmission electron microscopy (HAADF-STEM), EDX spectroscopy, and electron energy loss spectroscopy (EELS). For this purpose, an FEI Tecnai F20 (S)TEM operated in both TEM and STEM modes at 200 kV, equipped with a Gatan quantum electron energy-loss spectrometer for EELS analyses, was used.

Pore density was evaluated from the cross-sectional STEM-HAADF micrographs, processed with the image analysis software ImageJ,^32^ where well-defined dark contrast areas were defined as pores using the threshold tool and analyzed with the “analyze particle function”. The average pore sizes were calculated from the histograms of measured pore areas considering round pores.

For EELS data processing, principal component analysis (PCA) was used in order to reduce the statistical noise in EELS spectrum images. A reconstruction using the first 10 principal components was performed using the weighted-PCA multivariate statistical analysis (MSA) Plugin^33^ within Gatan Digital Micrograph software.

Moreover, the microstructure, atomic-defect structure, and phase composition of grown YBCO thin films were studied using the FEI Tecnai F20 (S)TEM operated at 200 kV as well as an FEI Titan with an X-FEG gun, a CESCOR Cs-probe corrector, and a Gatan TRIDIEM 866 ERS energy filter with a monochromator, operated in the STEM mode at 300 kV.

For TEM studies, cross-sectional specimens were prepared by conventional methods: cutting, gluing the slices face-to-face, and thinning down by tripod mechanical polishing, followed by Ar^+^ ion milling using Gatan PIPS, until electron transparency was attained.

### Electrical Characterization

The critical temperature (*T*_*c*_) is calculated from electrical resistivity measurements in Van der Pauw configuration using a quantum design physical property measurement system (PPMS). The magnetic field dependence of the transport critical current density (*J*_c_) up to 9 T was also determined from the *I*(*V*) characteristics measured in the same PPMS system, with a 10 μV/cm criterium in bridges of a width of tens of μm and a length around 200 μm. A maximum Lorentz force configuration was applied between the applied magnetic field and the transport current.

## Results and Discussion

### Novel Solution Formulation

The fundamental requisite for the scale-up and industrial appeal of these materials is the preparation of thick YBCO films with high performance.^34−36^ For this purpose, one of the crucial requirements in CSD is the need to develop a robust solution synthesis method compatible with spin coating and industrially scalable processes (slot die, inkjet printing) with the possibility to tune the concentration and rheology without compromising reproducibility, solution stability, and homogeneity, being the final thickness of the films directly correlated to solution rheology and the deposition method.^23,37−39^

Several studies showed the use of acetates of yttrium, barium, and copper as precursors for the preparation of fluorine-free YBCO precursor solutions through dissolution in HProp-based media.^40−47^ However, complete conversion of acetates into propionates is never assured; specifically, in the case of barium acetate, even in conditions of excess of HProp, only a mixed complex of Ba-Prop-Ac can be obtained.^48^ The synthesis of propionates of yttrium, barium, and copper was found to be imperative to avoid the presence of product mixtures in solution that may endure different decomposition paths, hindering an optimal and reproducible result of the final precursor films.

We developed a cost-effective, robust, and reproducible process to prepare the three metal-propionate salts of high purity through facile one-pot syntheses, without hazard media that may hamper the successive preparation of high-concentration YBCO precursor solutions. The full preparation procedure and characterization of the propionate salts are detailed in the Experimental Section and Supporting Information, Section SI.

The precursors of choice for the metal propionates were CuO, Y_2_O_3_, and BaCO_3_, given their low cost and high purity commercially available. Indeed, when compared to the respective acetate salts, a large cost-wise difference is observed, with the most notable being the case of copper precursors where a cost decrease of at least a factor 10 is achieved.

The initial step in the development of the novel fluorine-free solution for YBCO films was the preparation of a saturated solution employing the newly synthesized metal-propionate precursors. It must be taken into account that the first optimization effort was focused on the (3:7) composition, then successively adapted to the (2:3) and (4:11) compositions. We will concentrate on the case of (3:7) composition solutions, if not specified differently, as previous studies in our group showed this stoichiometry to enhance epitaxial growth of YBCO in TLAG.^23,49^ Further details regarding solution composition nomenclature are available in the Experimental Section.

Simple addition of the three metal-propionate salts in a mixture of propionic acid and methanol (50:50) yielded a solution of 1 M in sum of salts as the maximum concentration (the full procedure is described in the Experimental Section), given the limited solubility of Cu(Prop)_2_ in these media. Despite being stable during several months and allowing us to prepare homogeneous, crack-free precursor films, this solution resulted in layers of merely 100 nm thickness (Figure S8a). Therefore, the development of a specific protocol for the formulation and synthesis of a high-concentration solution incorporating an additive was considered. Nevertheless, our system required a careful selection of the additive to avoid its interference in the correct formation of the nanocrystalline precursor layer. Typically, the use of the additive should aid in obtaining homogeneous precursor layers of low porosity, which additionally should be suitable for multideposition.

Thus, the chemical requirements for the additive choice were as follows: first, it must aid in increasing Cu(Prop)_2_ solubility in the media used, by the formation of a complex with Cu(Prop)_2_. For this reason, a possible candidate was a compound from the amine family due to the renowned capability and easiness of Cu–N complex formation.^50^ Second, the chemical formula of the compound chosen was fundamental as a high C number creates an excess of C in the film, which makes it more prone to form the undesired monoclinic BaCO_3_ phase, observed to be formed when insufficient CO_2_ elimination during the pyrolysis occurs, as will be shown subsequently. Additionally, BaCO_3_ monoclinic phase was demonstrated to delay the BaCO_3_ decomposition in the successive growth process.^38,51^ Therefore, a short C-chain should be ideal. Third, it must decompose during the pyrolysis process and constitute part of the intricate metal–organic skeleton in a way to properly release the strain to avoid cracking.^38,39,52^ Consequently, considering all restrictions, the best amine additive selected was MEA. It fulfilled all the previous conditions, and furthermore, being an aminoalcohol, it contains an −OH functional group together with an −NH_2_ functional group, bearing possible stabilization through H-bonds, apart from feasibility for complexation.

The final optimization of the novel propionate-based solution preparation protocol is described in the Supporting Information, Section SII. We successfully reached a concentration of 1.75 M (sum of metals concentration) with 4%_v/v_ MEA for a (3:7) composition in a solvent mixture of 50:50 HProp/MeOH, with this solvent mixture being a compromise for solubility and layer thickness. This solution was found to yield single layers of high thickness (as shown in Figure S8) and to be suitable for multideposition, as will be demonstrated in the next section. Additionally, it was also adapted to scalable deposition techniques such as inkjet printing and slot die coating. However, it is relevant to discuss several fundamental aspects that were encountered during solution optimization.

**Figure 1:**
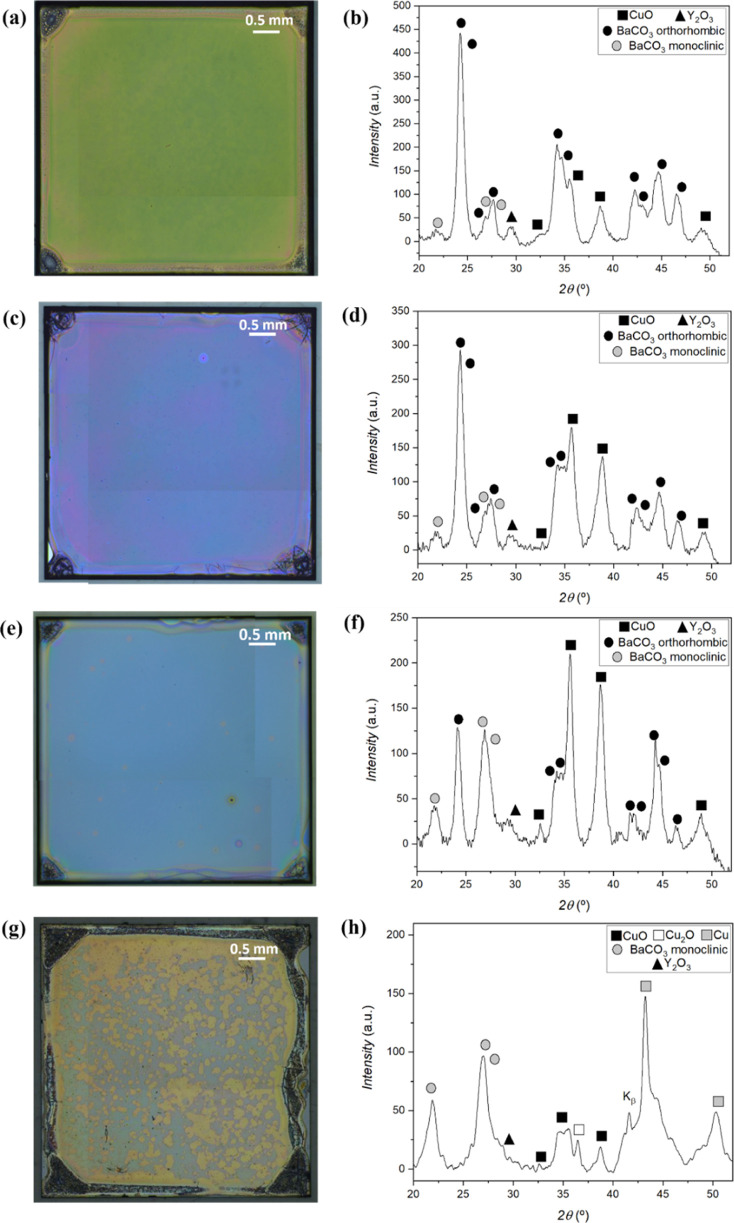
OM images and XRD patterns of nanocrystalline precursor samples with two layers of (a,b) (2:3) composition using 1.75 M + 4.3%_v/v_ MEA solution, (c,d) (3:7) composition using 1.75 M + 4%_v/v_ MEA solution, and (e,f) (4:11) composition using 1.75 M + 4%_v/v_ MEA solution and one layer of (g,h) (3:7) composition using excess MEA, in this case, 1.75 M + 8%_v/v_ MEA solution, respectively. The thickness for two layers is in the range of 700–850 nm for all the three compositions.

The XRD analyses of layers prepared by using this solution, shown in Figure 1a–f, exhibit the desired precursor phases for TLAG:BaCO_3_ (mainly in its orthorhombic phase), CuO, and Y_2_O_3_, confirming the promising prospect of this class of solutions for the growth of thick and robust YBCO films.

From Figure 1, it is evident that this solution can provide smooth, crack-free films for all three compositions, that is, (2:3), (3:7), and (4:11), with their respective optimum quantity of MEA. Figure 1g,h demonstrates that for (3:7) composition, when an excessive amount of MEA is added (here, 8%_v/v_ MEA), we observe morphologic irregularities of the film surface, and XRD analysis displays the formation of undesired phases: BaCO_3_ is found only in its monoclinic phase and copper is majorly present as metallic Cu (Cu(0)). These phases are detrimental for YBCO nucleation and growth as BaCO_3_ monoclinic has a slower decomposition pathway than its orthorhombic phase;^53^ additionally, metallic Cu is subject to strong coarsening phenomena and, in our system, it is seldom reoxidized to CuO,^51^ thus hindering the homogeneous formation of the transient liquid of Ba–Cu–O and the subsequent TLAG process. The optimization for the (2:3) composition is shown in Figure S9e–i, where it is also interesting to confirm how a mere 0.2%_v/v_ MEA difference from the optimum quantity can greatly alter the morphology of the sample. Hence, it is worth stressing how crucial the correct amount of additive is to our final objective not only for morphological reasons but also for the microstructural perspective: the addition of high quantities of MEA on one side undoubtedly favors dissolution of the precursor salts in the solution, but this must contemporarily give rise to high-quality precursor films with the desired nanoscale homogeneity required for the growth of TLAG films of high performance. Thus, a compromise between solution preparation and the final result of the pyrolysis process needs to be found.

**Figure 2:**
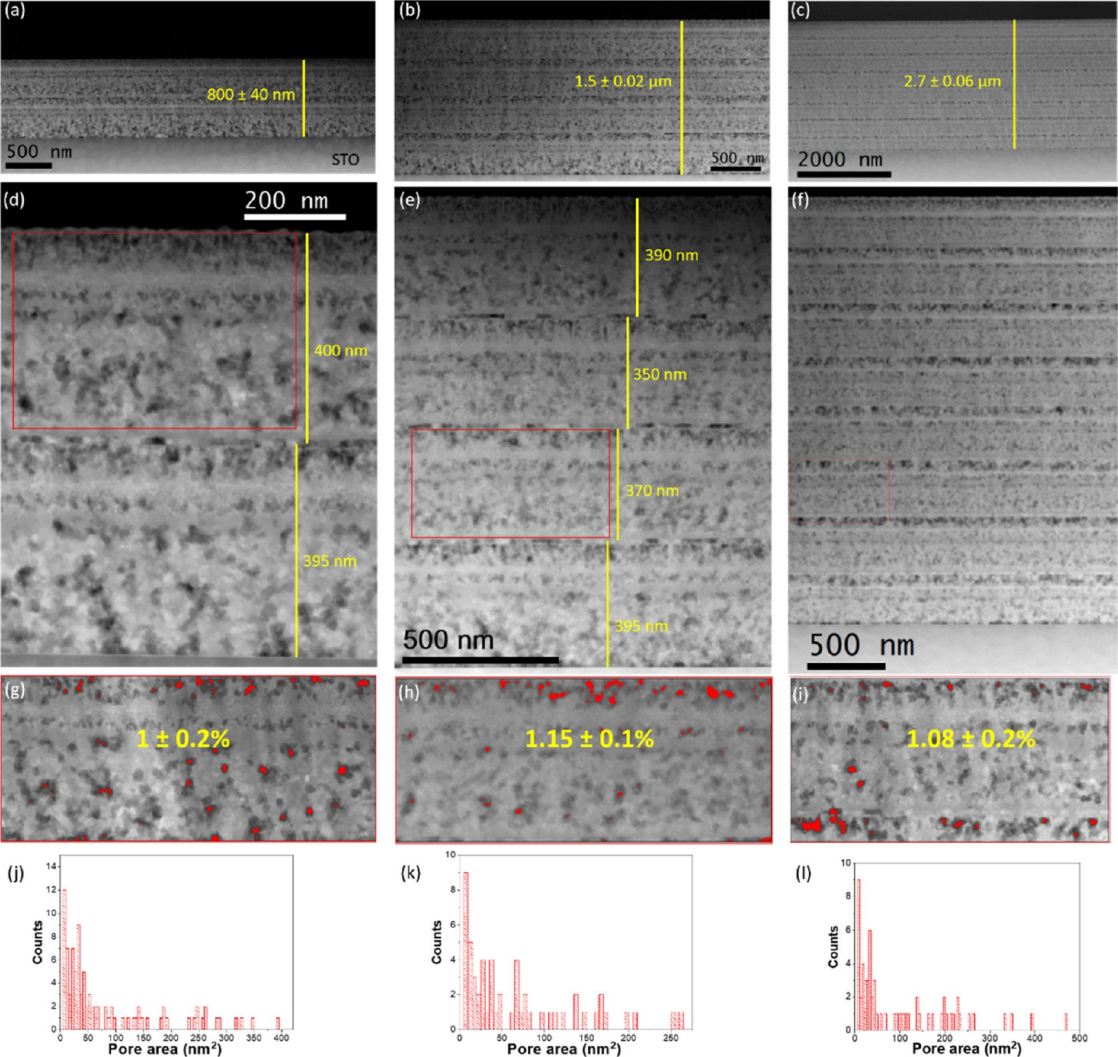
Cross-sectional low-magnification HAADF-STEM images of (3:7) precursor thin films deposited using 1.75 M + 4%_v/v_ MEA solution with (a) two layers, (b) four layers, and (c) eight layers. Magnified STEM-HAADF images displaying similar thicknesses of individual layers for (d) two layers, (e) four layers, and (f) eight layers. (g–i) Pore density analysis using ImageJ from the red rectangular regions in (d–f), respectively, where pores are colored in red for quantification. (j–l) Histograms of pore areas, used to calculate the average pore sizes.

Consequently, upon optimization, successful, reproducible, fully dissolved, and stable solutions could be achieved for several Y/Ba/Cu compositions, which yield smooth, high-quality, crack-free, thick precursor layers (400 nm for one single layer), with desired nanocrystalline precursor phases. We also achieved thicker films using multilayered samples deposited by spin coating, as shown in Figure 2; moreover, all films present the microstructural characteristics needed for the TLAG process, as will be further analyzed in the next section. Additionally, this solution was also found to be compatible with inkjet printing and multilayered depositions by slot die coating on metallic substrates.

### Microstructural Analysis of Nanocrystalline Layers

**Figure 3:**
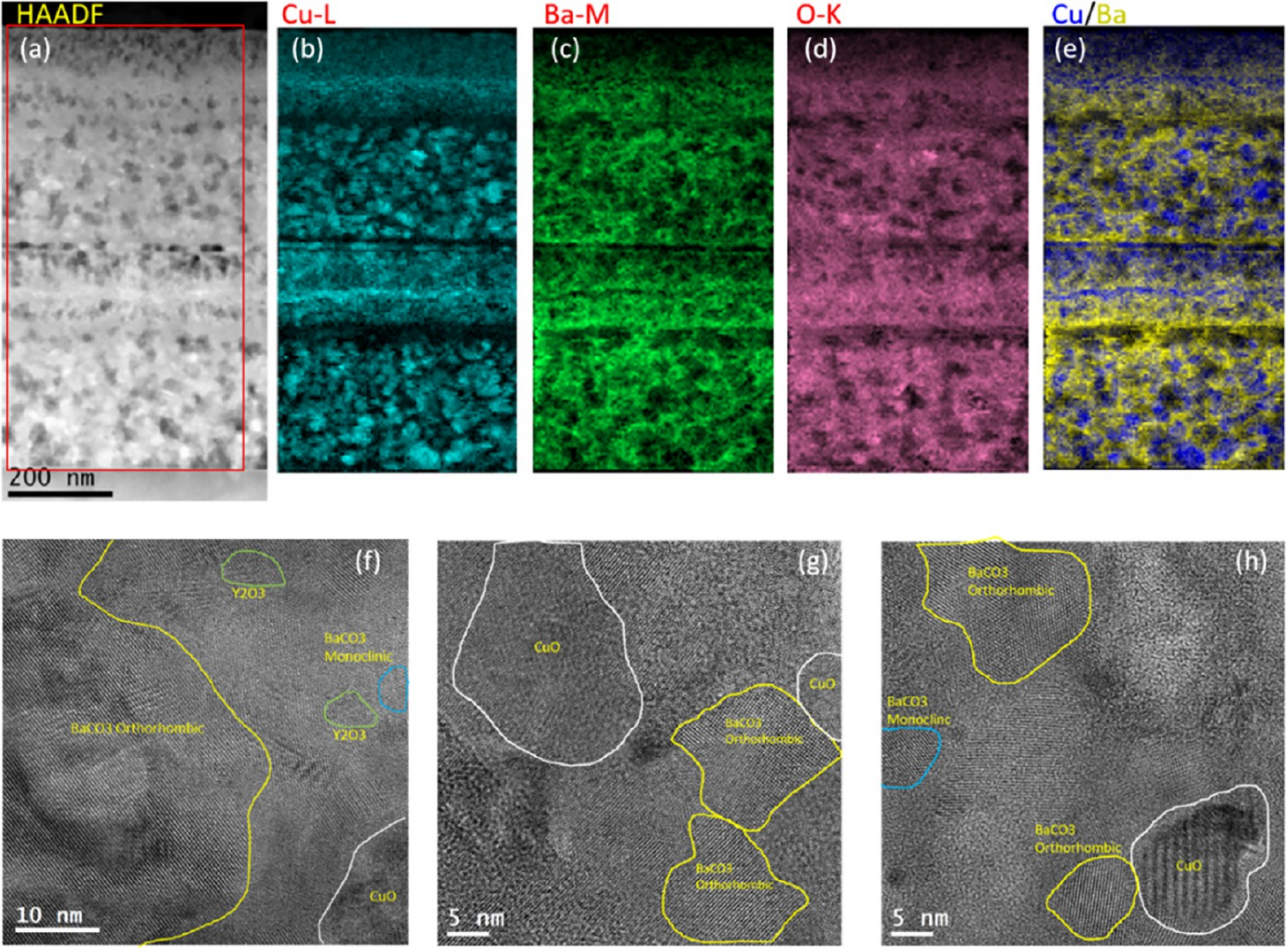
(a) HAADF-STEM image of a two-layer (3:7) precursor film deposited using 1.75 M + 4%_v/v_ MEA solution. Elemental EELS maps of (b) Cu-L edge, (c) Ba-M edge, and (d) O-K edge; (e) composite EELS map of Cu (blue) and Ba (yellow) from the red rectangular region in (a). (f–h) HRTEM images collected from different regions of the precursor film, displaying the individual sizes and the presence of all precursor phases in the same area, which exhibit a homogeneous distribution.

Microstructural investigation of the precursor films by various techniques of electron microscopy shows that the films of three Y/Ba/Cu compositions with optimized MEA quantities have similar characteristics: smooth surfaces, homogeneous distribution of desired nanocrystalline phases, and high thicknesses of the multideposition layers [Figures 2 and 3 for (3:7) composition and Figures S10 and S11 for (2:3) and (4:11) compositions, respectively]. As shown in Figure 2, multideposition did not affect in any way the final quality and microstructure integrity of the multipyrolyzed sample, and the thickness of each individual layer was maintained. The cross-sectional HAADF-STEM images display that samples with two layers result in 800 ± 40 nm thick films, samples of four layers reach 1.5 ± 0.02 μm in thickness, and most remarkably, samples of eight layers yield a final thickness of 2.7 ± 0.06 μm, implying no loss of homogeneity or differences in the nanocrystalline matrix (Figure 2), evidencing that the limit is yet to be reached.

In detail, the low-magnification HAADF-STEM images exhibit the smooth surfaces for all three (3:7) precursor thin films along with their respective thicknesses (Figure 2a–c). Moreover, Figure 2d–f reveals that CuO nanocrystals are homogeneously distributed within a BaCO_3_ matrix, with no segregations or interfaces, an ideal situation for a successful epitaxial growth of YBCO. In fact, segregated CuO interlayers at the interfaces of different layers in multiple depositions with intermediate pyrolysis are a common downside, especially widespread in the TFA route.^38^ In Figure 2g–l, through the HAADF-STEM image analysis using the software ImageJ, estimated pore densities as low as 1 ± 0.2, 1.15 ± 0.1, and 1.08 ± 0.2% are determined for precursor films with two, four, and eight layers, respectively. These low-porosity precursor films benefit from a fast BaCO_3_ and CuO reaction to the transient liquid but could probably be at the limit where gas transport (out-diffusion of CO_2_/CO and diffusion of O_2_) is hindered during the pyrolysis to ensure the nanocrystalline phase mixture desirable, as discussed below.

Further elemental analysis on these precursor films is performed through EELS, where elemental maps of CuL_2,3_, BaM_4,5_, and OK reveal high homogeneity of these precursor phases at the nanoscale (Figure 3a–e). A complementary compositional analysis is conducted availing EDX spectroscopy (Figure S12), and Y, Ba, and Cu STEM–EDX cross-sectional elemental maps also confirm the uniform distribution of nanocrystalline phases. The spatial distribution, crystalline state, and sizes of precursor phases could be determined through the analysis of HRTEM images. All three nanocrystalline phases, that is, BaCO_3_, CuO, and Y_2_O_3_, are distinctly identified in multiple HRTEM images corroborating a robust solution composed of these precursor intermediates, with typical diameters of 10–30 nm for orthorhombic BaCO_3_, 5–7 nm for monoclinic BaCO_3_, and 10–25 nm for CuO, and the diameters of Y_2_O_3_ remain as small as 5–6 nm (Figure 3f–h). With TLAG being an ultrafast liquid-assisted process, the small size and homogeneous distribution of the nanocrystalline precursors greatly favor homogeneous and fast liquid formation by easing atomic mobility, promoting high epitaxial layers at ultrafast growth rates. Likewise, as the majority of BaCO_3_ is present in the orthorhombic phase, it is advantageous due to its straightforward reaction with nanocrystalline CuO in the following TLAG process.

**Figure 4:**
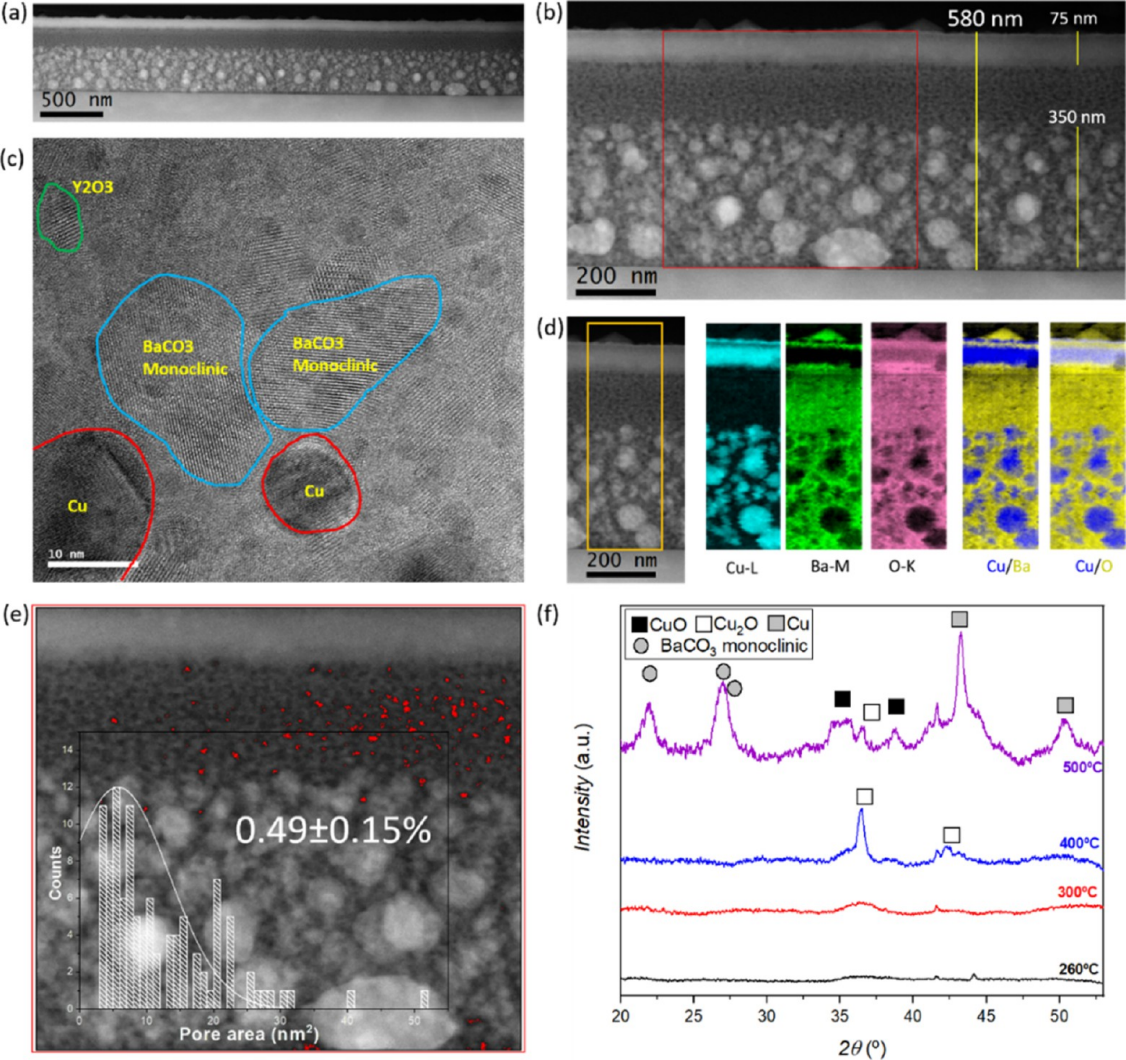
(a) Cross-sectional low-magnification HAADF-STEM image of a single-layer (3:7) precursor thin film with a solution of 1.75 M + 8%_v/v_ MEA (excess of MEA). (b) Magnified HAADF-STEM image demonstrating the thickness of the layer to be 580 ± 40 nm. (c) HRTEM image displaying the presence of metallic Cu and monoclinic BaCO_3_. (d) Elemental EELS maps of Cu-L edge, Ba-M edge, and O-K edge and composite EELS map of Cu (blue)–Ba (yellow) and Cu (blue)–O (yellow), confirming the metallic Cu NPs. (e) Pore density analysis using ImageJ from the red rectangular region in (b), where pores are colored in red for quantification. Histograms of pore area are used to calculate the average pore sizes. (f) XRD patterns of MEA excess samples which underwent pyrolysis processes at different temperatures.

Besides, from the TEM analysis of the precursor film prepared with a solution 1.75 M + 8%_v/v_ MEA (excess of MEA) (Figure 4), it is evident how adverse an excess of MEA is to our purpose. In spite of the thickness being higher than in the case of a sample with optimized MEA quantity (∼200 nm more), the microstructure differs completely, as shown in the HAADF-STEM images in Figure 4a,b. HRTEM confirms the presence of metallic Cu, along with monoclinic BaCO_3_ in majority (Figure 4c), as previously observed in the use of acetate-based precursor solutions.^54^ In fact, the varying sizes of metallic Cu between 10 and 100 nm result in enlarged inhomogeneity of each precursor phase in the film. The elemental EELS maps (in Figure 4d) reveal that the phases are segregated in specific areas of the precursor layer, with large grains of metallic Cu in the lower part, followed by an area of mainly BaCO_3_ monoclinic and superficial individual dense layers of CuO and BaCO_3_ on top. Through the HAADF-STEM image analysis using ImageJ, a pore density of mere 0.49 ± 0.15% is estimated (Figure 4e), where the pores are inhomogeneously distributed in the film and the segregated CuO top layer contains almost no pores. Figure 4f shows XRD analyses of MEA excess sample precursor at different temperatures for a thorough understanding of the phase formation during the pyrolysis process. It is clear that until the final part of the process, there is an amorphous nature of the film, with no distinction in crystalline phases relative to copper, indicating that the reduction of copper species does not coincide with the decomposition of Cu(Prop)_2_, which should be initiated after solvent evaporation, as early as 150 °C.^54^ At 400 °C, the crystallization of Cu_2_O takes place, and at the final temperature of 500 °C, corresponding to the complete decomposition of all the species involved, the intense peaks of BaCO_3_ monoclinic are the only ones deriving from the complete decomposition of Ba(Prop)_2_. However, the species relative to copper are now CuO and, in majority, metallic Cu. Therefore, the reduction of Cu_2_O to metallic Cu occurs at the final stage of the pyrolysis process. It is in our opinion that the higher thickness and extremely low porosity of the film (Figure 4) severely diminished the O_2_/CO_2_/CO transport, thus impeding CO_2_ elimination from the internal part of the layer: with the CO_2_ and CO release from the decomposition of Ba(Prop)_2_,^54^ the strongly reducing CO media locally created would be responsible for the reduction of CuO to Cu_2_O and finally metallic Cu through the reaction CuO + CO → Cu + CO_2_. The latter redox reaction seems to coincide with the temperature range corresponding to Ba(Prop)_2_ decomposition, when the highest amount of CO_2_ and CO (due to decomposition of intermediate barium oxalate^55,56^) should be released inside the film, confirming our hypothesis. This is also in agreement with the fact that CuO is only found in the surface area of the film (Figure 4d), most exposed to the oxygen flux, and the most reduced phases of Cu are segregated in its lowest part (from where there is CO_2_ out-diffusion or CO_2_ or O_2_ diffusion into the film): considering Fick’s law of diffusion^57^ when the thickness of a film is increased, the ability of CO_2_ to reach the surface of the layer and thus be expelled decreases importantly. In the specific case of MEA excess, the extremely low porosity additionally complicates CO_2_ expulsion from the film, thus contributing to the experimental result obtained.

**Figure 5:**
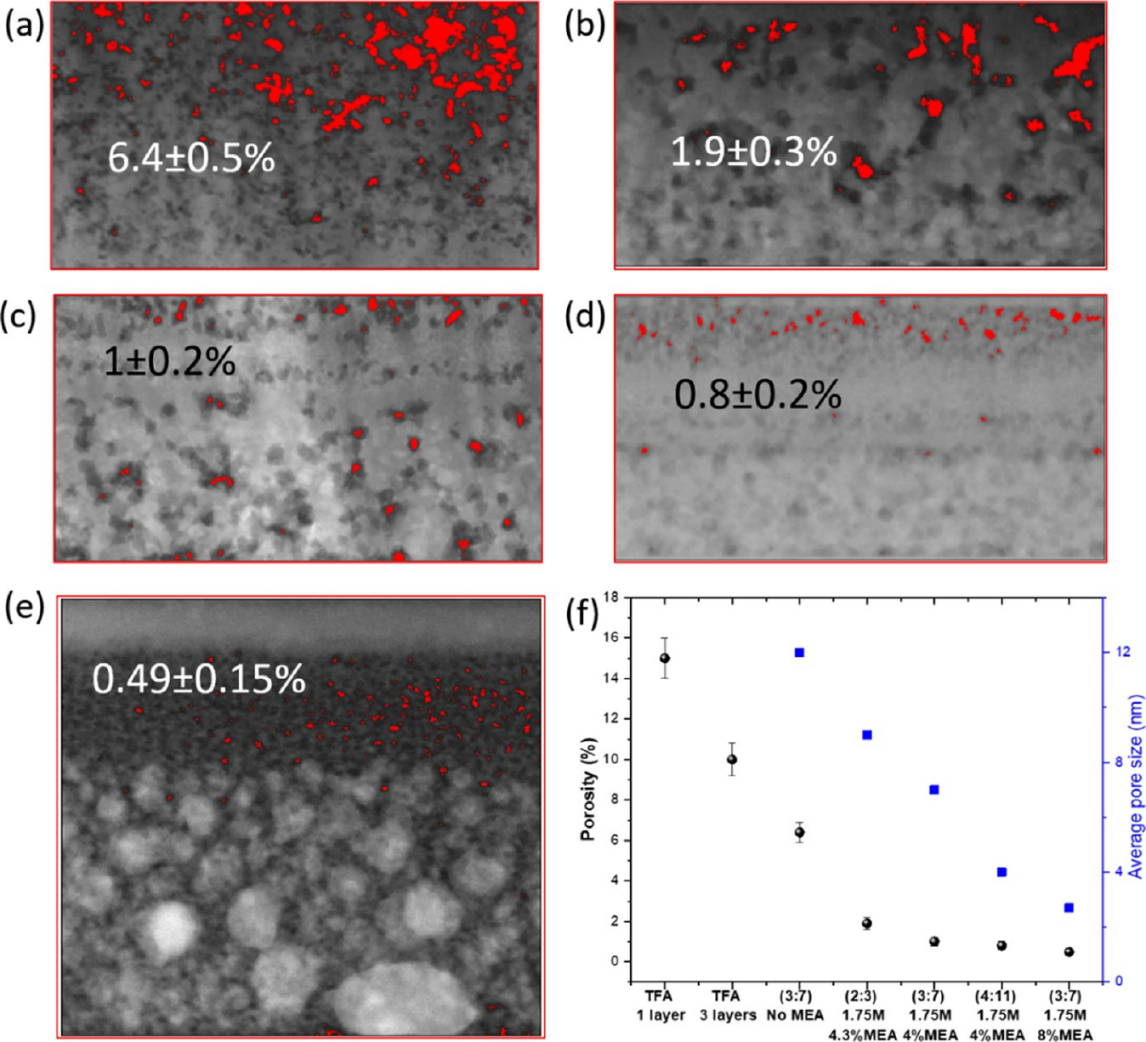
Estimation of pore density using image analysis of HAADF-STEM via ImageJ for (a) no MEA (3:7), (b) 1.75 M + 4.3%_v/v_ MEA (2:3), (c) 1.75 M + 4%_v/v_ MEA (3:7), (d) 1.75 M + 4%_v/v_ MEA (4:11), and (e) 1.75 M + 8%_v/v_ MEA (3:7), where pores are colored in red for quantification. (f) Plot of porosity and average pore sizes for all samples.

Through the HAADF-STEM image analysis using ImageJ, an estimation of the pore density can be performed and average pore sizes can be calculated using the histograms mentioned in Figures 2j–l, 4e, and S10d and S11e. A comparison of pore density analysis for each sample is shown in Figure 5, which implies a significant reduction upon addition of MEA for the same composition (3:7), and it also decreases with the increase of the amount of copper in the composition (Figure 5a–d). A similar trend can be found for the average pore sizes as well. Moreover, increasing the amount of MEA above the optimum value is followed by an inhomogeneous pore distribution, displaying almost no pores in the top CuO segregated layer and an extremely low degree of porosity in the lower part of the film, along with small average sizes (Figure 5e,f). The significant reduction in porosity and smaller sizes of pores in the sample with excess of MEA are seen to hinder the O_2_/CO_2_/CO gas transport during pyrolysis, resulting in metallic Cu and monoclinic BaCO_3_ phases (Figure 4f). Nonetheless, the low degree of porosity (in the range of ∼1–2%) for the optimized samples is expected to be of great advantage for the epitaxial growth of YBCO through TLAG, suggesting once again the beneficial nature of MEA. Notice the high porosity of layers without MEA added in the solution, and for the extreme case, samples prepared through the TFA route^38^ (Figures 5f, S13), a rather reduced porosity could be reached with the novel propionate-based solution developed, ensuring a homogeneous nanocrystalline mixture of CuO, Y_2_O_3_, and mainly orthorhombic BaCO_3_ phases in the precursor films.

### Rheological and Chemical Analysis of the Precursor Solutions

**Figure 6:**
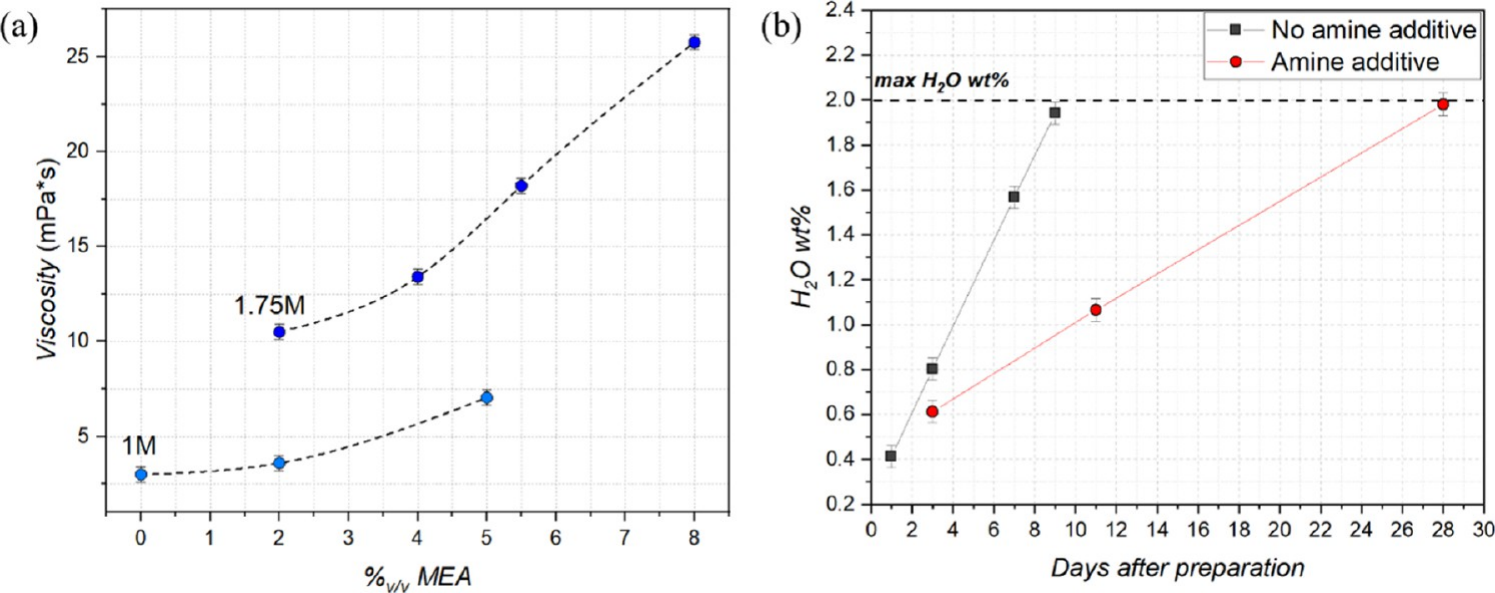
(a) Viscosity measurements with varying quantities of MEA for (3:7) solutions of 1 M and 1.75 M concentrations. (b) Evolution of H_2_O wt % over time for (3:7) solutions with and without MEA, measured using the Karl–Fischer titration method.

At this stage, we want to disentangle several aspects of this novel solution that provide its beneficial physico-chemical characteristics, mainly associated directly or indirectly to the role of MEA. Figure 6a displays a series of viscosity measurements conducted on solutions of two concentrations, 1 M and 1.75 M, in sum of metals. It is evident that an increase in MEA content strongly increases the viscosity of the solution in both cases. As the concentration and viscosity of the solutions are directly correlated with the resulting precursor film thickness, the addition of MEA to our system is definitely of great benefit to the purpose of increasing the final thickness. However, as shown in the previous sections, only a well-defined value of MEA worked to our advantage, forcing us to find a compromise between solution rheology and precursor film quality.

Previous studies of CSD have shown that YBCO film quality is strongly affected by the water content in the solutions beyond a certain threshold,^58^ usually established to be approximately 1.5% wt for full fluorine and 2% wt for low fluorine or fluorine-free solutions, resulting in continuous monitoring of the solution through the Karl–Fischer titration method.^59^ As this class of solutions uses a mixture of HProp and MeOH (50:50) as solvents, they are inevitably subject to Fischer esterification,^60,61^ producing H_2_O as a by-product of this reaction. Figure 6b shows the H_2_O wt % evolution of two (3:7) composition solutions over time, one of which contains MEA in optimized quantity. The clear difference in slope of the H_2_O wt % trends can be ascribed to the presence of the amine additive. When an amine is added to an acid, the deprotonation of the acid occurs, forming a carboxylate, contemporarily to the formation of the protonated amine. As Fischer esterification sees its first step in the protonation of the carbonyl group of the acid, fundamental to activate its C for the successive nucleophilic attack by the alcohol to form the tetrahedral intermediate, the formation of a carboxylate deters this protonation and thus the whole reaction to occur (reaction mechanism shown in Figure S14). Evidently, as the quantity of acid in solution is in enormous excess with respect to the quantity of amine, the reaction is only slowed down and not completely hampered. Still, the addition of 4%_v/v_ of MEA in the solution increases by a factor 3 the time to reach the criterion of 2 wt %. It is important to notice that a dedicated study of the effect of water content in this novel solution to demonstrate if this previously imposed threshold can be increased is still pending.

The esterification reaction was also corroborated by an NMR analysis on the mixture of solvents. This analysis had to be conducted solely on the solvents disregarding complete YBCO precursor solutions due to the paramagnetic nature of copper, which hinders a high-quality resolution in standard NMR conditions. For this reason, we analyzed an immediately prepared mixture of HProp and MeOH (50:50) and a mixture of the same solvents with the addition of a 4%_v/v_ MEA. The NMR of the as-prepared solutions promptly showed the difference between the two cases (Figures S15–S18). In the solution where no additive was present, the peak of the ester was identified, indicating the immediate occurrence of Fischer esterification, whereas in the solution in which the amine additive was used, this peak was not observed. The repetition of the measurement on these solutions after 1 week from preparation showed the increase of the ester peak in the case without MEA as well as the appearance of a low-intensity peak of the ester in the solution with the amine additive. By correlating the area of the −CH_3_ peak of the ester to the −CH_3_ peak of MeOH, we could estimate the quantity of ester formed. On the day of preparation, the solution without MEA showed 4% of ester (with respect to MeOH), whereas this increased up to 21% after 8 days. The solution with MEA started at 0% on the day of preparation and increased to only a 4% upon remeasurement after 8 days, confirming that the production rate of H_2_O in solutions is strongly reduced with the amine additive.

**Figure 7:**
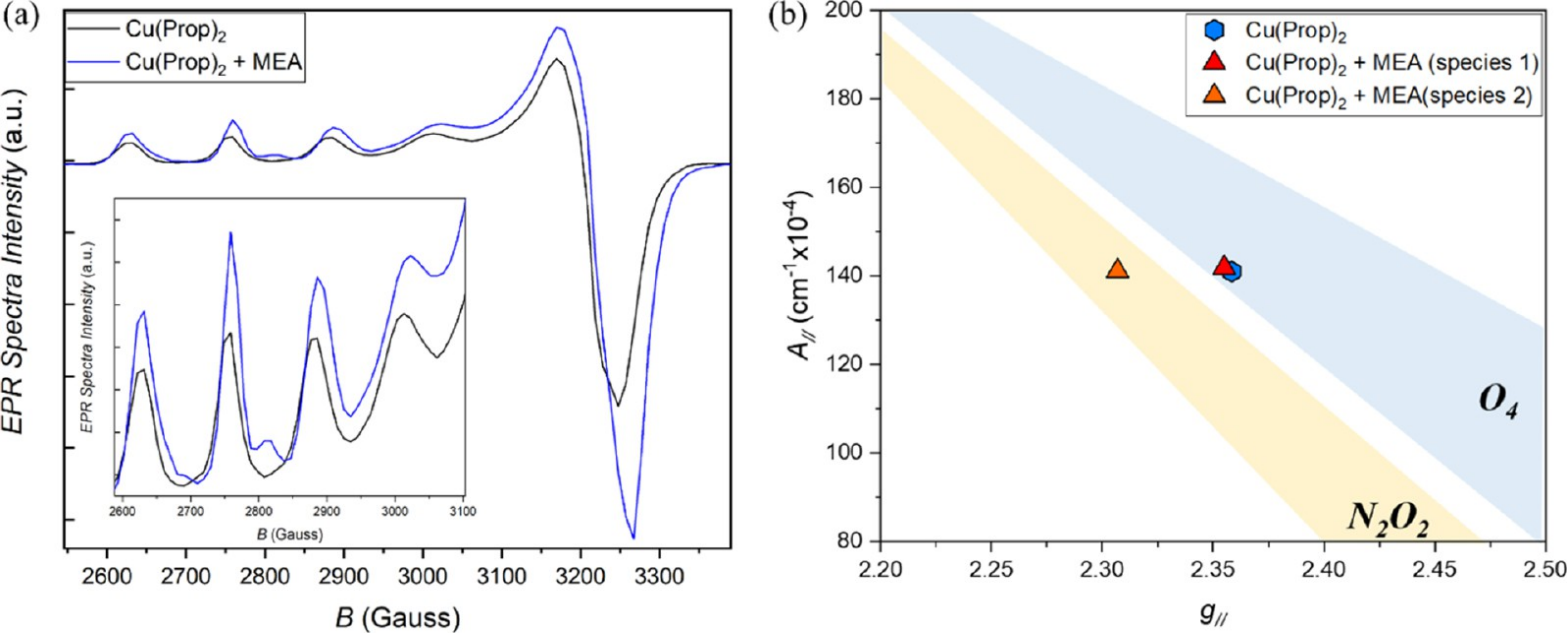
(a) EPR spectra of a mixture of HProp and MeOH (50:50) and Cu(Prop)_2_ without MEA (black) and with 4%_v/v_ MEA (blue). The inset shows the magnified bands. (b) Graph of possible coordination arrangements for various copper complexes depending on *A*_II_ and *g*_II_ values (adapted^62−64^).

Another fundamental aspect to be investigated is the reason for which a higher solution concentration is reached by the addition of MEA, in concurrence with higher solubility of Cu(Prop)_2_ in the solvent mixture. In order to explore the possibility of a complex formation in solution, EPR was used (Figure 7) as, in this context, it can provide valuable information on the interaction between the electrons of the copper atom with those of its neighboring nuclei, the in-plane coordinated ligands. The analysis, as for the previous studies, was conducted on two solutions: Cu(Prop)_2_ in a 50:50 mixture of HProp and MeOH, and the same solution with addition of MEA in a molar ratio of Cu/MEA = 1:0.61 (corresponding to the molar ratio in an optimized solution of 1.75 M + 4%_v/v_ MEA). Both solutions had a concentration of 1 M; therefore, the only difference was the presence of MEA. The EPR spectra display a major difference between the two cases (Figure 7a). In the case of the solution without the amine additive, we obtained the classic EPR spectrum for a Cu^2+^ octahedral elongated complex, with values of *g*_II_ = 2.358 and *A*_II_ = 141 cm^–1^ × 10^–4^. The spectrum obtained for the solution where MEA is added differs distinctly as it is possible to identify the contribution of two species to the spectrum, responsible for an evident splitting of the bands; the first species has values of *g*_II_ = 2.355 and *A*_II_ = 141.9 cm^–1^ × 10^–4^, whereas the second has *g*_II_ = 2.307 and *A*_II_ = 141.1 cm^–1^ × 10^–4^. The graph in Figure 7b (adapted^62−64^) correlates these two values for each case to a specific coordination arrangement for various copper complexes. As expected, the solution without the amine additive shows an in-plane coordination of the copper exclusively to four oxygen atoms, coming from the four propionate groups, as no nitrogen atoms are present in this solution. On the contrary, the solution containing MEA displays the contemporary existence of two species in solution, one in which the copper shows in-plane coordination to four oxygen atoms, as in the previous case, and a second one where the in-plane coordination with the copper is produced by two oxygen atoms and two nitrogen atoms. This demonstrates the formation of a complex with MEA; furthermore, as the MEA is not in stoichiometric quantity with the Cu(Prop)_2_, it gives a reason for the concomitant existence of two species in solution. Therefore, we believe that this may explain the higher solubility of Cu(Prop)_2_ when MEA is employed, allowing for a higher concentration due to the formation of a complex Cu–MEA, more stable in solution than Cu(Prop)_2_ alone. Efforts on crystallizing and identifying this compound are beyond the scope of this article.

The final step to have a full overview on the novelty of these fluorine-free solutions is the study of their decomposition through thermogravimetric analysis coupled to FT-IR (TG-FTIR). The decomposition of this type of metal–organic precursors occurs via the removal of volatile species (small organic molecules such as propionic acid, acetaldehyde) which leave behind a solid residue. TG-FTIR is a technique which allows both to register the mass loss coming from the decomposition of the solutions/films when subjected to a heat treatment as the one used in the pyrolysis process and to analyze the infrared stretching of the volatiles generated in situ. For that study, YBCO precursor solutions with and without the amine additive were analyzed. The solutions without amine had a concentration in sum of salts of 1 M, whereas the solutions with amine were the optimized ones for (3:7) and (2:3) compositions (1.75 M + 4%_v/v_ MEA and 1.75 M + 4.3%_v/v_ MEA, respectively). The samples were prepared through drop casting of a solution on 10 × 10 mm LaAlO_3_ (LAO) substrates. To ensure representativeness between samples of different concentrations, similar initial masses were used (exact mass values are mentioned in the Supporting Information, Section SIV). Thorough thermogravimetric studies and understanding of YBCO precursor solutions have been described earlier for fluorinated^65,66^ and non-fluorinated solutions.^54^ The results from the present solutions indicate that the TG decomposition profile for the amine cases has an evidently less abrupt mass loss than the cases without the amine additive, which instead present an important mass loss around 240–250 °C, corresponding to the end of the Cu(Prop)_2_ decomposition. This temperature range matches with the moment where crack formation is favored^54^ (Figures S19–S22). This particular decomposition profile justifies that films from solutions with amine are strongly robust against crack formation up to the film thickness studied, improving the quality of the layers. Notice that for amine-free solutions, a tendency to generate cracks was observed when the film thickness increases.^38^

Additionally, in both cases (Figure S23), the mass loss is in agreement with the expected one from a dried precursor solution (43% with MEA and 47% without MEA). Since the amount of MEA is very small, the volatiles detected are very similar and are in agreement with the expected ones in a humid O_2_ atmosphere. In particular, decomposition in this atmosphere takes place through evolution of propionic acid coming from the hydrolysis mechanism of the copper and yttrium carboxylates,^54,67^ which in part overlaps with the oxidation path at higher temperatures (220–300 °C) and generates mostly acetaldehyde and CO_2_. The last CO_2_ peaks, in the 300–500 °C temperature range, can be ascribed to the oxidation of the barium salt, which is stable until 280–300 °C.^55^ Similar analysis has been conducted for the case of (2:3) composition solutions (Figure S24). If we compare the (2:3) composition with the (3:7) composition, we can notice that these films (Figures S20 and S22, respectively) are harder to dry, and thus, the propionic acid coming from the initial evaporation stage (before 160 °C) overlaps much more with the same acid coming from the hydrolysis path of the salts in a humid atmosphere. This could be a consequence of the higher viscosity of this solution composition as well as the resulting thickness due to the different deposition method. Finally, notice that the variation of the Cu/Ba ratio affects the amount of CO_2_ evolving: in the decomposition region of the barium salt (300–500 °C), the CO_2_ peaks are more intense in the (2:3) composition (Figure S20) with respect to the (3:7) composition, shown in Figure S22.

### Growth from Optimized Nanocrystalline Precursor Layers

**Figure 8:**
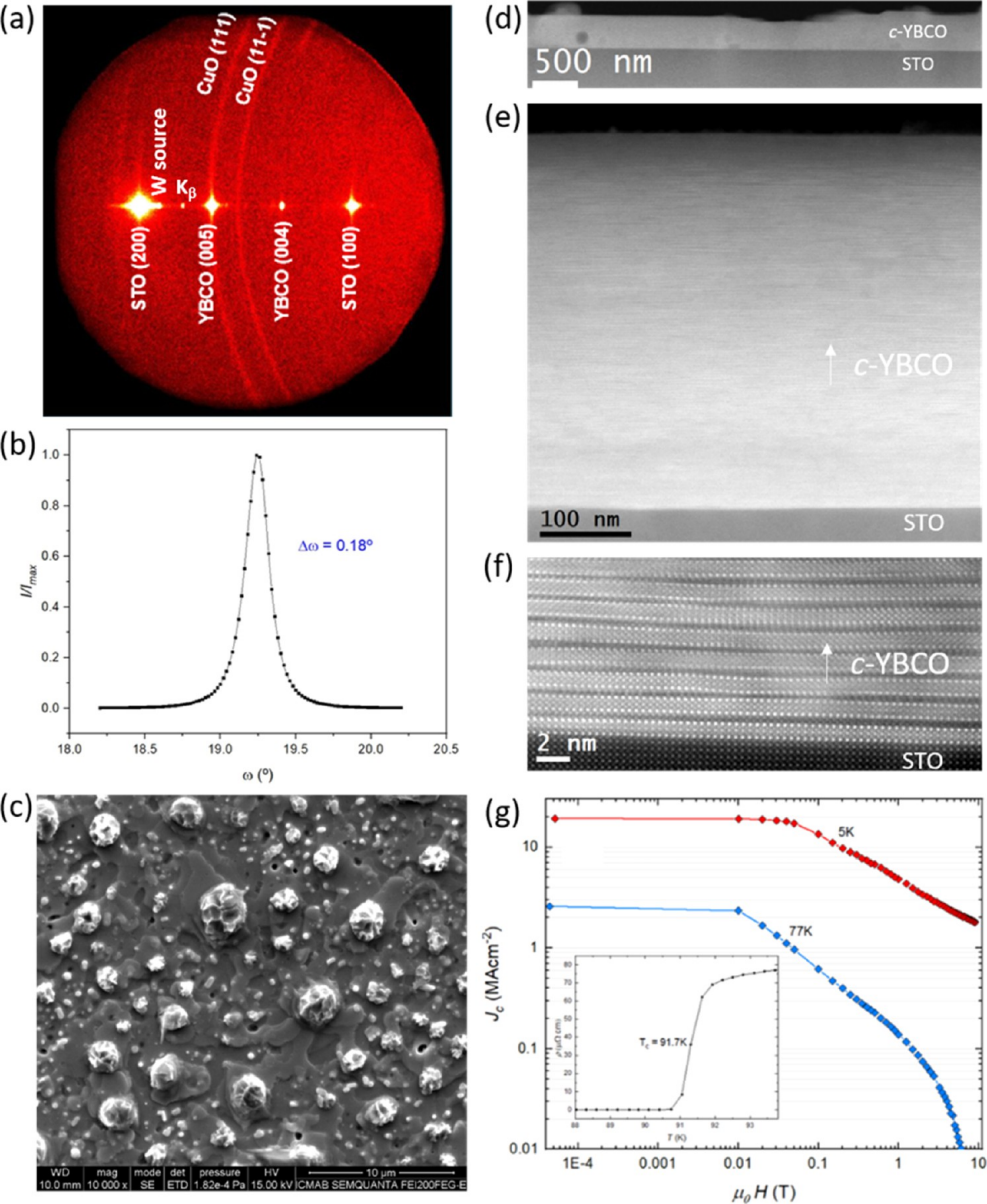
(a) 2D-scan GADDS XRD, (b) ω-scan, (c) SEM image, (d) low-magnification HAADF-STEM image, (e) intermediate-magnification HAADF-STEM image, (f) HR-STEM image of *c*-YBCO/STO interface, and (g) *J*_c_(*H*) (*T*_*c*_ is shown in the inset) of a (3:7) composition *c*-YBCO thin film deposited using 1.75 M + 4%_v/v_ MEA solution, grown at 835 °C, by performing a PO_2_ jump from 10^–5^ to 10^–3^ bar.

Ultimately, samples prepared by using this novel class of optimized fluorine-free propionate-based solutions were grown through the TLAG process in PO_2_ (more details in the Supporting Information, Section SV) using various growth conditions to test for their suitability for the TLAG process. High-performance epitaxial YBCO superconducting layers could be produced in a reproducible manner (Figures 8 and S25), suggesting promising opportunities for further optimization of the TLAG process using these innovative solutions. The details from growth of samples deriving from different solution compositions, TLAG process parameters, and modifications required for thicker films are beyond the scope of this article. However, in this section, we demonstrate that growth of high-performance YBCO superconducting layers through TLAG is possible using the optimized solution and homogeneous nanocrystalline precursor layers described in the previous sections of this article.

Figure 8 shows the example of a (3:7) composition sample, grown through the TLAG PO_2_ route. The texture and quality of the epitaxy are indicative of a high-performance film as shown by 2D-scan GADDS XRD (Figure 8a) and a Δω value of 0.18 (Figure 8b) (a Δφ value of 0.61, not shown here), respectively. The morphology observed through SEM (Figure 8c) shows a flat surface of epitaxial YBCO, with the excess of CuO (coming from the copper excess in this specific composition) expelled to the surface homogeneously distributed and displaying the same morphology on the surface of the entire sample.

The low-magnification cross-sectional HAADF-STEM image (Figure 8d) displays a smooth surface with very few small-sized secondary phases. Figure 8e shows an intermediate-magnification HAADF-STEM image, displaying a dense YBCO film without any pores and with an estimated thickness of ∼400 nm. The HR-STEM image (Figure 8f) of YBCO/STO interface confirms the *c*-axis epitaxial growth of YBCO on STO along with stacking faults due to Y248 intergrowths in the YBCO layer. The critical current density (*J*_c_) (Figure 8g) is high: 20 MA/cm^2^ at 5 K and 2.6 MA/cm^2^ at 77 K for a final thickness of the layer of 400 nm. Contrarily, when a sample which presents unfavorable nanocrystalline precursor phases (deposited from an excess of MEA solution, i.e., 1.75 M + 8%_v/v_ MEA) is grown in the same conditions, the resulting physical properties are seen to be strongly affected by the low epitaxy that the film presents (Figure S26). Nonetheless, for samples deriving from optimized solutions, the main obstacle to current percolation is the presence of secondary phases (EDX maps, Figure S27) creating discontinuities of the epitaxial YBCO layer and therefore preventing higher *J*_c_ values to be obtained. However, upon optimization of the TLAG process, we are certain that high-performance samples of high-thickness YBCO can be produced through the use of these solutions in the innovative and promising framework of TLAG.

## Conclusions

Through a meticulous process of optimization, we have demonstrated the development of a novel class of solutions based on metal propionates of yttrium, barium, and copper, suitable for the preparation of high-performance epitaxial YBCO superconductors.

By synthesizing propionates of yttrium, barium, and copper, we can successfully eliminate the possibility of mixtures of products deriving from the incomplete conversion of acetate precursors in the solvent media. Moreover, the synthetic process is facile, only including one-pot syntheses, resulting in high-purity products with high yields, and, most importantly, it is extremely cost-effective as compared with commercially available acetate precursors.

Making use of these metal-propionate precursors, a novel class of fluorine-free solutions for TLAG-CSD of YBCO films was developed through the introduction of MEA as an additive to our system. We proved the great advantage of the use of MEA in aiding the dissolution of Cu(Prop)_2_ through the formation of a Cu–MEA complex and hence the increase in concentration and viscosity. This subsequently results in an enhancement of the precursor layers’ thickness by a factor 4 with respect to films obtained from solutions in which MEA is not employed. However, it was demonstrated that the optimization of MEA content was crucial to obtain nanoscale homogeneous precursor layers with the necessary characteristics for TLAG: in fact, the extreme case of excess of MEA was a clear illustration of the adverse microstructural conditions for our purpose, even though, if only solution rheology was considered, the excess of MEA enhanced importantly the rapid dissolution of the precursors and increased considerably solution viscosity and thus the thickness of the precursor layers.

Microstructural characterization by TEM analysis was the key element to a deep understanding of the behavior of the system, shedding light on the fundamental data about the microstructure, such as identification and sizes of the nanocrystalline intermediate phases and their homogeneity, spatial distribution, and porosity. All of them have been essential for the successful development of high-quality and high-thickness precursor layers deriving from this novel class of solutions.

Solution rheology and thermal studies unraveled the mechanisms and characteristics of the solutions through the use of various techniques, further highlighting the effectiveness of the amine additive.

Finally, we demonstrated the success of this solution in the growth of TLAG YBCO layers from the texture quality to the superconducting properties, given the nanoscale chemical and microstructural homogeneity of the precursor films obtained with this solution. We believe that further research will lead to a detailed comprehension of the innovative TLAG process for the fabrication of thick REBCO layers and CCs.

## Supporting Information Available

Description of metal propionates’ synthesis, solution preparation procedure, microstructural characterization of precursor layers through TEM analysis, and details on solution rheology and decomposition pathways through TG-FTIR and the TLAG PO_2_ route of precursor layers of different characteristics (PDF)

## References

[ref1] Shiohara Y.; Taneda T.; Yoshizumi M. Overview of Materials and Power Applications of Coated Conductors Project. Jpn. J. Appl. Phys. 2011, 51, 010007 10.1143/jjap.51.010007.

[ref2] Obradors X.; Puig T. Coated Conductors for Power Applications: Materials Challenges. Supercond. Sci. Technol. 2014, 27, 044003 10.1088/0953-2048/27/4/044003.

[ref3] Ito S.; Hashizume H.; Yanagi N.; Tamura H. Advanced High-Temperature Superconducting Magnet for Fusion Reactors: Segment Fabrication and Joint Technique. Fusion Eng. Des. 2018, 136, 239–246. 10.1016/j.fusengdes.2018.01.072.

[ref4] Bednorz J. G.; Müller K. A. Possible highT c superconductivity in the Ba?La?Cu?O system. ZPhys-e.B: Condens. Matter 1986, 64, 189–193. 10.1007/bf01303701.

[ref5] Larbalestier D. C.; Cooley L. D.; Rikel M. O.; Polyanskii A. A.; Jiang J.; Patnaik S.; Cai X. Y.; Feldmann D. M.; Gurevich A.; Squitieri A. A.; Naus M. T.; Eom C. B.; Hellstrom E. E.; Cava R. J.; Regan K. A.; Rogado N.; Hayward M. A.; He T.; Slusky J. S.; Khalifah P.; Inumaru K.; Haas M. Strongly linked current flow in polycrystalline forms of the superconductor MgB2. Nature 2001, 410, 186–189. 10.1038/35065559. 11242073

[ref6] Haran K. S.; Kalsi S.; Arndt T.; Karmaker H.; Badcock R.; Buckley B.; Haugan T.; Izumi M.; Loder D.; Bray J. W.; Masson P.; Stautner E. W. High power density superconducting rotating machines-development status and technology roadmap. Supercond. Sci. Technol. 2017, 30, 123002 10.1088/1361-6668/aa833e.

[ref7] Li L.; Liu W.-D.; Liu Q.; Chen Z.-G. Multifunctional Wearable Thermoelectrics for Personal Thermal Management. Adv. Funct. Mater. 2022, 32, 2200548 10.1002/adfm.202200548.

[ref8] Zhou C.; Lee Y. K.; Yu Y.; Byun S.; Luo Z. Z.; Lee H.; Ge B.; Lee Y. L.; Chen X.; Lee J. Y.; Cojocaru-Mirédin O.; Chang H.; Im J.; Cho S. P.; Wuttig M.; Dravid V. P.; Kanatzidis M. G.; Chung I. Polycrystalline SnSe with a Thermoelectric Figure of Merit Greater than the Single Crystal. Nat. Mater. 2021, 20, 1378–1384. 10.1038/s41563-021-01064-6. 34341524 PMC8463294

[ref9] Rodriguez J. E. YBCO Samples as a Possible Thermoelectric Material. Phys. Status Solidi C 2005, 2, 3605–3608. 10.1002/pssc.200461769.

[ref10] Ahn D.; Kwon O.; Chung W.; Jang W.; Lee D.; Youn S. W.; Byun H.; Youm D.; Semertzidis Y. K. First prototype of a biaxially textured YBa2Cu3O7–x microwave cavity in a high magnetic field for dark matter axion search. 2021, arXiv:2103.14515.

[ref11] Kaya P.; Gregori G.; Baiutti F.; Yordanov P.; Suyolcu Y. E.; Cristiani G.; Wrobel F.; Benckiser E.; Keimer B.; Van Aken P. A.; Habermeier H. U.; Logvenov G.; Maier J. High-Temperature Thermoelectricity in LaNiO3-La2CuO4 Heterostructures. ACS Appl. Mater. Interfaces 2018, 10, 22786–22792. 10.1021/acsami.8b02153. 29927575

[ref12] Wu M. K.; Ashburn J. R.; Torng C. J.; Hor P. H.; Meng R. L.; Gao L.; Huang Z. J.; Wang Y. Q.; Chu C. W. Superconductivity at 93 K in a New Mixed-Phase Y-Ba-Cu-O Compound System at Ambient Pressure. Phys. Rev. Lett. 1987, 58, 908–910. 10.1103/PhysRevLett.58.908. 10035069

[ref13] MacManus-Driscoll J. L.; Wimbush S. C. Processing and Application of High-Temperature Superconducting Coated Conductors. Nat. Rev. Mater. 2021, 6, 587–604. 10.1038/s41578-021-00290-3.

[ref14] Jha A. K.; Matsumoto K. Superconductive REBCO Thin Films and Their Nanocomposites: The Role of Rare-Earth Oxides in Promoting Sustainable Energy. Front. Phys. 2019, 7, 82 10.3389/fphy.2019.00082.

[ref15] Shiohara Y.; Yoshizumi M.; Takagi Y.; Izumi T. Future Prospects of High Tc Superconductors-Coated Conductors and Their Applications. Phys. C 2013, 484, 1–5. 10.1016/j.physc.2012.03.058.

[ref16] Majkic G.; Pratap R.; Xu A.; Galstyan E.; Higley H. C.; Prestemon S. O.; Wang X.; Abraimov D.; Jaroszynski J.; Selvamanickam V. Engineering current density over 5 kA mm–2 at 4.2 K, 14 T in thick film REBCO tapes. Supercond. Sci. Technol. 2018, 31, 10LT01 10.1088/1361-6668/aad844.

[ref17] Molodyk A.; Samoilenkov S.; Markelov A.; Degtyarenko P.; Lee S.; Petrykin V.; Gaifullin M.; Mankevich A.; Vavilov A.; Sorbom B.; Cheng J.; Garberg S.; Kesler L.; Hartwig Z.; Gavrilkin S.; Tsvetkov A.; Okada T.; Awaji S.; Abraimov D.; Francis A.; Bradford G.; Larbalestier D.; Senatore C.; Bonura M.; Pantoja A. E.; Wimbush S. C.; Strickland N. M.; Vasiliev A. Development and large volume production of extremely high current density YBa2Cu3O7 superconducting wires for fusion. Sci. Rep. 2021, 11, 2084 10.1038/s41598-021-81559-z. 33483553 PMC7822827

[ref18] Zhao Y.; Ma L.; Wu W.; Suo H. L.; Grivel J. C. Study on advanced Ce0.9La0.1O2/Gd2Zr2O7 buffer layers architecture towards all chemical solution processed coated conductors. J. Mater. Chem. A 2015, 3, 13275–13282. 10.1039/c5ta00153f.

[ref19] Obradors X.; Puig T.; Ricart S.; Coll M.; Gazquez J.; Palau A.; Granados X. Growth, nanostructure and vortex pinning in superconducting YBa2Cu3O7thin films based on trifluoroacetate solutions. Supercond. Sci. Technol. 2012, 25, 123001 10.1088/0953-2048/25/12/123001.

[ref20] Solovyov M.; Pardo E.; Šouc J.; Gömöry F.; Skarba M.; Konopka P.; Pekarčíková M.; Janovec J. Non-Uniformity of Coated Conductor Tapes. Supercond. Sci. Technol. 2013, 26, 115013 10.1088/0953-2048/26/11/115013.

[ref21] Sánchez-Valdés C. F.; Puig T.; Obradors X. In situstudy through electrical resistance of growth rate of trifluoroacetate-based solution-derived YBa2Cu3O7films. Supercond. Sci. Technol. 2015, 28, 024006 10.1088/0953-2048/28/2/024006.

[ref22] Rijckaert H.; Pollefeyt G.; Sieger M.; Hänisch J.; Bennewitz J.; De Keukeleere K.; De Roo J.; Hühne R.; Bäcker M.; Paturi P.; Huhtinen H.; Hemgesberg M.; Van Driessche I. Optimizing Nanocomposites through Nanocrystal Surface Chemistry: Superconducting YBa2Cu3O7 Thin Films via Low-Fluorine Metal Organic Deposition and Preformed Metal Oxide Nanocrystals. Chem. Mater. 2017, 29, 6104–6113. 10.1021/acs.chemmater.7b02116.

[ref23] Soler L.; Jareño J.; Banchewski J.; Rasi S.; Chamorro N.; Guzman R.; Yáñez R.; Mocuta C.; Ricart S.; Farjas J.; Roura-Grabulosa P.; Obradors X.; Puig T. Ultrafast Transient Liquid Assisted Growth of High Current Density Superconducting Films. Nat. Commun. 2020, 11, 344 10.1038/s41467-019-13791-1. 31953396 PMC6969047

[ref24] Queraltó A.; Banchewski J.; Pacheco A.; Gupta K.; Saltarelli L.; Garcia D.; Alcalde N.; Mocuta C.; Ricart S.; Pino F.; Obradors X.; Puig T. Combinatorial Screening of Cuprate Superconductors by Drop-On-Demand Inkjet Printing. ACS Appl. Mater. Interfaces 2021, 13, 9101–9112. 10.1021/acsami.0c18014. 33576610 PMC7908015

[ref25] Rijckaert H.; Cayado P.; Hänisch J.; Billet J.; Erbe M.; Holzapfel B.; Van Driessche I. Unravelling the Crystallization Process in Solution-Derived YBa2Cu3O7-δ Nanocomposite Films with Preformed ZrO2 Nanocrystals via Definitive Screening Design. J. Phys. Chem. Lett. 2021, 12, 2118–2125. 10.1021/acs.jpclett.1c00135. 33625860

[ref26] Queraltó A.; Pacheco A.; Jiménez N.; Ricart S.; Obradors X.; Puig T. Defining Inkjet Printing Conditions of Superconducting Cuprate Films through Machine Learning. J. Mater. Chem. C 2022, 10, 6885–6895. 10.1039/D1TC05913K. PMC9069570 35665056

[ref27] Kursumovic A.; Tomov R. I.; Hühne R.; MacManus-Driscoll J. L.; Glowacki B. A.; Evetts J. E. Hybrid liquid phase epitaxy processes for YBa2Cu3O7film growth. Supercond. Sci. Technol. 2004, 17, 1215–1223. 10.1088/0953-2048/17/10/024.

[ref28] Lee J.-H.; Lee H.; Lee J.-W.; Choi S.-M.; Yoo S.-I.; Moon S.-H. RCE-DR, a Novel Process for Coated Conductor Fabrication with High Performance. Supercond. Sci. Technol. 2014, 27, 044018 10.1088/0953-2048/27/4/044018.

[ref29] Németh P.; Mugnaioli E.; Gemmi M.; Czuppon G.; Demény A.; Spötl C. A nanocrystalline monoclinic CaCO 3 precursor of metastable aragonite. Sci. Adv. 2018, 4, 1–7. 10.1126/sciadv.aau6178. PMC6291313 30547088

[ref30] Rasi S.; Queraltó A.; Banchewski J.; Saltarelli L.; Garcia D.; Pacheco A.; Gupta K.; Kethamkuzhi A.; Soler L.; Jareño J.; Ricart S.; Farjas J.; Roura-Grabulosa P.; Mocuta C.; Obradors X.; Puig T. Kinetic Control of Ultrafast Transient Liquid Assisted Growth of Solution-Derived YBa 2 Cu3O7-x Superconducting Films. Adv. Sci. 2022, 58, 2203834 10.1002/advs.202203834. PMC9661858 36116124

[ref31] Mitsubishi Chemical Corporation. Karl Fischer Reagents—Technical Manual: Tokyo. http://www.mcckf.com/english/scope.html (accessed February 20, 2019).

[ref32] Rasband W. S. ImageJ; National Institutes of Health, 1997.

[ref33] Bosman M.; Watanabe M.; Alexander D. T. L.; Keast V. J. Mapping Chemical and Bonding Information Using Multivariate Analysis of Electron Energy-Loss Spectrum Images. Ultramicroscopy 2006, 106, 1024–1032. 10.1016/j.ultramic.2006.04.016. 16876322

[ref34] Foltyn S. R.; Jia Q. X.; Arendt P. N.; Kinder L.; Fan Y.; Smith J. F. Relationship between film thickness and the critical current of YBa2Cu3O7−δ-coated conductors. Appl. Phys. Lett. 1999, 75, 3692–3694. 10.1063/1.125431.

[ref35] Izumi T.; Yoshizumi M.; Miura M.; Sutoh Y.; Nakanishi T.; Nakai A.; Ichikawa Y.; Yamada Y.; Goto T.; Yajima A.; Aoki Y.; Hasegawa T.; Shiohara Y. Research and Development of Reel-to-Reel TFA-MOD Process for Coated Conductors. Phys. C 2008, 468, 1527–1530. 10.1016/j.physc.2008.05.270.

[ref36] Pop C.; Barusco P.; Vlad R.; Queralto A.; Gupta K.; Almog B.; Saraf A.; Deutscher G.; Granados X.; Puig T.; Obradors X. High critical current solution derived YBa2Cu3O7 films grown on sapphire. Supercond. Sci. Technol. 2022, 35, 054007 10.1088/1361-6668/ac5be9.

[ref37] Palmer X.; Pop C.; Eloussifi H.; Villarejo B.; Roura P.; Farjas J.; Calleja A.; Palau A.; Obradors X.; Puig T.; Ricart S. Solution design for low-fluorine trifluoroacetate route to YBa2Cu3O7films. Supercond. Sci. Technol. 2015, 29, 024002 10.1088/0953-2048/29/2/024002.

[ref38] Villarejo B.; Pop C.; Ricart S.; Mundet B.; Palau A.; Roura-Grabulosa P.; Farjas J.; Puig T.; Obradors X. Pyrolysis study of solution-derived superconducting YBa2Cu3O7 films: disentangling the physico-chemical transformations. J. Mater. Chem. C 2020, 8, 10266–10282. 10.1039/d0tc01846e.

[ref39] Villarejo B.; Pino F.; Pop C.; Ricart S.; Vallès F.; Mundet B.; Palau A.; Roura-Grabulosa P.; Farjas J.; Chamorro N.; Yáñez R.; Granados X.; Puig T.; Obradors X. High Performance of Superconducting YBa2Cu3O7 Thick Films Prepared by Single-Deposition Inkjet Printing. ACS Appl. Electron. Mater. 2021, 3, 3948–3961. 10.1021/acsaelm.1c00513.

[ref40] Vermeir P.; Feys J.; Schaubroeck J.; Verbeken K.; Lommens P.; Van Driessche I. Influence of Sintering Conditions in the Preparation of Acetate-Based Fluorine-Free CSD YBCO Films Using a Direct Sintering Method. Mater. Res. Bull. 2012, 47, 4376–4382. 10.1016/j.materresbull.2012.09.033.

[ref41] Schoofs B.; Cloet V.; Vermeir P.; Schaubroeck J.; Hoste S.; Driessche I. A water-based sol-gel technique for chemical solution deposition of (RE)Ba2Cu3O7–y(RE = Nd and Y) superconducting thin films. Supercond. Sci. Technol. 2006, 19, 1178–1184. 10.1088/0953-2048/19/11/015.

[ref42] Lu F.; Kametani F.; Hellstrom E. E. Film growth of BaZrO3-doped YBa2Cu3O7−δby using fluorine-free metal-organic deposition. Supercond. Sci. Technol. 2011, 25, 015011 10.1088/0953-2048/25/1/015011.

[ref43] Lei L.; Zhao G.; Zhao J.; Xu H. Water-Vapor-Controlled Reaction for Fabrication of YBCO Films by Fluorine-Free Sol-Gel Process. IEEE Trans. Appl. Supercond. 2010, 20, 2286–2293. 10.1109/TASC.2010.2050589.

[ref44] Zhao Y.; Torres P.; Tang X.; Norby P.; Grivel J.-C. Growth of Highly Epitaxial YBa2Cu3O7−δ Films from a Simple Propionate-Based Solution. Inorg. Chem. 2015, 54, 10232–10238. 10.1021/acs.inorgchem.5b01486. 26473556

[ref45] Zhao Y.; Chu J.; Qureishy T.; Wu W.; Zhang Z.; Mikheenko P.; Johansen T. H.; Grivel J.-C. Structural and superconducting characteristics of YBa2Cu3O7 films grown by fluorine-free metal-organic deposition route. Acta Mater. 2018, 144, 844–852. 10.1016/j.actamat.2017.11.050.

[ref46] Chu J.; Zhao Y.; Khan M. Z.; Tang X.; Wu W.; Shi J.; Wu Y.; Huhtinen H.; Suo H.; Jin Z. Insight into the Interfacial Nucleation and Competitive Growth of YBa2Cu3O7−δ Films as High-Performance Coated Conductors by a Fluorine-Free Metal-Organic Decomposition Route. Cryst. Growth Des. 2019, 19, 6752–6762. 10.1021/acs.cgd.9b01120.

[ref47] Zuo J. L.; Zhao Y.; Wu W.; Chu J. Y.; Wu X. Y.; Zhang Z. W.; Hong Z. Y.; Jin Z. J. Intermediate Phase Evolution of YBCO Superconducting Films Fabricated by Fluorine Free MOD Method. J. Phys.: Conf. Ser. 2018, 1054, 012010 10.1088/1742-6596/1054/1/012010.

[ref48] Mos R. B.; Nasui M.; Petrisor M. S.; Gabor R.; Varga L.; Ciontea T.; Petrisor T. S. Synthesis, crystal structure and thermal decomposition study of a new barium acetato-propionate complex. J. Anal. Appl. Pyrolysis 2011, 92, 445–449. 10.1016/j.jaap.2011.08.007.

[ref49] Jareño Cerulla J. Transient Liquid Assisted Growth of Superconducting Nanocomposite Films. PhD Thesis, Universitat Autònoma de Barcelona, 2020.

[ref50] Kato M.; Jonassen H. B.; Fanning J. C. Copper(II) Complexes with Subnormal Magnetic Moments. Chem. Rev. 1964, 64, 99–128. 10.1021/cr60228a003.

[ref51] Rasi S. Advanced Thermal Analysis of REBCO Superconductor Precursor Films and Functional Oxides. PhD Thesis, Universitat de Girona, 2019.

[ref52] Li M.; Cayado P.; Erbe M.; Jung A.; Hänisch J.; Holzapfel B.; Liu Z.; Cai C. Rapid Pyrolysis of SmBa2Cu3O7-δ Films in CSD-MOD Using Extremely-Low-Fluorine Solutions. Coatings 2020, 10, 31 10.3390/coatings10010031.

[ref53] Vermeir P.; Cardinael I.; Schaubroeck J.; Verbeken K.; Bäcker M.; Lommens P.; Knaepen W.; D’haen J.; De Buysser K.; Van Driessche I. Elucidation of the Mechanism in Fluorine-Free Prepared YBa2Cu3O7−δ Coatings. Inorg. Chem. 2010, 49, 4471–4477. 10.1021/ic9021799. 20405962

[ref54] Rasi S.; Soler L.; Jareño J.; Banchewski J.; Guzman R.; Mocuta C.; Kreuzer M.; Ricart S.; Roura-Grabulosa P.; Farjas J.; Obradors X.; Puig T. Relevance of the Formation of Intermediate Non-Equilibrium Phases in YBa2Cu3O7-x Film Growth by Transient Liquid-Assisted Growth. J. Phys. Chem. C 2020, 124, 15574–15584. 10.1021/acs.jpcc.0c03859.

[ref55] Rasi S.; Ricart S.; Obradors X.; Puig T.; Roura-Grabulosa P.; Farjas J. Radical and Oxidative Pathways in the Pyrolysis of a Barium Propionate-Acetate Salt. J. Anal. Appl. Pyrolysis 2019, 141, 104640 10.1016/j.jaap.2019.104640.

[ref56] Verdonk A. H.; Broersma A. Thermal decomposition of barium oxalate hemihydrate BaC2O4·0.5H2O. 5H_2_O. Thermochim. Acta 1973, 6, 95–110. 10.1016/0040-6031(73)80009-7.

[ref57] Fick A. Ueber Diffusion. Ann. Phys. 1855, 170, 59–86. 10.1002/andp.18551700105.

[ref58] Cayado P.; Mundet B.; Eloussifi H.; Vallés F.; Coll M.; Ricart S.; Gázquez J.; Palau A.; Roura P.; Farjas J.; Puig T.; Obradors X. Epitaxial superconducting GdBa2Cu3O7−δ /Gd2O3 nanocomposite thin films from advanced low-fluorine solutions. Supercond. Sci. Technol. 2017, 30, 125010 10.1088/1361-6668/aa8ffe.

[ref59] Calleja A.; Ricart S.; Palmer X.; Luccas R. F.; Puig T.; Obradors X. Water Determination of Precursor Solutions with Oxidant Cations by the Karl Fischer Method: The YBCO-TFA Case. J. Sol-Gel Sci. Technol. 2010, 53, 347–352. 10.1007/s10971-009-2100-5.

[ref60] Fischer E.; Speier A. B. Darstellung der Ester. Dass. Ber. Dtsch. Chem. Ges. 1895, 28, 3252–3258. 10.1002/cber.189502803176.

[ref61] Bender M. L. Mechanisms of Catalysis of Nucleophilic Reactions of Carboxylic Acid Derivatives. Am. Chem. Soc. 1960, 60, 53–113. 10.1021/cr60203a005.

[ref62] Rasia R. M.; Bertoncini C. W.; Marsh D.; Hoyer W.; Cherny D.; Zweckstetter M.; Griesinger C.; Jovin T. M.; Fernández C. O. Structural characterization of copper(II) binding to α-synuclein: Insights into the bioinorganic chemistry of Parkinson’s disease. PNAS 2005, 102, 4294–4299. 10.1073/pnas.0407881102. 15767574 PMC555498

[ref63] Sakaguchi U.; Addison A. W. Spectroscopic and redox studies of some copper(II) complexes with biomimetic donor atoms: implications for protein copper centres. J. Chem. Soc., Dalton Trans. 1979, 600–608. 10.1039/DT9790000600.

[ref64] Peisach J.; Blumberg W. E. Structural Implications Derived from the Analysis of Electron Paramagnetic Resonance Spectra of Natural and Artificial Copper Proteins. Arch. Biochem. Biophys. 1974, 165, 691–708. 10.1016/0003-9861(74)90298-7. 4374138

[ref65] Eloussifi H.; Farjas J.; Roura P.; Ricart S.; Puig T.; Obradors X.; Dammak M. Thermoanalytical Study of the Decomposition of Yttrium Trifluoroacetate Thin Films. Thin Solid Films 2013, 545, 200–204. 10.1016/j.tsf.2013.07.082.

[ref66] Eloussifi H.; Farjas J.; Roura P.; Ricart S.; Puig T.; Obradors X.; Dammak M. Thermal Decomposition of Barium Trifluoroacetate Thin Films. Thermochim. Acta 2013, 556, 58–62. 10.1016/j.tca.2013.01.022.

[ref67] Rasi S.; Ricart S.; Obradors X.; Puig T.; Roura P.; Farjas J. Thermal Decomposition of Yttrium Propionate: Film and Powder. J. Anal. Appl. Pyrolysis 2018, 133, 225–233. 10.1016/j.jaap.2018.03.021.

