# Supplementary material for: Chemical and Microstructural Nanoscale Homogeneity in Superconducting YBa2Cu3O7–x Films Derived from Metal-Propionate Fluorine-free Solutions
Source: ACS Appl Mater Interfaces. 2022 Oct 21;14(43):48582–97. doi: 10.1021/acsami.2c11414 (PMC9634695; doi:10.1021/acsami.2c11414)
Supplement: Supplementary file 1 — am2c11414_si_001.pdf [file am2c11414_si_001.pdf]

Supporting Information

Chemical and microstructural nanoscale homogeneity in superconducting $YBa_2Cu_3O_{7-x}$ films derived from metal-propionate fluorine-free solutions

Lavinia Saltarelli, ⊥[a] Kapil Gupta, ⊥*[a] Silvia Rasi, [a] Aiswarya Kethamkuzhi, [a] Albert Queraltó, [a] Diana Garcia, [a] Joffre Gutierrez, [a] Jordi Farjas, [b] Pere Roura-Grabulosa, [b] Susagna Ricart, [a] Xavier Obradors, [a] Teresa Puig *[a]

[a] *Institut de Ciència de Materials de Barcelona, ICMAB-CSIC, Campus de la UAB, 08193 Bellaterra, Catalonia, Spain*

[b] *GRMT, Department of Physics, University of Girona, E17071-Girona, Catalonia, Spain*

⊥Authors equally contributed

*Corresponding authors: Dr. Kapil Gupta (kgupta@icmab.es), Prof. Teresa Puig (teresa.puig@icmab.es)

Table of Contents

Section I. Synthesis of Metal Propionates Precursors………………………..…page S-3

Section II. Solution Preparation……………………………………………page S-10

Section III. Nanocrystalline Layers' optimization and Microstructural Analysis…………………………………………………………………………page S-11

Section IV. Solution Rheological Analysis…………………………………..page S-18

Section V. YBCO Growth through TLAG……………………………….....page S-30

Section I. Synthesis of Metal Propionates Precursors

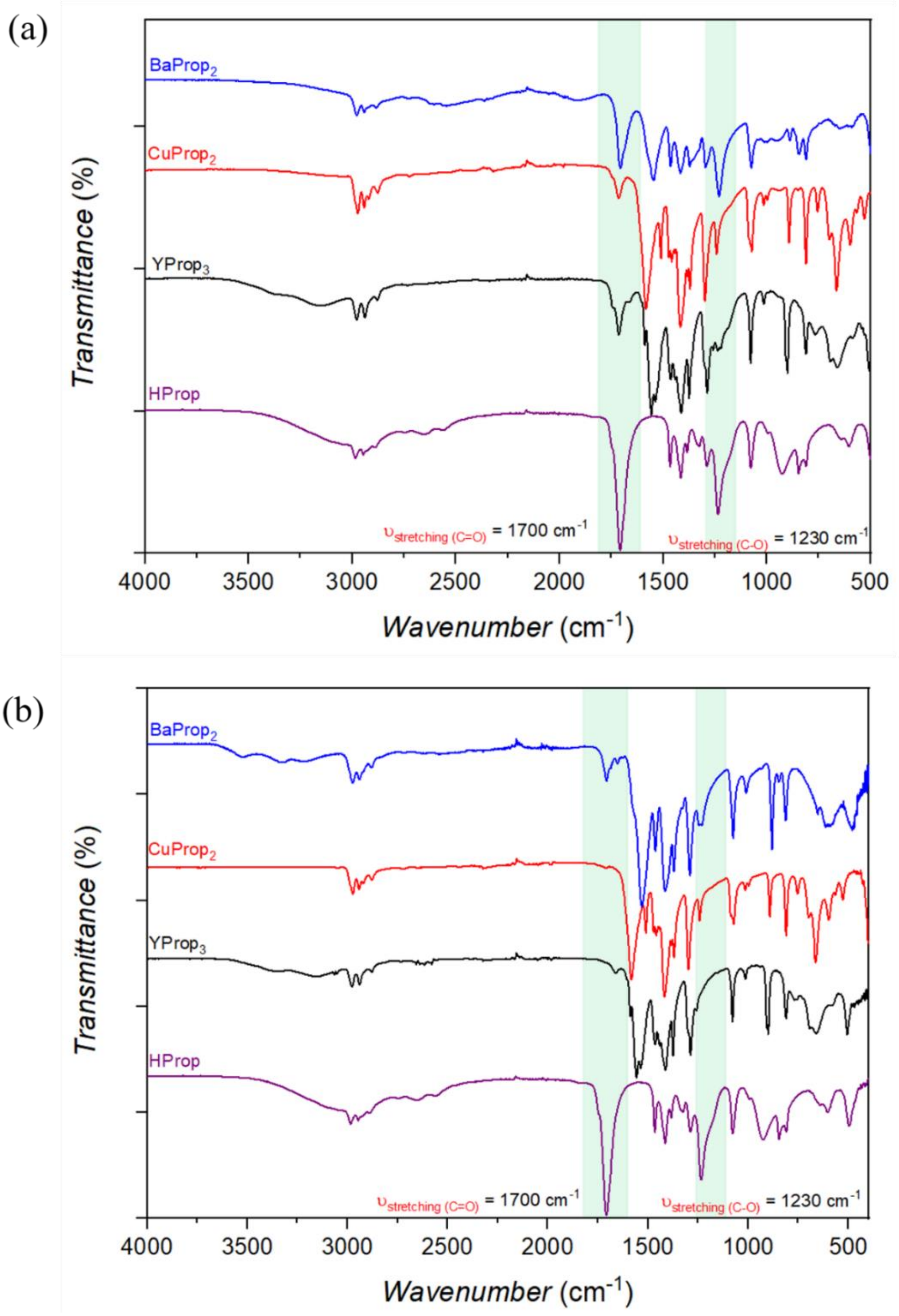

Figure S1. (a) ATR FT-IR of as-synthesized powders (before washing procedure), (b) ATR FT-IR of powders (after washing procedure), with ATR FT-IR of HProp. The peaks due to the presence of residual solvent (HProp), specifically the peaks at 1700 cm^{-1} and 1230 cm^{-1} (respectively the stretching mode of the *C=O* group and the stretching mode of *C-O*), are seen to disappear after the washing procedure, confirming the successful elimination of residual solvent.

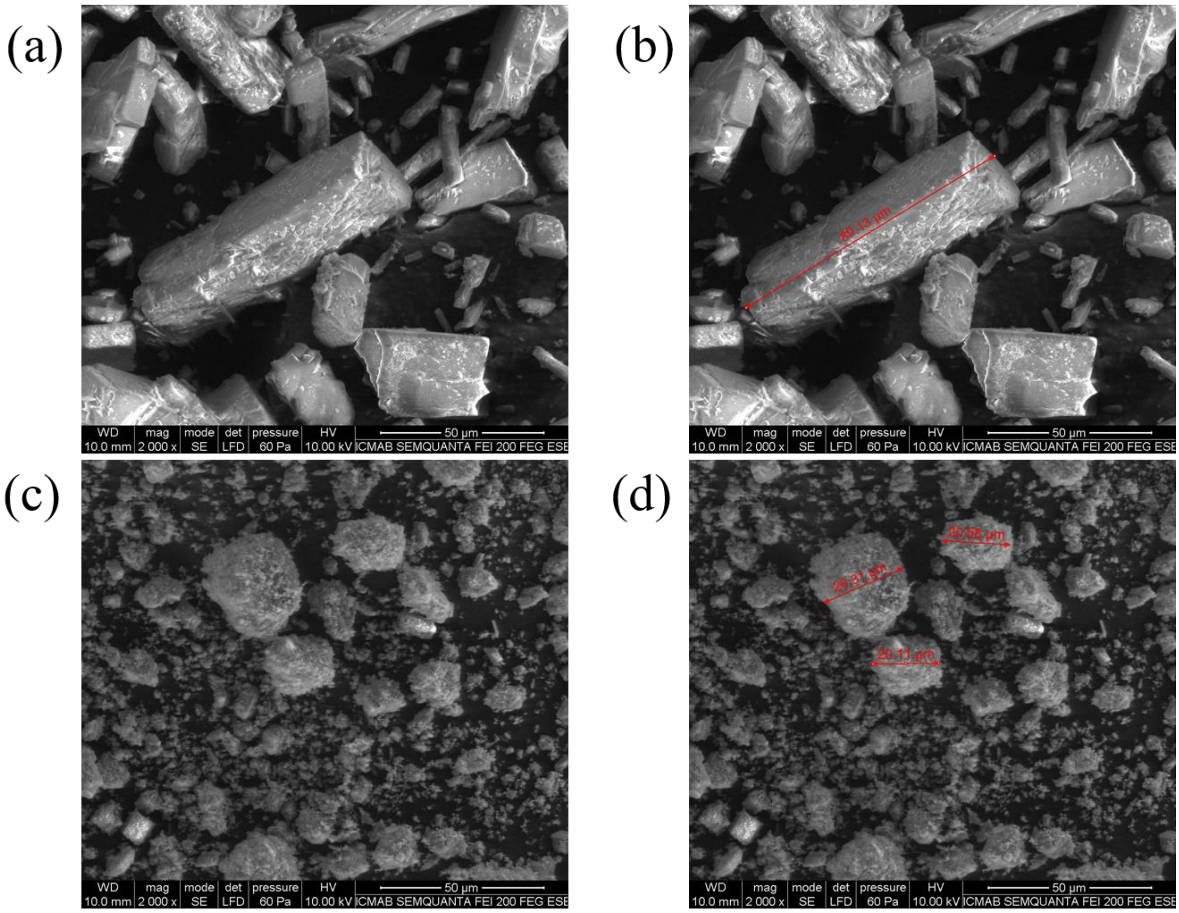

Figure S2. SEM of Cu(Prop)$_2$ final powder product, manually crushed ((a) and (b)) compared to mechanical grinding through ball milling ((c) and (d)).

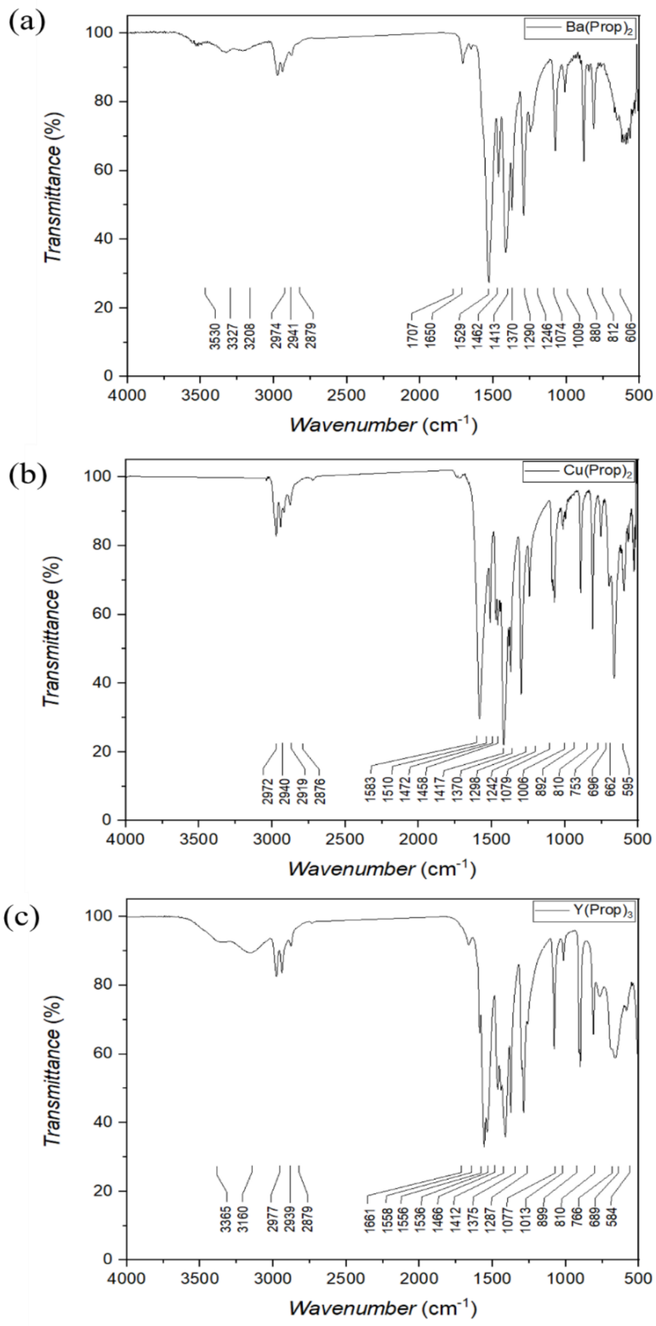

Figure S3. ATR FT-IR of final powder products of (a) $Ba(Prop)_2$, (b) $Cu(Prop)_2$, (c) $Y(Prop)_3$. The three peaks in the range 2980-2870 cm^{-1} are characteristic of the aliphatic chain of the propionate group, present in the three spectra. It can be noted that $Cu(Prop)_2$ is anhydrous, while the presence of broad bands in the range 3000-3500 cm^{-1} for the spectra relative to $Ba(Prop)_2$ and $Y(Prop)_3$ due to *-OH* groups confirm the presence of water in these products. The symmetric and asymmetric vibration of COO^- groups are identified, respectively, around 1550 cm^{-1} and 1460 cm^{-1}, varying due to the different coordination with each metal. Bands around 1079 cm^{-1} in the three spectra are relative to the $-CH_2-CH_3$ deformation vibration mode.

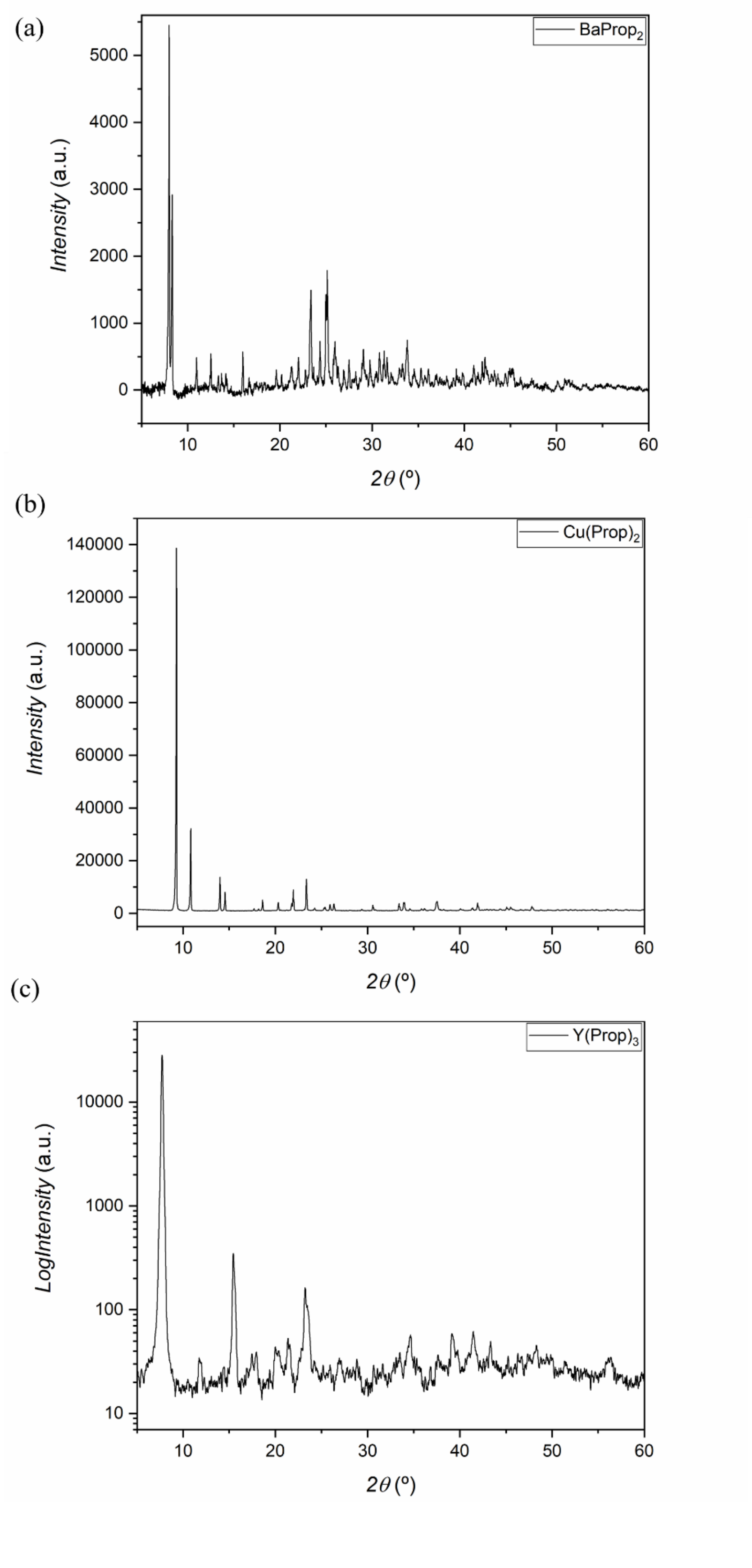

Figure S4. XRD patterns of final powder products of (a) Cu(Prop)$_2$, (b) Ba(Prop)$_2$, (c) Y(Prop)$_3$. All peaks can be identified by the expected metal-propionates.

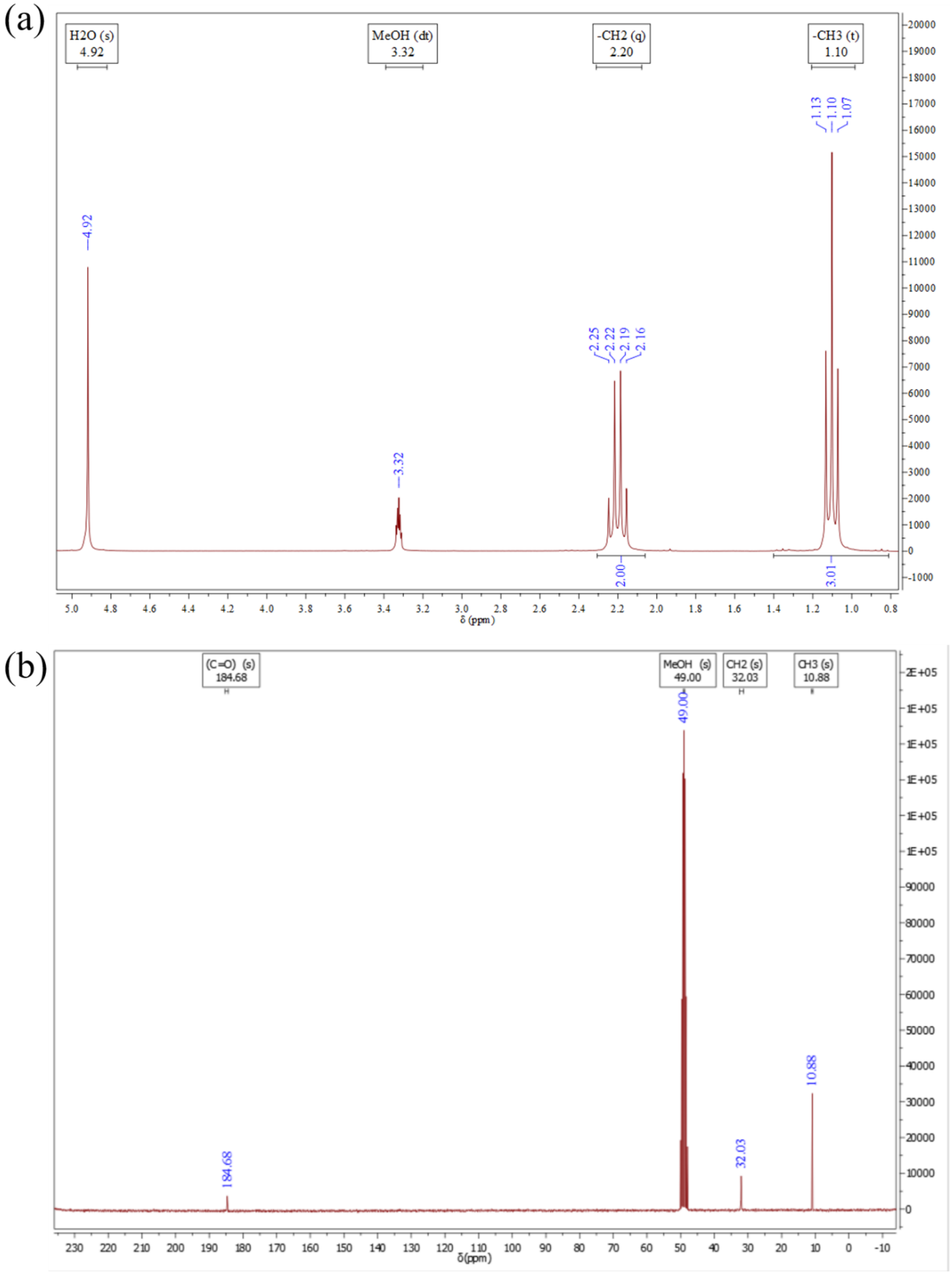

Figure S5. NMR spectra of $Ba(Prop)_2$, in (a) the ^{1}H and in (b) the ^{13}C Both spectra show the presence of only propionate groups.

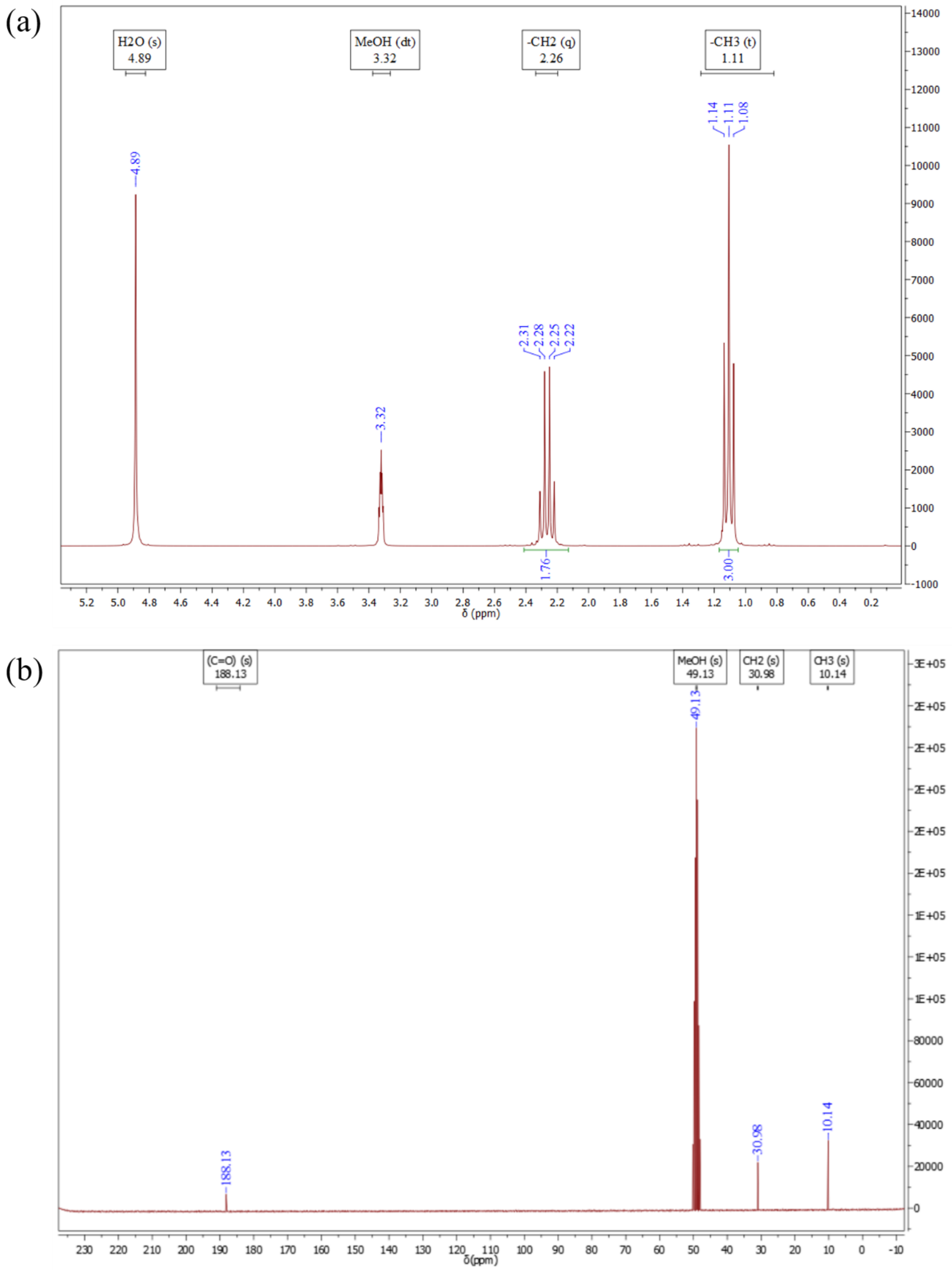

Figure S6. NMR spectra of Y(Prop)$_3$, in (a) the ^{1}H and in (b) the ^{13}C. Both spectra show the presence of only propionate groups.

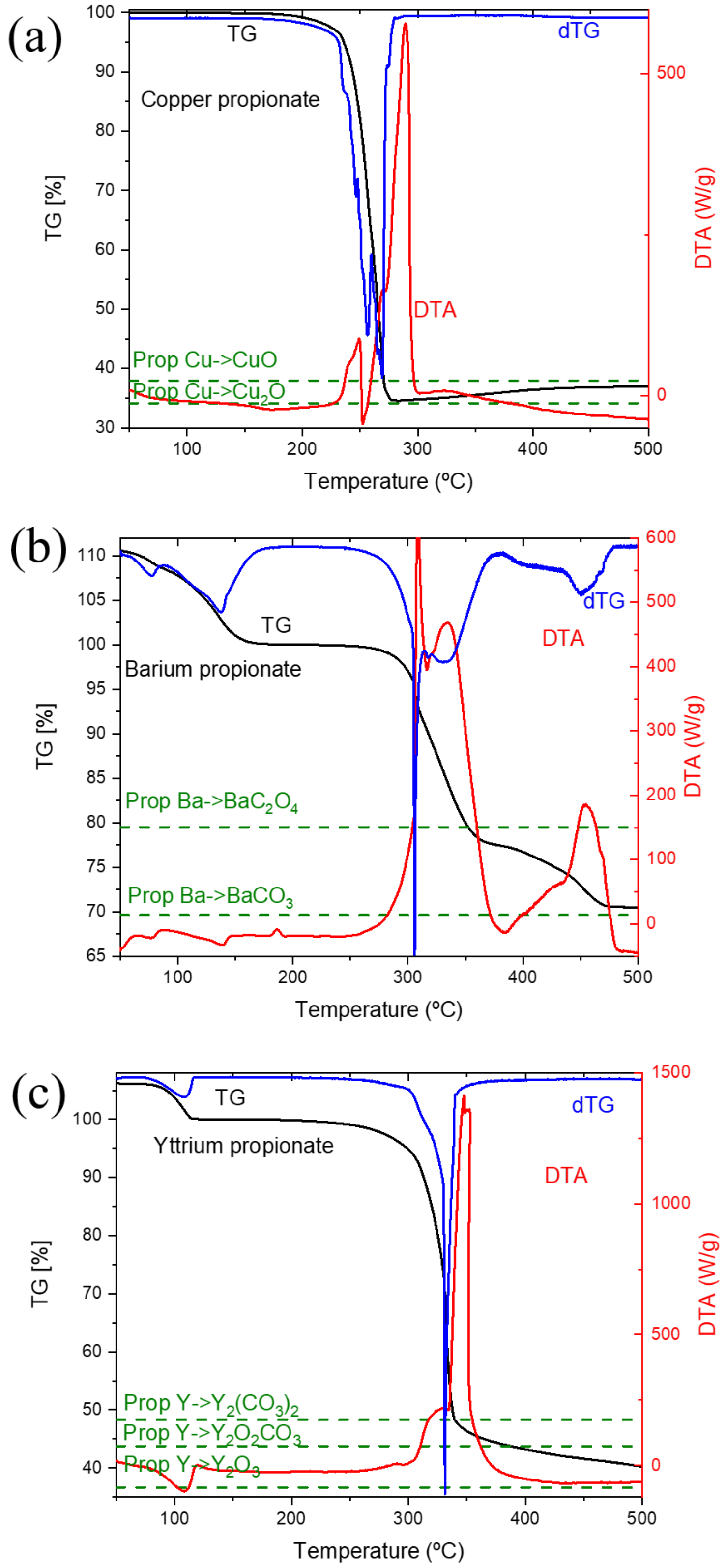

Figure S7. TGA, DTA, and dTG of, (a) $CuProp_2$, (b) $Ba(Prop)_2$, (c) $Y(Prop)_3$ powders performed in dry air at 5K/min. Decomposition of the three powder products follows expected mechanisms to form the oxide products for $Y(Prop)_3$ and $Cu(Prop)_2$, and $BaCO_3$ for the $Ba(Prop)_2$, in agreement with S. Rasi et al.[1–3]

Section II. Solution Preparation

$Ba(Prop)_2$ is the first to be added to the mixture of solvents, showing a fast dissolution, with the solution to appear transparent once dissolution is completed. $Cu(Prop)_2$ is then added, and heated at 30 ºC. Subsequently, $Y(Prop)_3$ is added.

Complete dissolution of all the precursors was achieved yielding an intense blue coloured solution. The solvents mixture was adjusted to 50:50 of solvents (as previously specified), and filtered. The final solution was stored in a sealed vial with Ar atmosphere.

Section III. Nanocrystalline Layers' optimization and Microstructural Analysis

The solution optimization process started after the realization that a 1 M solution with no additive only yielded precursor layers of 100 nm in thickness (Figure S7(a)); the need to move the TLAG process to thicker films for possible industrial applications implied the use of MEA, as described in the main text. Therefore, different values of $\%_{v/v}$ MEA were added to the solution of 1 M in sum of salts, exhibiting a rapid dissolution of $Cu(Prop)_2$, suggesting an interaction between $Cu(Prop)_2$ and the amine additive. Most importantly, the thickness of the resulting precursor layer was strongly increased indicating it as useful for the scope of our main objective. However, the quality of the precursor layer was far from optimal, displaying a rough surface with numerous defects (Figure S8(b) and S8(c)). Therefore, a fine-tuning of solution concentration and MEA content was pivotal for films of the desired quality. Through a finer adjustment of the conditions, high quality precursor films with 400±50 nm thick layers by using a solution of 1.75 M with $4\%_{v/v}$ MEA (Figure S8(d)) found to be the optimal quantity.

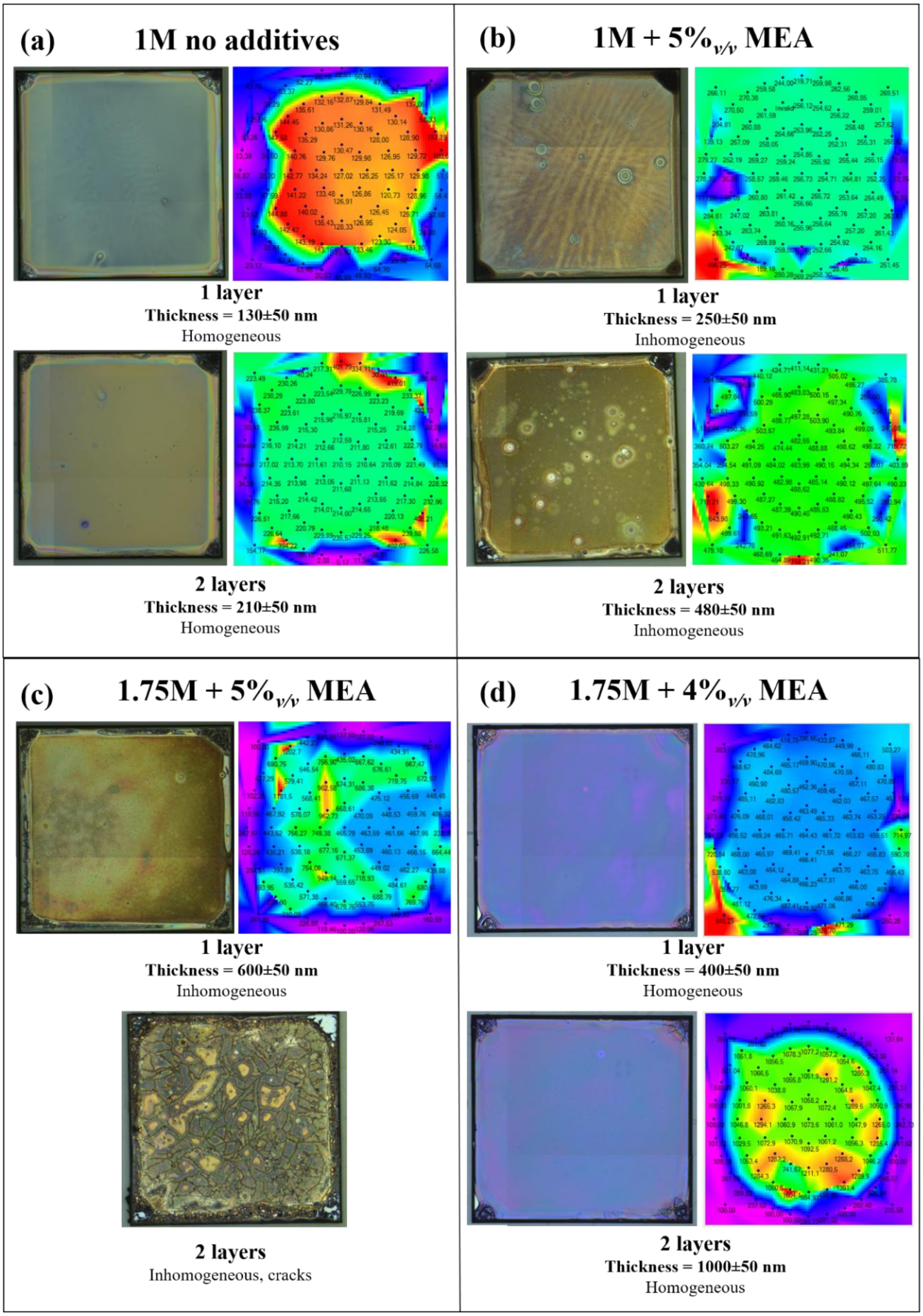

Figure S8. (a)-(d) Optimization process for (3:7) composition solutions varying MEA $\%_{v/v}$, showing optical microscope (OM) images of precursor layers and their thicknesses measured through reflectometry.

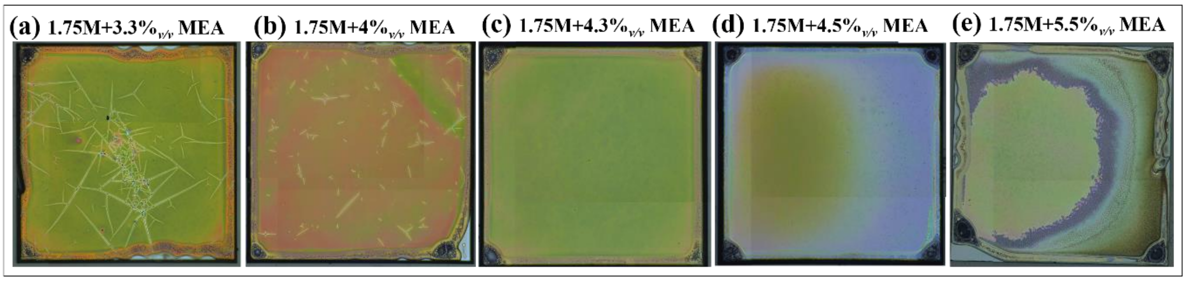

Figure S9. (a)-(e) OM images showing the MEA %$_{v/v}$ optimization process for samples of (2:3) composition. Notice that the sample with 4.3% $_{v/v}$ MEA reaches the best result.

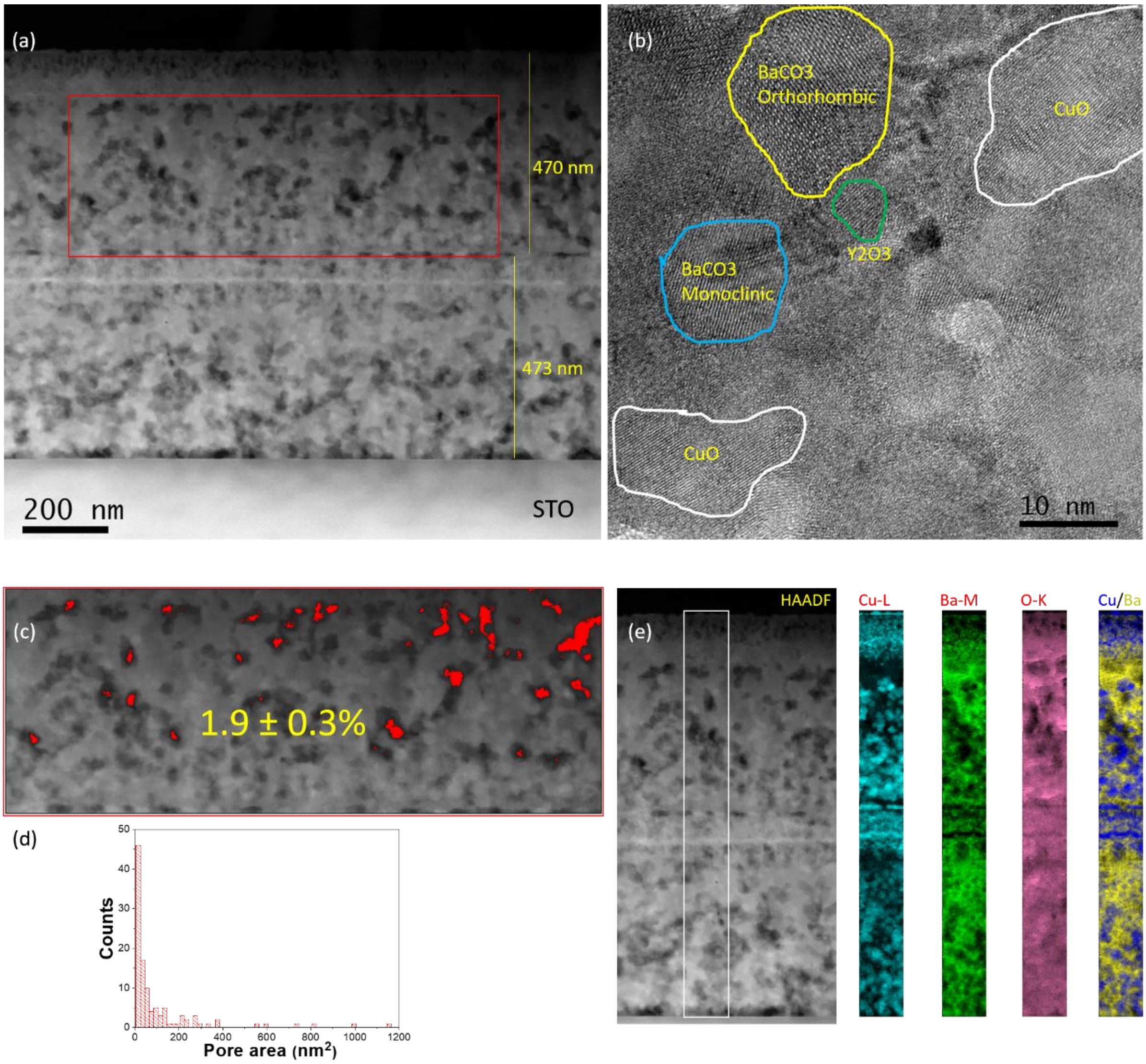

Figure S10. (a) Cross-sectional STEM-HAADF image of a two layer (2:3) precursor thin film deposited using 1.75 M+4.3%$_{v/v}$ MEA solution, displaying the similar thicknesses of individual layers to be ~ 470 nm. (b) HR-TEM image displaying the individual sizes and the presence of all precursor phases in the same area, exhibiting a homogeneous distribution. (c) The pore density analysis using ImageJ from the red rectangular region in (a), where pores are coloured in red for quantification. (d) Histogram of pore area used to calculate the average pore size in Figure 5f. (e) Elemental EELS maps of Cu-L edge, Ba-M edge, O-K edge, and composite EELS map of Cu (blue)-Ba (yellow), from red rectangular region in STEM-HAADF image in (d).

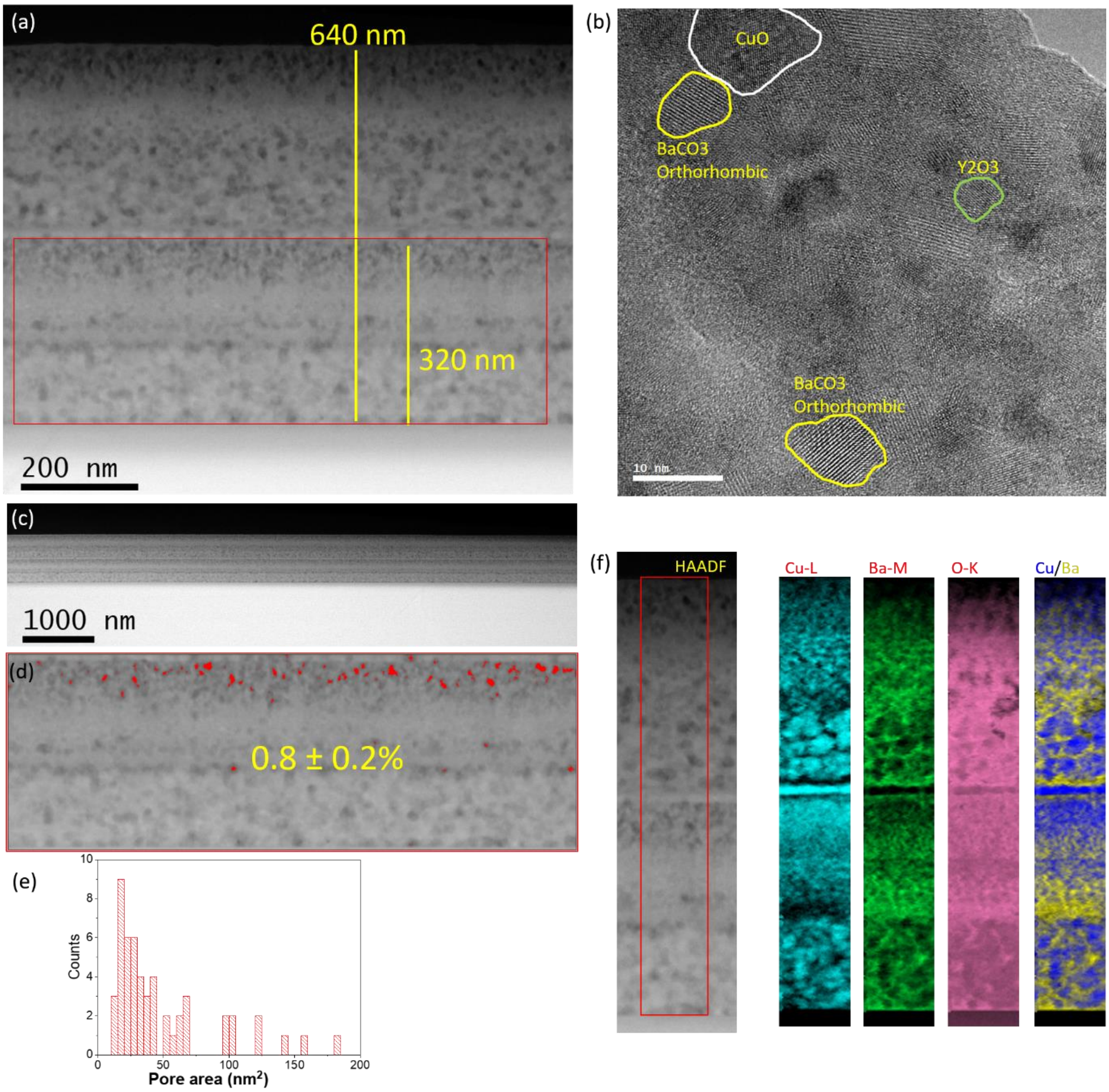

Figure S11. (a) Cross-sectional low magnification STEM-HAADF image of a two layer (4:11) precursor thin film deposited using 1.75 M+4%$_{v/v}$ MEA solution, confirming the smooth surface. (b) HR-TEM image displaying the individual sizes and the presence of all precursor phases in the same area, exhibiting a homogeneous distribution. (c) Cross-sectional STEM-HAADF image exhibiting both layers with similar thicknesses and the total thickness to be ~ 640 nm. (d) The pore density analysis using ImageJ from the red rectangular region in (c), where pores are coloured in red for quantification. (e) Histogram of pore area used to calculate the average pore size in Figure 5f. (f) Elemental EELS maps of Cu-L edge, Ba-M edge, O-K edge, and composite EELS map of Cu (blue)-Ba (yellow), from red rectangular region in STEM-HAADF image in (e).

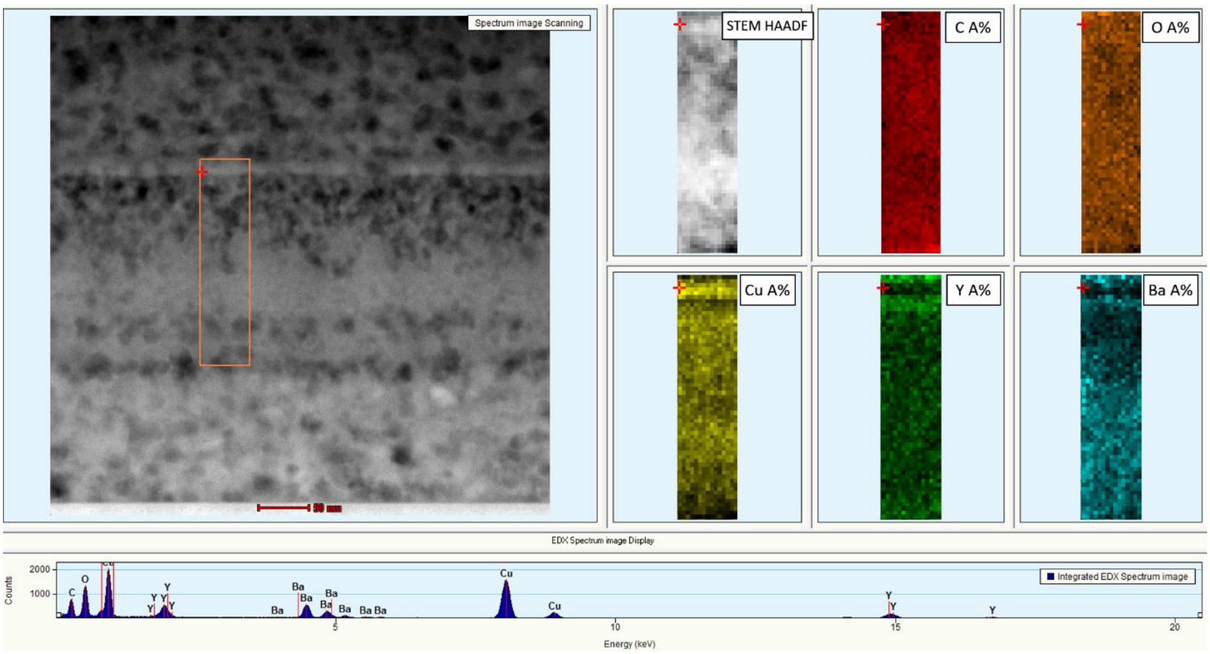

Figure S12. STEM-EDX cross-sectional elemental maps of Cu, Y, and Ba showing homogeneous distribution of precursor phases in a (3:7) composition precursor thin film deposited using 1.75 M+4%$_{v/v}$ MEA solution.

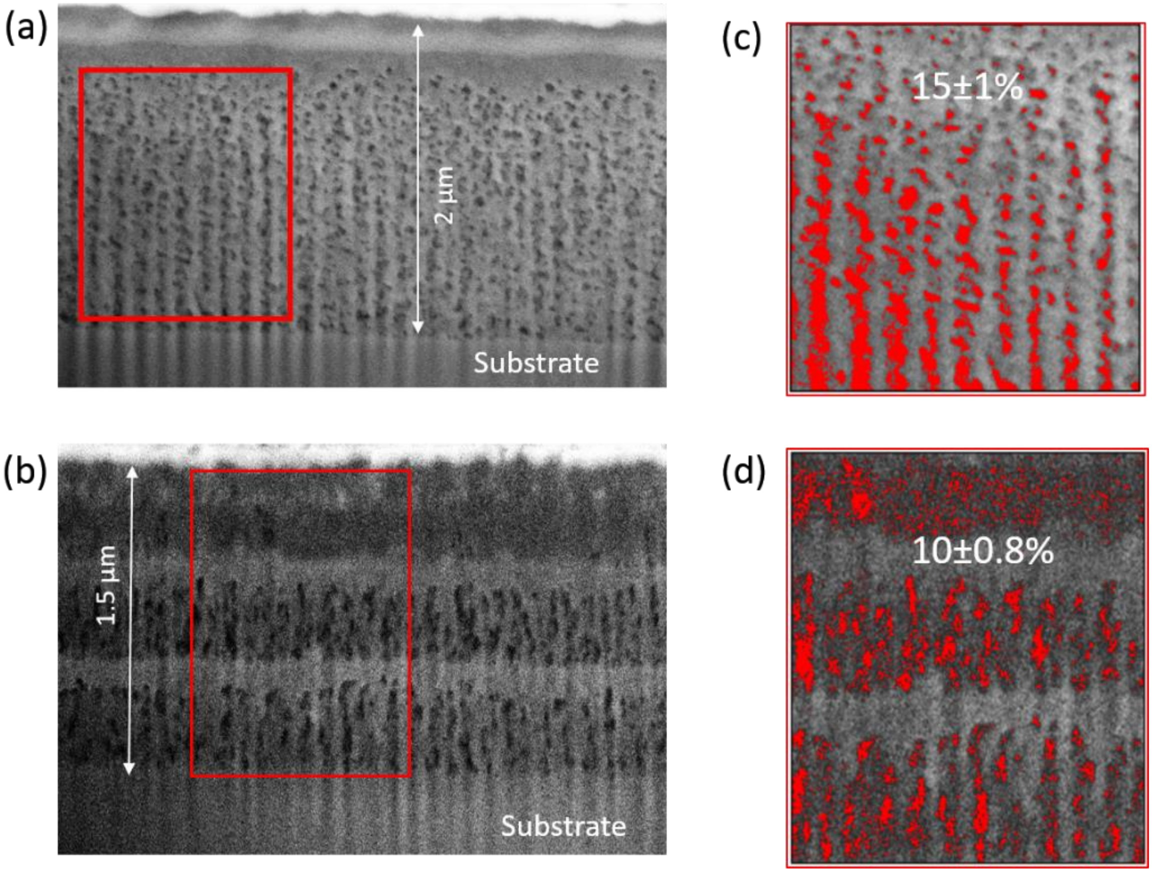

Figure S13. Cross-sectional SEM images of YBCO precursor TFA films obtained by Focused Ion Beam (FIB) with (a) a single layer using ink jet printing (IJP), (b) 3 layers using spin coating. (c) and (d) show the pore density analysis using ImageJ from the red rectangular region in (a) and (b), respectively, where pores are coloured in red for quantification.[4]

Section IV. Solution Rheological Analysis

Composition	Concentration (M)	Water content on day of preparation (wt%)	Viscosity (mPa·s)
(3:7)	1M(no additive)	0.23%	2.991 mPa·s
(3:7)	1M + 5%$_{v/v}$ MEA	0.37%	7.035 mPa·s
(3:7)	1.75M + 2%$_{v/v}$ MEA	0.74%	10.56 mPa·s
(3:7)	1.75M + 4%$_{v/v}$ MEA	0.78%	13.42 mPa·s
(3:7)	1.75M + 5%$_{v/v}$ MEA	1.033 %	19.85 mPa·s
(2:3)	1.75M + 2%$_{v/v}$ MEA	0.86%	13.05 mPa·s
(2:3)	1.75M + 4.3%$_{v/v}$ MEA	0.89%	17.4 mPa·s
(2:3)	1.75M + 5.5%$_{v/v}$ MEA	0.94%	19.35 mPa·s
(4:11)	1.75M + 2%$_{v/v}$ MEA	0.82%	9.78 mPa·s

Table S1. Rheological properties of various solutions used during the process of optimisation of the solutions.

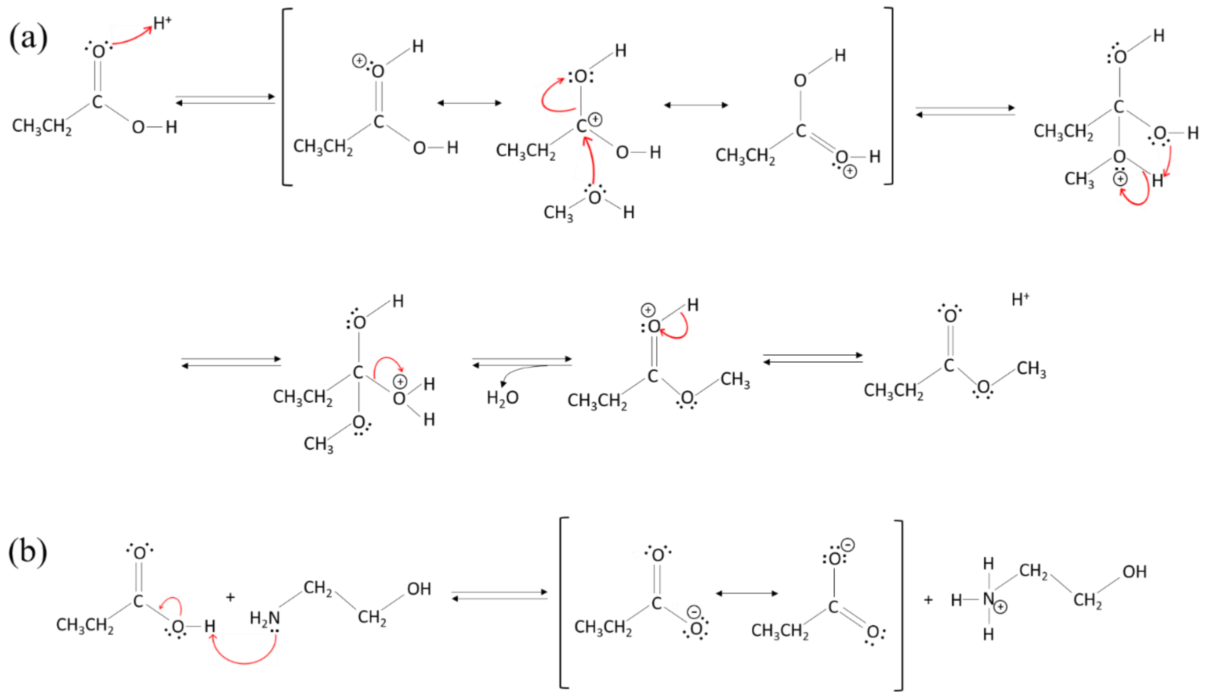

Figure S14. (a) Fischer esterification reaction mechanism for HProp and MeOH. Addition of an MEA to HProp reacts through the mechanism in (b).

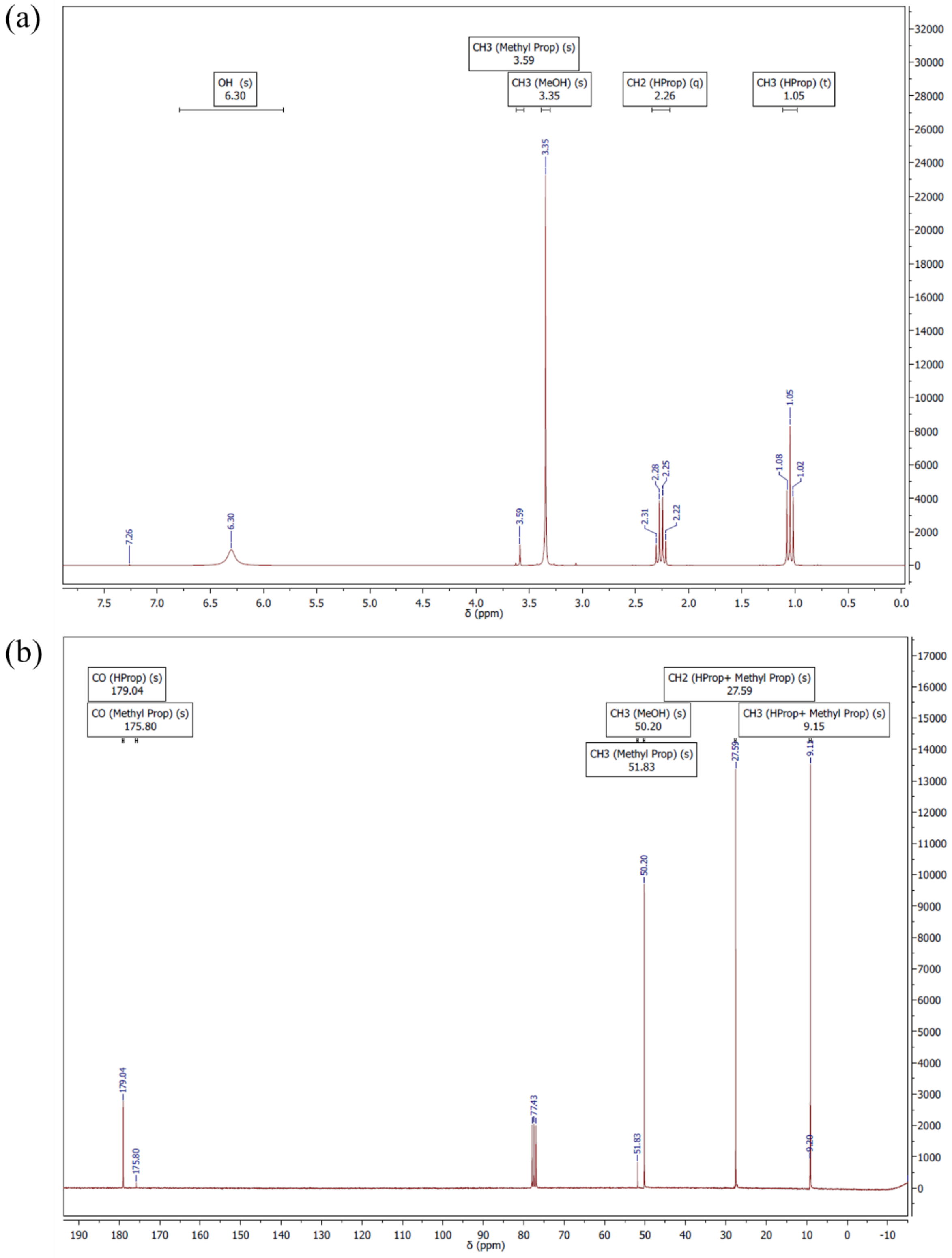

Figure S15. NMR spectra of mixture of solvents (HProp and MeOH in a 50:50 ratio) measured on day of preparation. (a) and (b) are, respectively, the ^{1}H spectrum and ^{13}C spectrum for the case with no amine additive. To be noted is the presence of the peak of methyl propionate, the ester formed from the Fischer esterification reaction of HProp and MeOH.

(a)

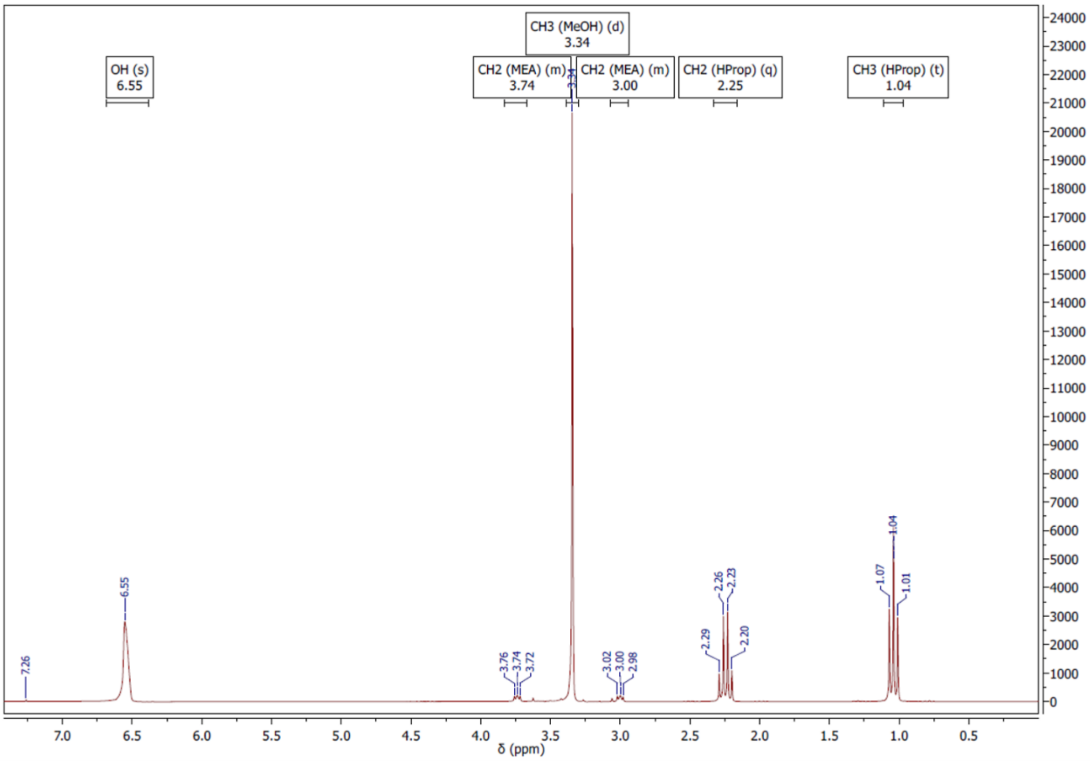

(b)

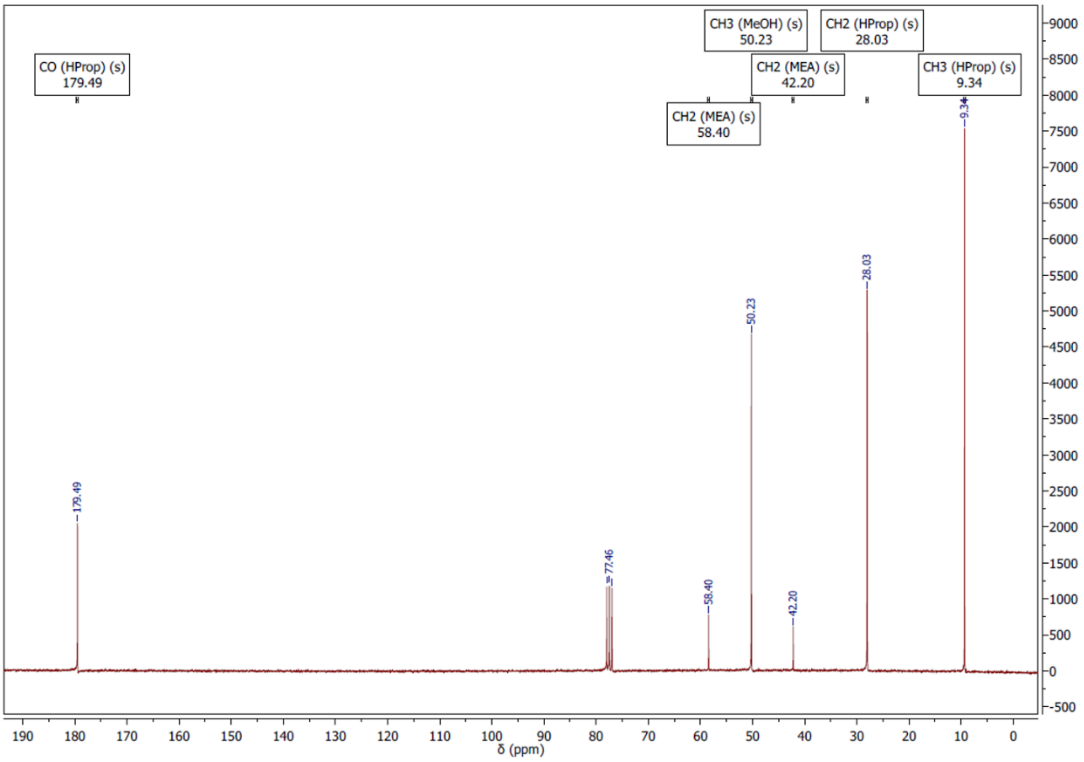

Figure S16. NMR spectra of mixture of solvents (HProp and MeOH in a 50:50 ratio) with the addition of MEA (4%$_{v/v}$) measured on day of preparation. Shown in (a) and (b) are, respectively, the ^{1}H spectrum and ^{13}C spectrum. Differently from Figure S13, in the case of MEA addition, no peak due to the presence of methyl propionate is found.

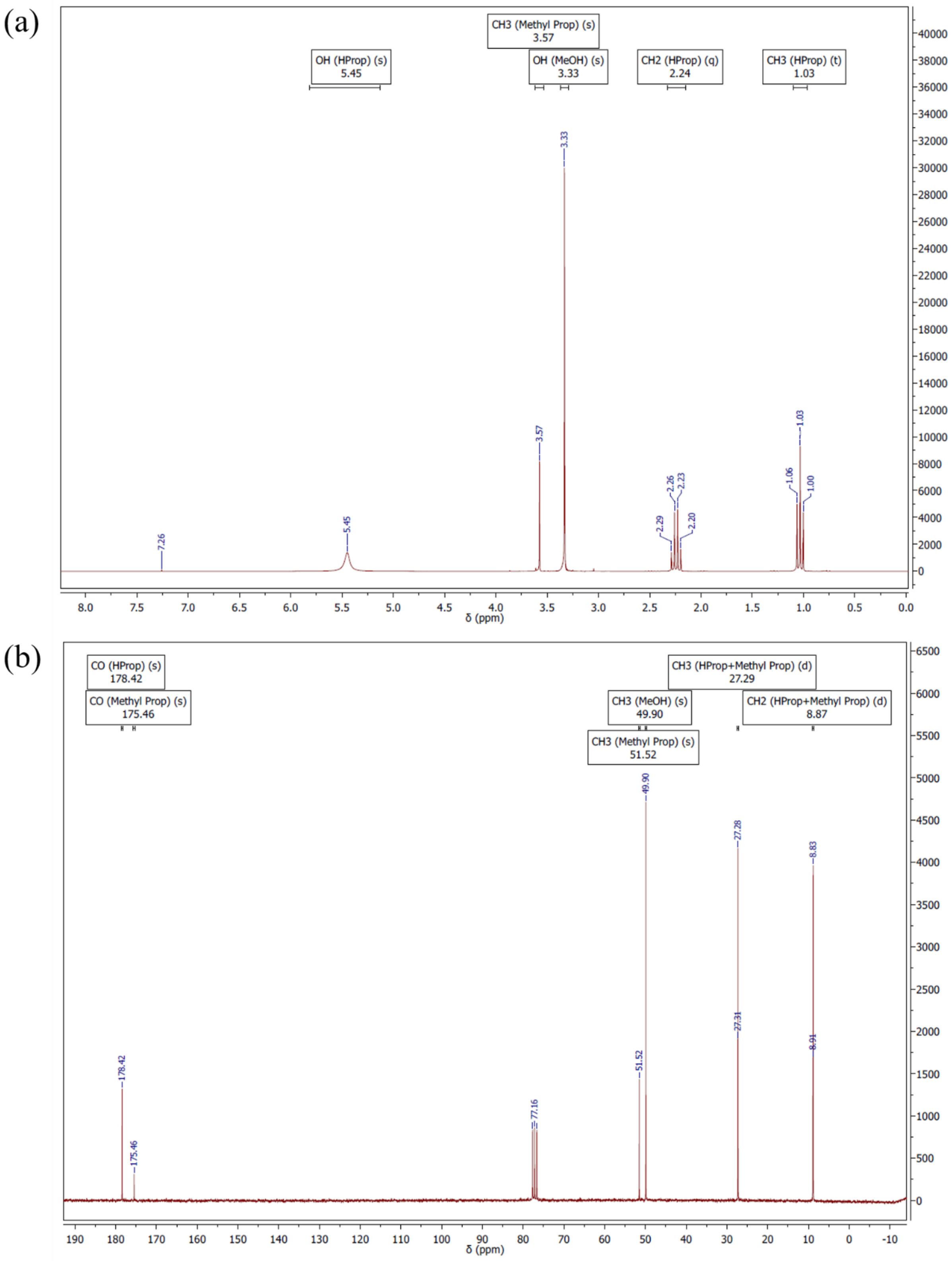

Figure S17. NMR spectra of mixture of solvents (HProp and MeOH in a 50:50 ratio) measured 8 days after its preparation. (a) and (b) are, respectively, the ^{1}H spectrum and ^{13}C spectrum for the case with no amine additive. The intensity of the peak due to methyl propionate has increased in intensity compared to the spectra from day of preparation of this solvents mixture.

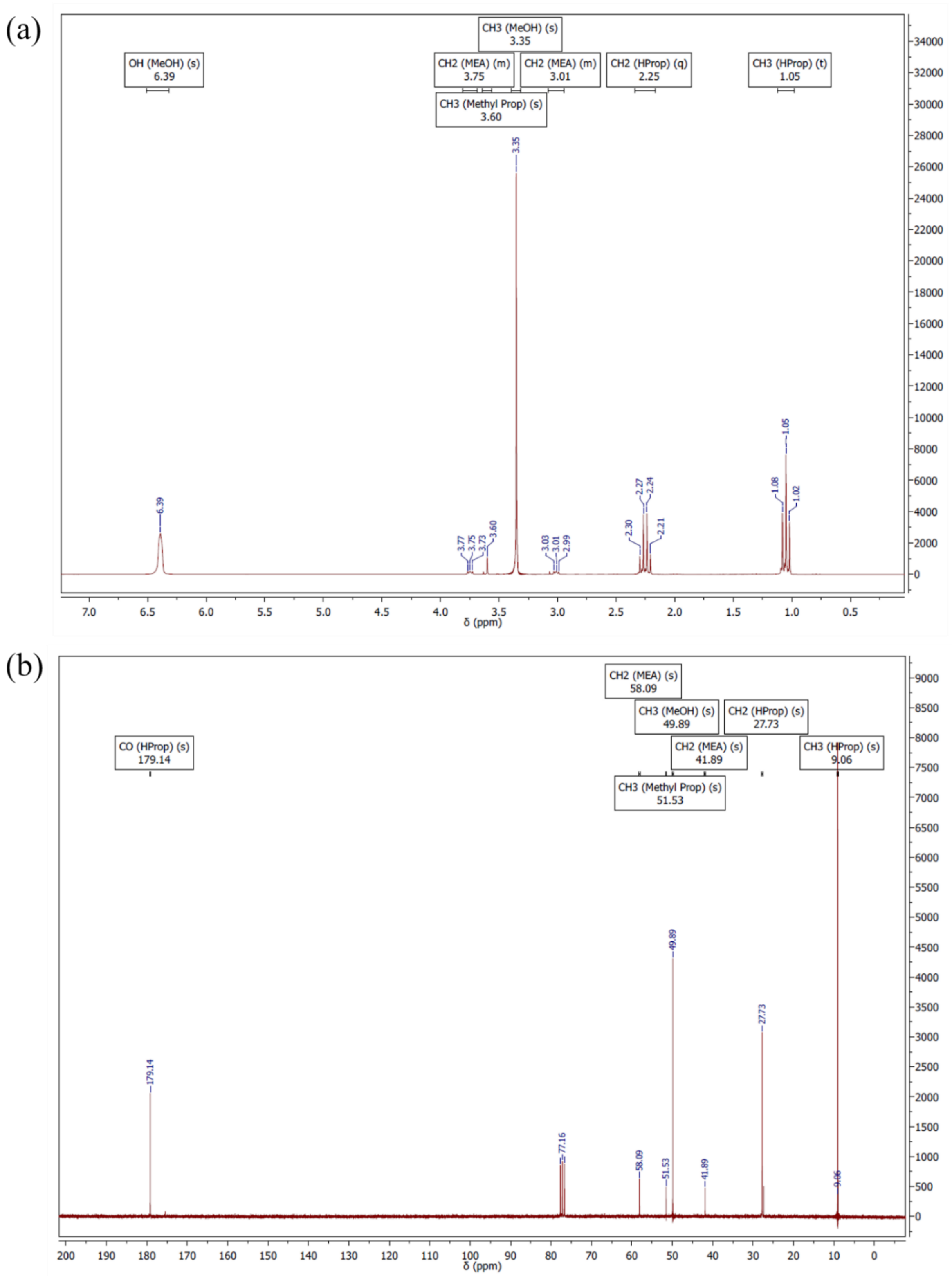

Figure S18. NMR spectra of mixture of solvents (HProp and MeOH in a 50:50 ratio) with the addition of MEA (4%$_{v/v}$) measured 8 days from its preparation. Shown in (a) and (b) are, respectively, the ^{1}H spectrum and ^{13}C spectrum. A peak due to the presence of methyl propionate is now visible, suggesting the occurrence of Fischer esterification reaction.

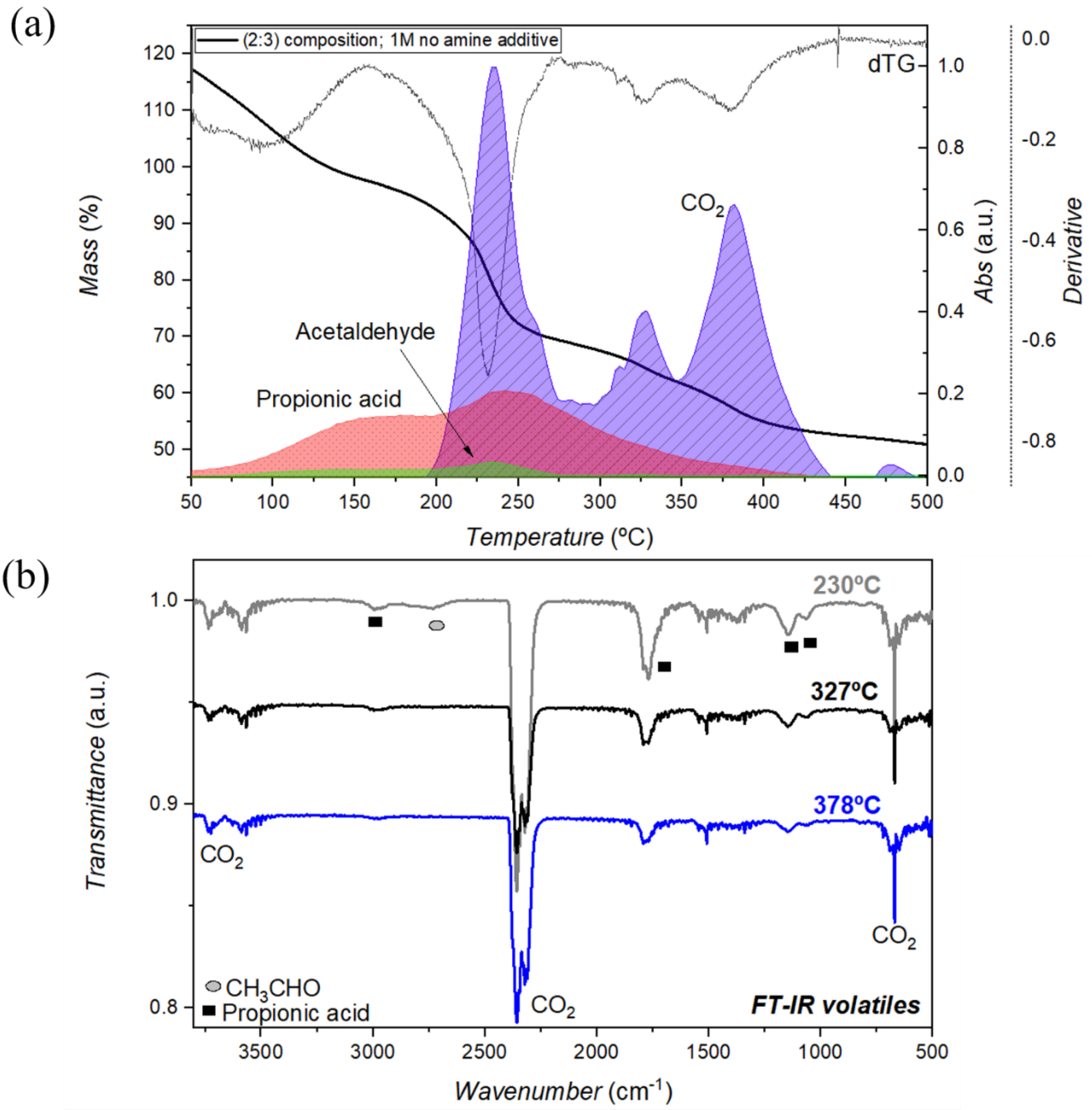

Figure S19. TGA analysis of (2:3) composition solution, 1 M no amine additive. Plot (a) displays the TG and dTG profiles together with the gas evolution during the heat treatment. Plot (b) shows the FT-IR corresponding to the temperatures where the mass losses are greatest. It can be noted that the largest mass loss shown in dTG corresponds to the temperature of 230-240 ºC. The evolution of volatiles identifies this loss as HProp and CO_2, coming from the first metal propionate to decompose, $Cu(Prop)_2$. The following mass losses in which CO_2 is liberated correspond to the decomposition of $Y(Prop)_3$ and, as last, $Ba(Prop)_2$.

(a)

(b)

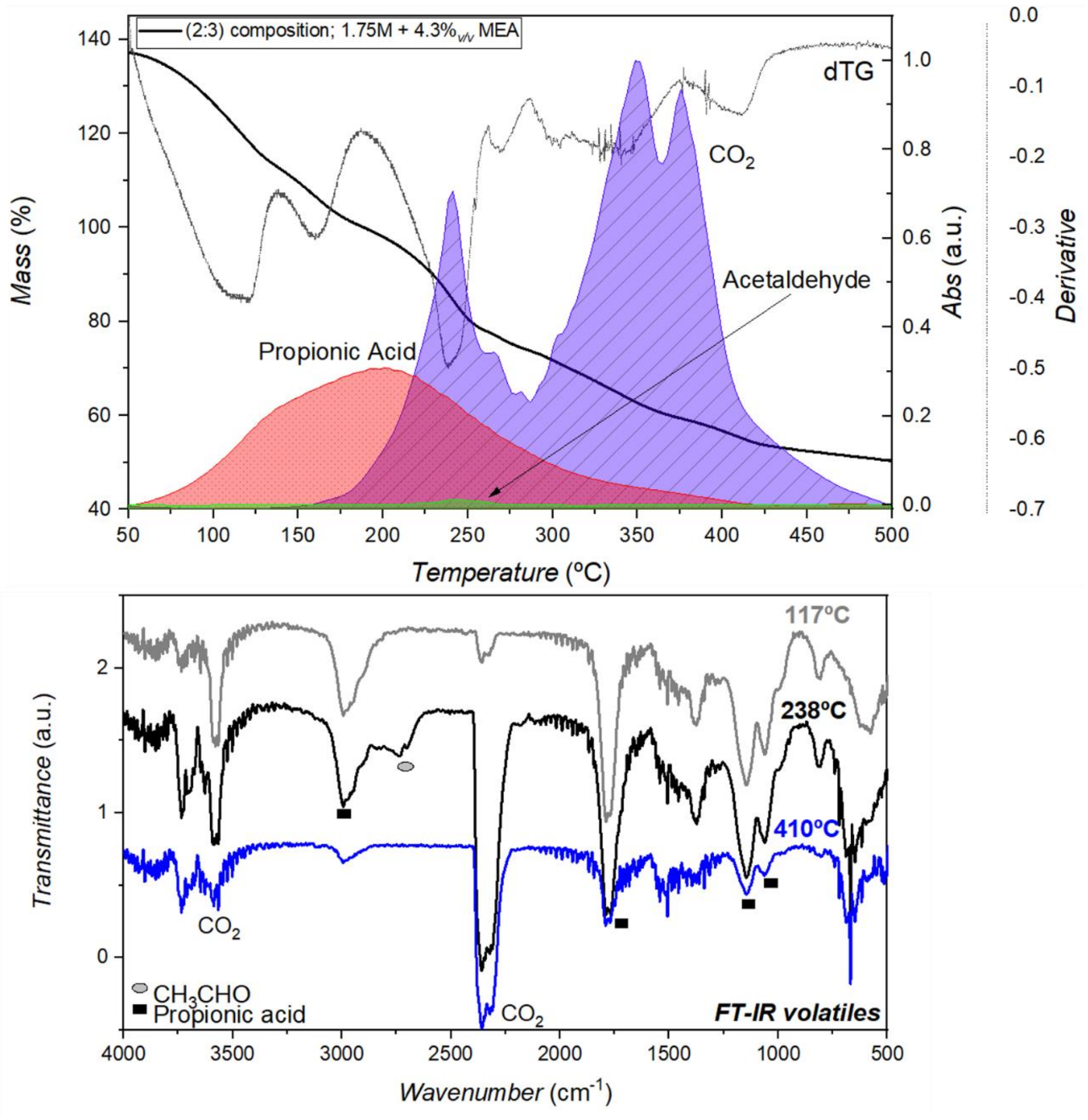

Figure S20. TGA analysis of (2:3) composition solution, 1.75 M + 4.3%$_{v/v}$ MEA. Plot (a) displays the TG and dTG profiles together with the gas evolution during the heat treatment. Plot (b) shows the FT-IR corresponding to the temperatures where the mass losses are greatest. In this case, in which MEA is added, the decomposition seems to be different than the one shown in Figure S17, where no MEA is employed. The largest mass loss shown in dTG still corresponds to the temperature of 230-240 ºC, but the difference between this mass loss step and the subsequent ones is less significant, resulting in a smoother TG curve profile. Notice that CO_2 loss is now distributed on a wider range at higher temperature. This might be the reason for which these solutions show such robustness to avoid crack formation, as it is favoured around 240 ºC[4]. The initial mass loss due to HProp is due to the high viscosity of this solution, impeding a successful drying of the samples before the start of the experiment.

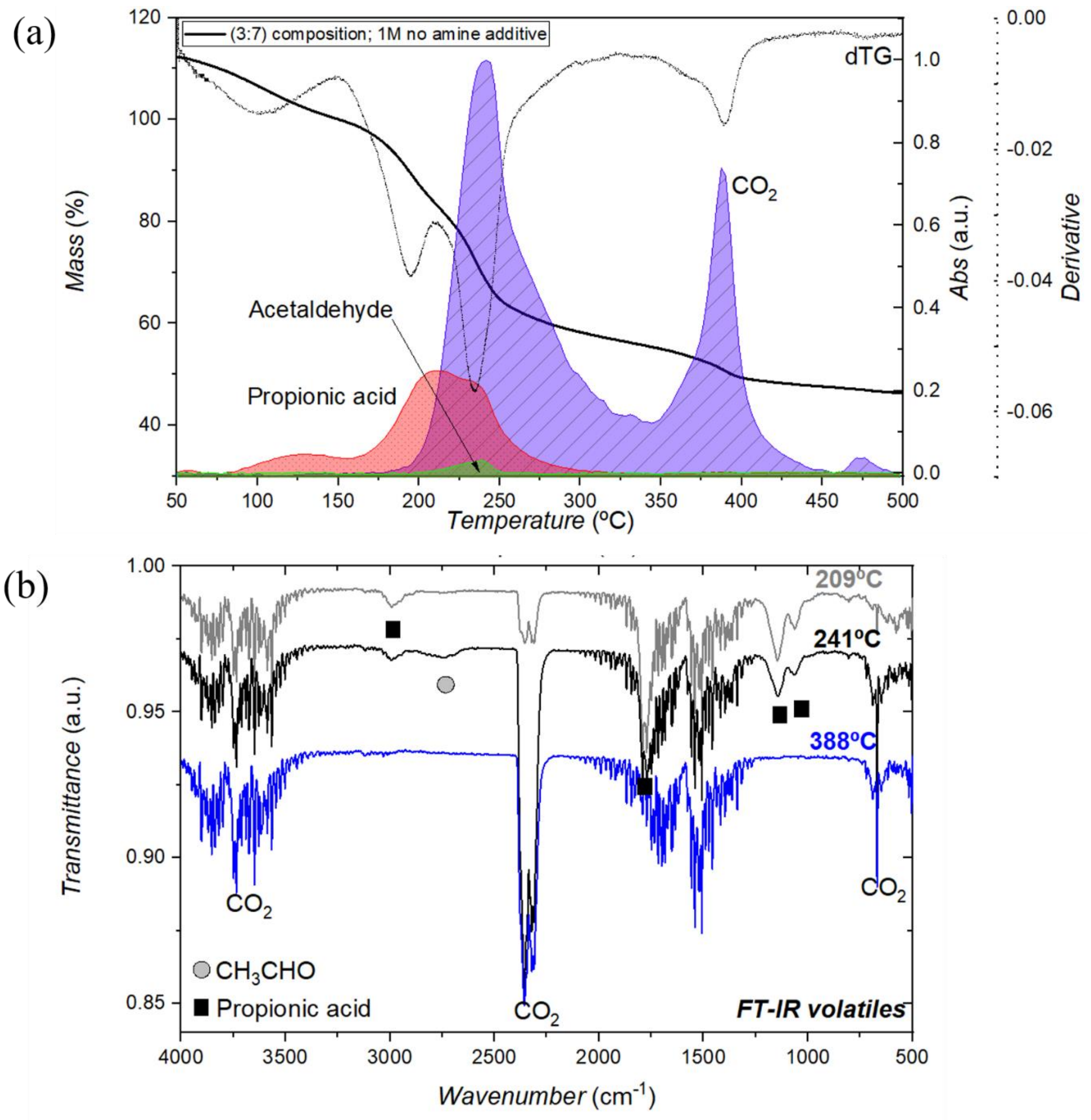

Figure S21. TGA analysis of a (3:7) composition solution, 1 M with no amine additive. Plot (a) displays the TG and dTG profiles together with the gas evolution during the heat treatment. Plot (b) shows the FT-IR corresponding to the temperatures where the mass losses are greatest. As in Figure S17, it can be noted that the largest mass loss shown in dTG corresponds to the temperature of 230-240 ºC. The evolution of volatiles identifies this loss as HProp and CO_2, coming from remaining $Cu(Prop)_2$ that already started to decompose before 200 ºC, and $Y(Prop)_3$. The following mass losses in which CO_2 is released correspond to the decomposition of $Ba(Prop)_2$.

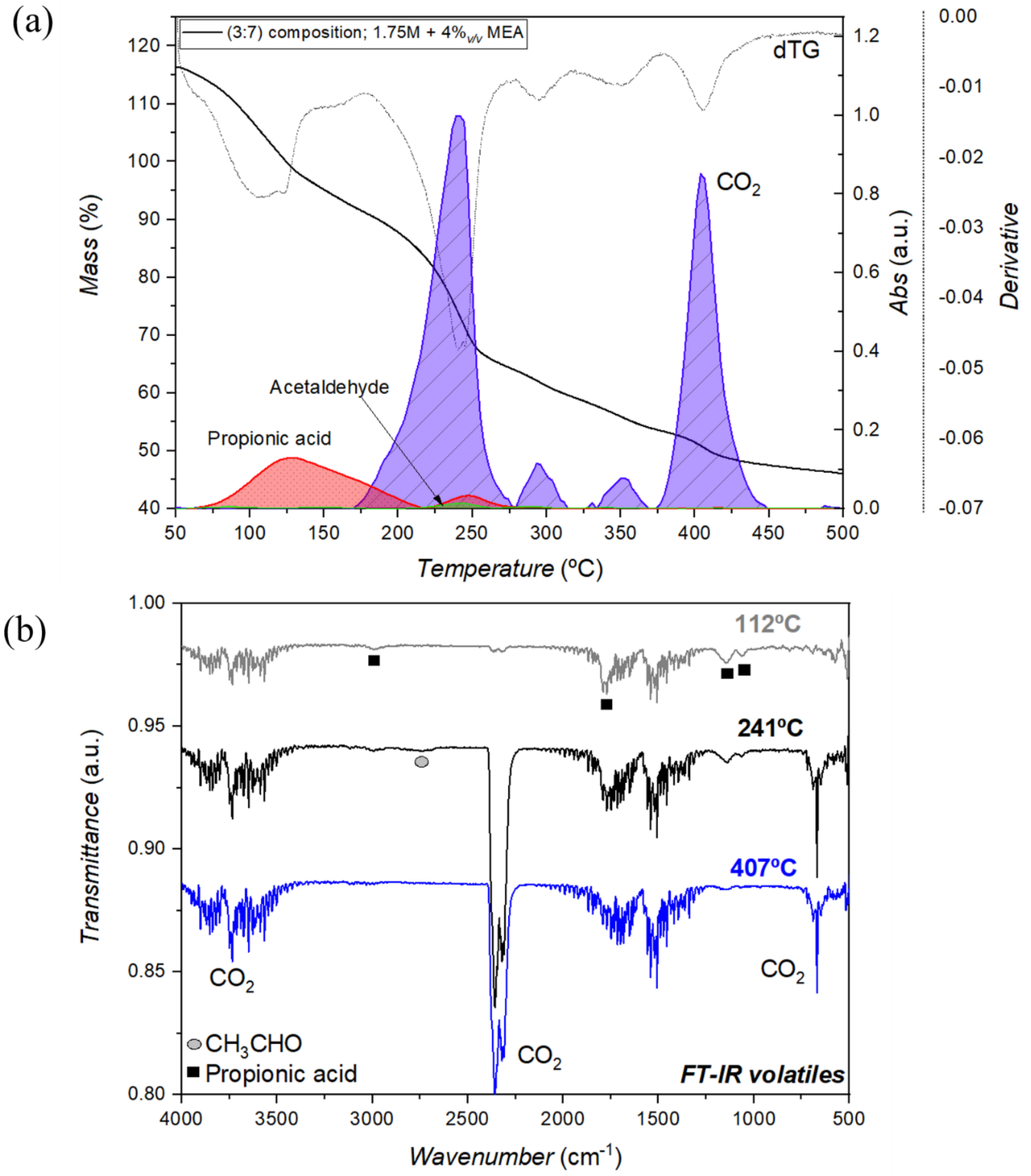

Figure S22. TGA analysis of (3:7) composition solution, 1.75 M + 4%$_{v/v}$ MEA. Plot (a) displays the TG and dTG profiles together with the gas evolution during the heat treatment. Plot (b) shows the FT-IR corresponding to the temperatures where the mass losses are greatest. In this case, in which MEA is added, the decomposition seems to be different than the one shown in Figure S17, where no MEA is employed. In this case, dTG shows an intense peak of CO_2 in the temperature of 230-240 ºC, however it seems to be comparable to the loss of CO_2 at higher temperatures, corresponding to the final decomposition of Ba(Prop)$_2$. The initial mass loss in the temperature range 100-200 ºC due to HProp is due to the high viscosity of this solution, impeding a successful drying of the samples before the start of the experiment.

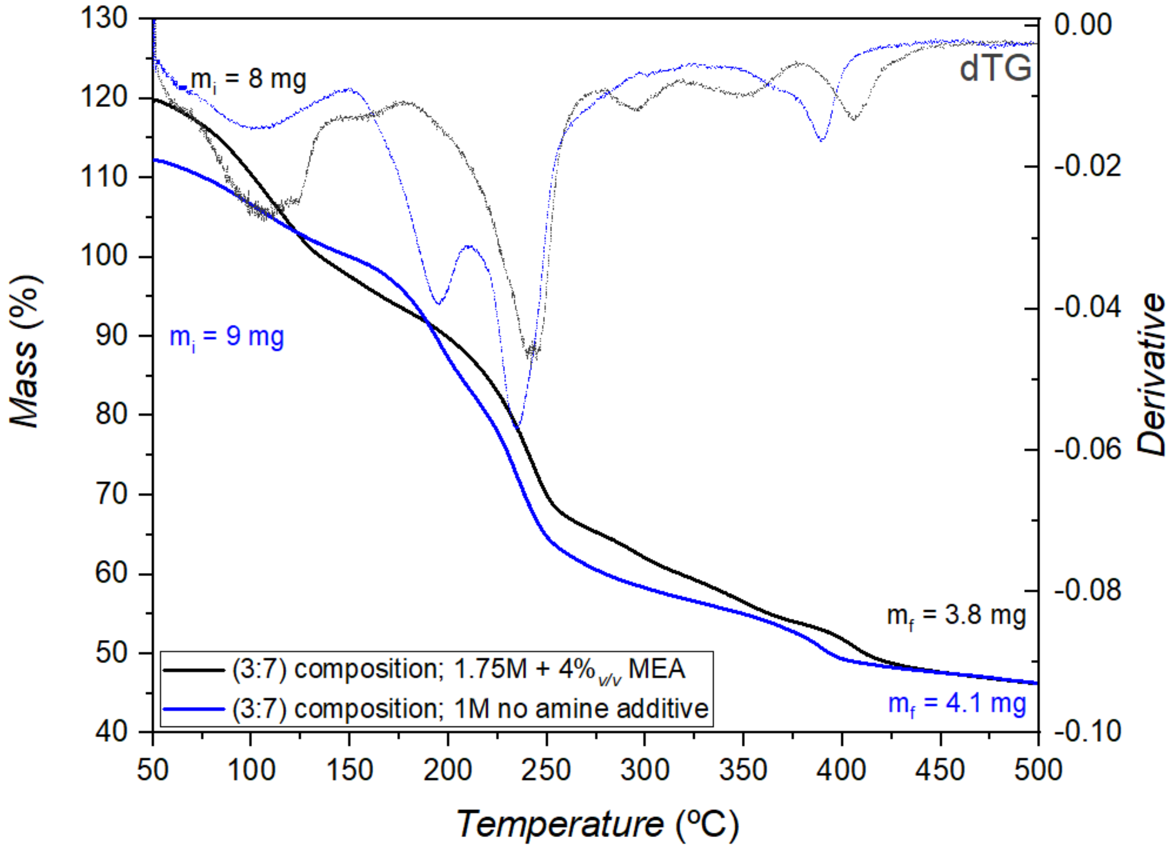

Figure S23. Comparison of TG and dTG of solutions with and without amine additive for (3:7) composition. Initial and final masses (m_i and m_f respectively) are displayed for a correct comparison of the samples, given the difference in concentration of the solutions. The main difference between the case with and without MEA is the smoother decomposition profile, specifically in the temperature range 230-240 ºC, the most delicate step as this corresponds to the moment in which crack formation is identified[4]. The case of a solution with no amine additive shows a greater evolution of CO_2 and HProp in this range, compared to the case in which MEA is added, possibly being this the reason for which the solutions with MEA are so robust and avoid completely the appearance of cracks when the optimal quantity is employed. Note also that the last decomposition steps are shifted to slightly higher temperatures.

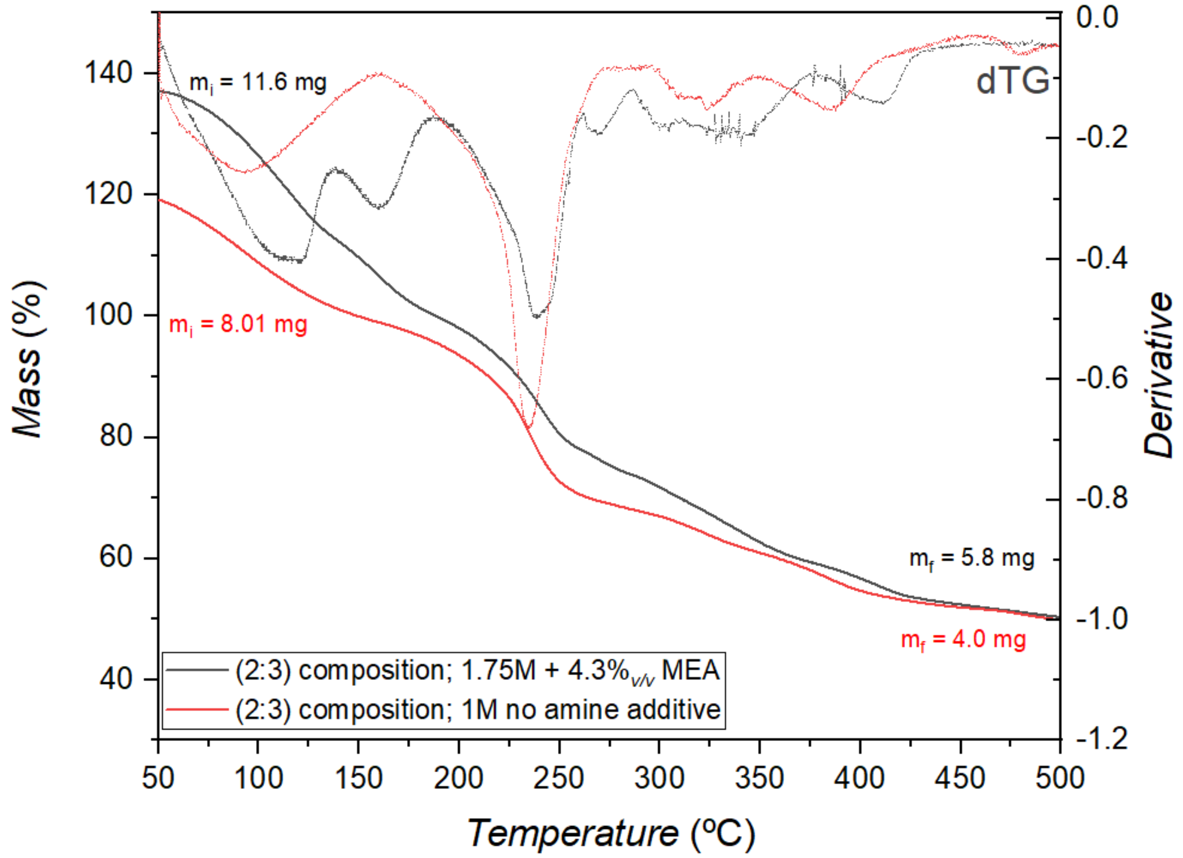

Figure S24. Comparison of TG and dTG of solutions with and without amine additive for (2:3) composition. Initial and final masses (m_i and m_f respectively) are displayed for a correct comparison of the samples, given the difference in concentration of the solutions. Similar to the case of (3:7) composition solutions, the main difference between the case with and without MEA is the smoother decomposition profile, specifically in the temperature range 230-240 ºC, the most delicate step as this corresponds to the moment in which crack formation is identified.[4] The case of a solution with no amine additive shows a greater evolution of CO_2 and HProp in this range, compared to the case in which MEA is added, which instead shows various peaks of decomposition of lower intensity in the range 50-280 ºC, possibly being this the reason for which the solutions with MEA are so robust and avoid completely the appearance of cracks when the optimal quantity is employed. Note also that the last decomposition steps are shifted to slightly higher temperatures.

Section V. YBCO Growth through TLAG

TLAG P_{O2} - route experiments are carried out in a tubular furnace equipped with a vacuum system that enables to switch fast (time range of seconds) from a low vacuum to a high vacuum. The P_{O2} is controlled by introducing gas lines in the vacuum system with regulating valves.

The samples are heated at low P_{O2} (10^{-5}-10^{-6} bar), with an average heating rate of 1 ºC/s to the desired temperature. The jump in P_{O2} follows, reaching the desired final P_{O2} in a time range of 1s. Cooling of the samples is performed with the same heating rate.

Following the TLAG process, an oxygenation process is performed for tetragonal to orthorhombic transition of YBCO. The samples are heated in a tubular furnace at a total pressure of 1 bar, with a heating rate of 10 ºC/min to 450 ºC, followed by a dwell of 210 min, then cooling with the same heating rate to RT. This process is performed under a continuous O_2 flow of 0.6 L/min.

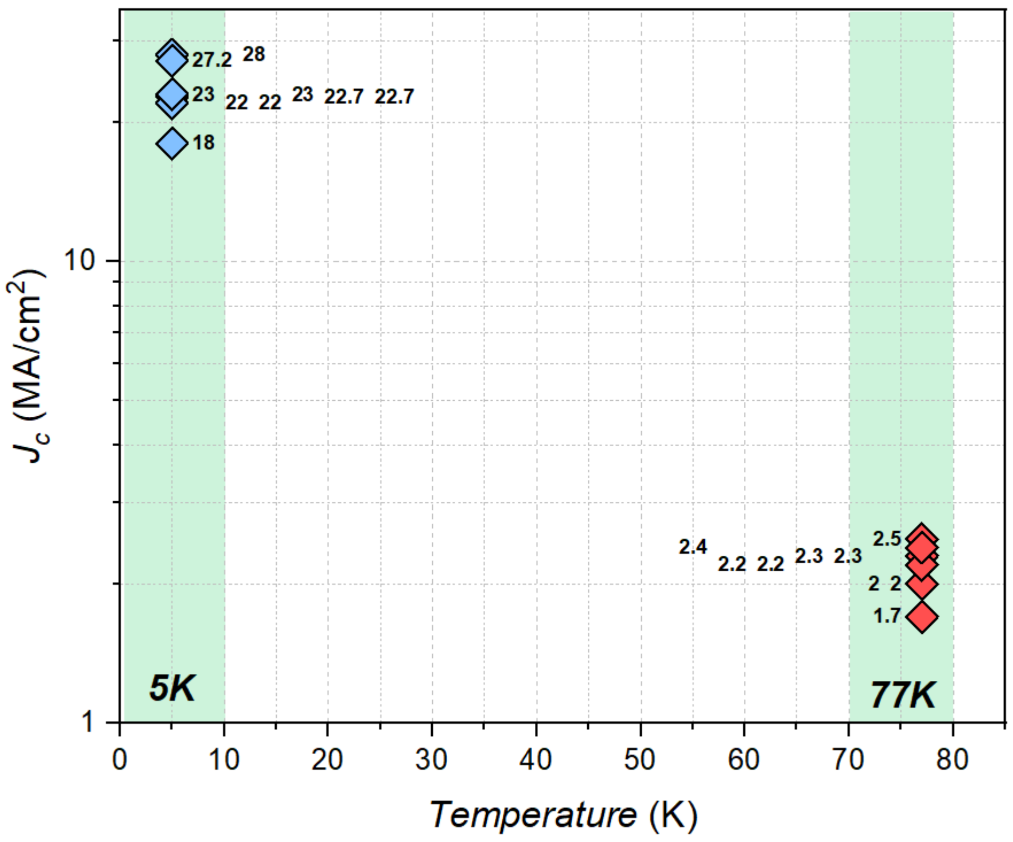

Figure S25. Plot showing the reproducibility of J_c values at 5K and 77K for 8 samples deriving from optimised solutions (1.75 M + 4%$_{v/v}$ MEA) of (3:7) composition grown through TLAG P_{O2} - route.

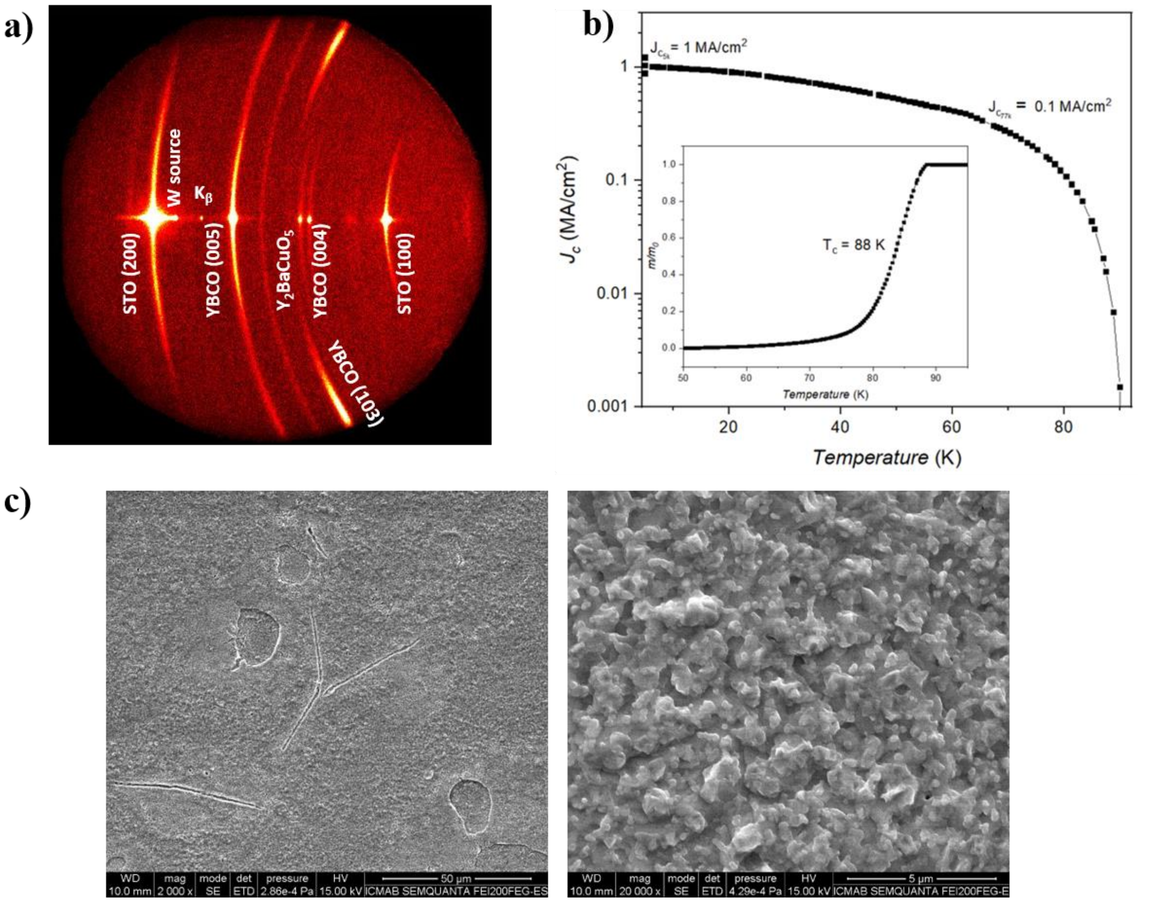

Figure S26. Results from the TLAG P_{O2} - route of a sample deriving from a solution 1.75 M + 8%$_{v/v}$ MEA (excess of MEA), as comparison to a sample obtained from an optimal solution (Figure 8 in main text), grown in the same conditions as sample shown in Figure 8. In (a) 2D-scan GADDS XRD shows the poor quality of the epitaxy in this sample, which displays an important polycrystalline character. This is reflected in the physical properties, J_c and T_c (as inset), shown in plot (b), being the value of J_c a factor 20 or more lower than the case of optimised samples. SEM images in (c) show a highly irregular surface, with no CuO (due to the (3:7) Cu excess) on the surface, in contrast to the case of optimal MEA quantity in Figure 8(b). The physical properties reported in (b) were measured with a commercial Quantum Design MPMS XL SQUID DC magnetometer equipped with a 7 T magnet. The critical current density has been calculated from the magnetization measurements using the Bean critical state model for a thin disk.

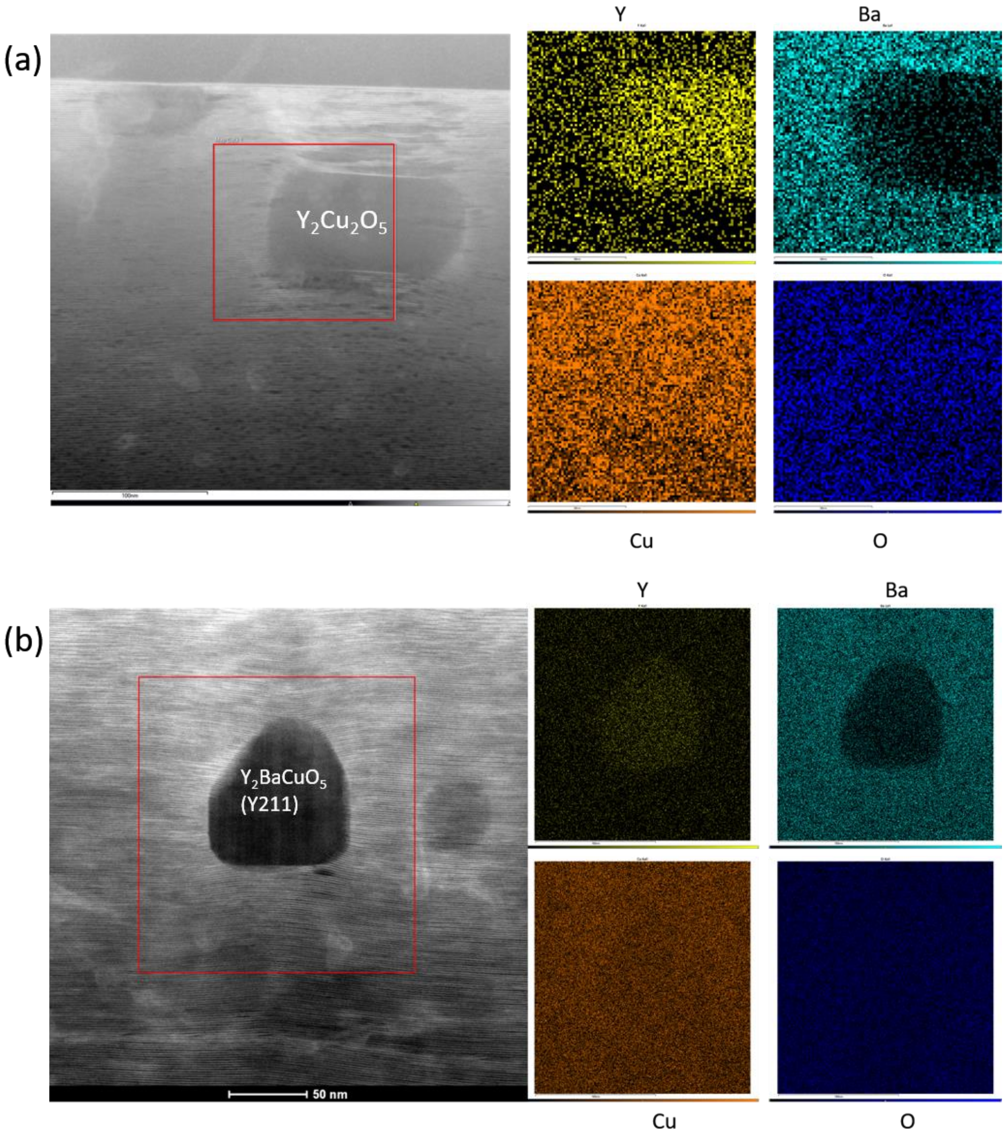

Figure S27. STEM-EDX cross-sectional elemental maps of Y, Ba, Cu, and O from different regions of a (3:7) composition YBCO thin film deposited using 1.75 M+4%$_{v/v}$ MEA solution, grown at 835 ºC, by performing a P_{O2} jump from 10^{-5} bar to 10^{-3} bar showing secondary phases of (a) $Y_2Cu_2O_5$ and (b) Y_2BaCuO_5 (Y211).

Notes and References

(1) Rasi, S.; Ricart, S.; Obradors, X.; Puig, T.; Roura-Grabulosa, P.; Farjas, J. Radical and Oxidative Pathways in the Pyrolysis of a Barium Propionate-Acetate Salt. *J. Anal. Appl. Pyrolysis* **2019**, *141*, 104640. https://doi.org/https://doi.org/10.1016/j.jaap.2019.104640.

(2) Rasi, S.; Ricart, S.; Obradors, X.; Puig, T.; Roura, P.; Farjas, J. Thermal Decomposition of Yttrium Propionate: Film and Powder. *J. Anal. Appl. Pyrolysis* **2018**, *133*, 225–233. https://doi.org/10.1016/j.jaap.2018.03.021.

(3) Rasi, S.; Silveri, F.; Ricart, S.; Obradors, X.; Puig, T.; Roura-Grabulosa, P.; Farjas, J. Thermal Decomposition of CuProp 2 : In-Situ Analysis of Film and Powder Pyrolysis. *J. Anal. Appl. Pyrolysis* **2019**, *140*, 312–320. https://doi.org/10.1016/j.jaap.2019.04.008.

(4) Villarejo, B.; Pop, C.; Ricart, S.; Mundet, B.; Palau, A.; Roura-Grabulosa, P.; Farjas, J.; Puig, T.; Obradors, X. Pyrolysis Study of Solution-Derived Superconducting $YBa_2Cu_3O_7$ Films: Disentangling the Physico-Chemical Transformations. *J. Mater. Chem. C* **2020**, *8* (30), 10266–10282. https://doi.org/10.1039/d0tc01846e.
